# A Combination of Chromatography with Tandem Mass Spectrometry Systems (UPLC-MS/MS and GC-MS/MS), Modified QuEChERS Extraction and Mixed-Mode SPE Clean-Up Method for the Analysis of 656 Pesticide Residues in Rice

**DOI:** 10.3390/foods10102455

**Published:** 2021-10-14

**Authors:** Thanh-Thien Tran-Lam, Minh Quang Bui, Hoa Quynh Nguyen, Yen Hai Dao, Giang Truong Le

**Affiliations:** 1 Institute of Mechanics and Applied Informatics, Vietnam Academy of Science and Technology, Ho Chi Minh City 72450, Vietnam; thanhthien307@gmail.com; 2 Center for Research and Technology Transfer (CRETECH), Vietnam Academy of Science and Technology, Hanoi 10072, Vietnam; bui_quang_minh@yahoo.com; 3 Institute of Chemistry, Vietnam Academy of Science and Technology, Hanoi 10072, Vietnam; hoanq8x@gmail.com (H.Q.N.); gianglt.hsmt@vast.vn (G.T.L.)

**Keywords:** pesticides, rice, QuEChERS, UPLC-MS/MS, GC-MS/MS, matrix effect

## Abstract

The emerging ungovernable application of pesticides in rice farming has attracted public concerns as these hazardous chemicals leave long-lasting environmental impacts and cause severe health effects. Here, an optimized analytical method was proposed for the measurement of 656 pesticide residues in rice samples collected in Vietnam. We utilized chromatography with tandem mass spectrometry systems (UPLC-MS/MS and GC-MS/MS) combined with a modified quick, easy, cheap, effective, rugged, and safe (QuEChERS) extraction method and adopted a mixed-mode SPE clean-up method for the analysis. The results showed that a total of 341 and 315 compounds were determined by UPLC- and GC-MS/MS, respectively. Usage of 10 mL MeCN, 5 mL H_2_O and 1% glacial acetic acid as extraction solvent outperformed other mixtures in purifying the analytes from the sample matrix. Besides, pressure swing adsorption connected to a C18 cartridge with C18 placed on top exhibited remarkably more extracted compounds of high recovery which resulted in 299 and 318 compounds with recovery ranging from 70 to 120% in GC- and UPLC-MS/MS, respectively. Our optimized protocols also resulted in maximal limits of quantification of 10 μg Kg^−1^ in both MS methods with repeatability and reproducibility less than 20%. Application of validated method on 20 rice samples collected in Hanoi, Vietnam showed that 14 samples were contaminated with at least one pesticide, and insecticide was the most detected group. Overall, the compliance of all method validation parameters to SANTE/12682/2019 Guideline demonstrates that this protocol can be employed for the effective management of Vietnam’s rice in accordance with international requirements.

## 1. Introduction

Rice provides approximately 20% of human daily calorie intake [1] as starch predominantly constitutes up to 80% of rice grain components, while other minor constituents such as proteins, lipids, fibers, and minerals make up 20% [2]. The humid tropical weather in South East Asia is ideal both for rice farming and the development of rice pests such as fungi, insects, and weeds that adversely affect crop productivity [3]. Therefore, numerous types of pesticides have been applied ungovernably as pest prevention and crop mitigation, and protection practices against pest infestation [4]. Inappropriate application of pesticides leads to long-lasting environmental impacts and severe health effects as residues of these poisonous chemicals enter the food chain and pollute the whole ecosystem [5,6]. Consequently, government agencies and international organizations have established maximum residue levels (MRLs) to regulate the level of pesticides detected in food. For instance, CODEX Alimentarius Commission set the general type-specific MRLs for pesticides in rice [7]. In addition, these MRLs are further adjusted by large rice-exporting countries such as China, India, Japan, the USA, and Brazil [3].

Liquid- (LC-MS/MS) and gas chromatography-tandem mass spectrometry (GC-MS/MS) are two arbitrating analytical methods for the examination of pesticide residues in food. These methods have been proven to provide better sensitivity and selectivity, greater limit of detection, and limit of quantification for simultaneous identification of multi-residues [8]. Besides the selection of appropriate analytical methods, the application of an efficient sample treatment for the complete extraction of analytes from complex food sample matrices is of great importance. Among the purification methods frequently employed, QuEChERS (quick, easy, cheap, effective, rugged, and safe) has gained more popularity owing to its major advantages and is considered as the gold standard for pesticide residues analysis in food [9]. Even though QuEChERS has proven its effectiveness for the extraction of a wide variety of analytes and sample matrices, the probability of co-extraction of contaminants cannot be left out which results in matrix effect, i.e., ion enhancement or suppression phenomenon. Consequently, the adoption of suitable sorbent in the clean-up stage plays a critical role in mitigating matrix effect, thus ensuring method sensitivity and selectivity as well as increasing result reliability [10].

This study describes a robust method using chromatography with tandem mass spectroscopy systems (UPLC-MS/MS and GC-MS/MS) in combination with QuEChERS extraction and mixed-mode SPE clean-up for simultaneously analyzing 656 pesticide residues in rice. The protocol was developed with the hope to provide an effective management tool for Vietnam’s rice quality in accordance with international requirements for exported items.

## 2. Materials and Methods

### 2.1. Chemicals

HPLC-grade acetonitrile (MeCN), methanol (MeOH), *n*-hexane, and toluene were purchased from Fisher (Waltham, MA, USA). Formic acid (>98% purities), ammonium formate (>99% purities), and glacial acetic acid (AA) were obtained from Sigma-Aldrich (St. Louis, MO, USA). The C18 SPE tube (40 µm, 500 mg, 6 mL) and the primary secondary amine (PSA) (40 µm, 500 mg, 6 mL) were purchased from Agilent Technologies (Santa Clara, CA, USA). The QuEChERS extraction tubes were composed of 4 g of anhydrous magnesium sulfate, 1 g of sodium chloride, 1 g of sodium citrate tribasic dihydrate, and 0.5 g of disodium citrate sesquihydrate purchased from Merck (Darmstadt, Germany) were prepared by weighting each substance into a 50-mL centrifuge tube. The mixture was then filtered through a polytetrafluoroethylene (PTFE) syringe filter (13 mm diameter, 0.22 µm pore size) provided by CNW (Shanghai, China).

### 2.2. Preparation of Standard Solutions

For LC-MS/MS analysis, 204 solution compounds were obtained from Restek (Bellefonte, PA, USA), and 137 solid compounds were obtained from Sigma-Adrich (St. Louis, MO, USA) and Dr. Ehrenstorfer (Augsburd, Germany) (Table S1). For GC-MS/MS analysis, 203 solution compounds were obtained from Restek (Bellefonte, PA, USA) and 112 solid compounds obtained from Sigma-Adrich (St. Louis, MO, USA) and Dr. Ehrenstorfer (Augsburd, Germany) (Table S1). For pesticide standard solutions, an intermediate standard solution at a concentration of 10 mg L^−1^ was diluted in MeCN solvent for LC-MS/MS analysis and in toluene solvent for GC-MS/MS analysis. All intermediate standard solutions were kept in amber dark glass vials stored in darkness at −20 °C to prevent photolysis. Working standard solutions at a concentration of 1 mg L^−1^ was prepared by diluting the stock intermediate standard solution in MeCN for LC-MS/MS analysis and in toluene for GC-MS/MS analysis and used for method optimization and method validation.

For GC-MS/MS analysis, alpha-BHC-d_6_ and parathion-d_10_ were provided by Dr. Ehrenstorfer (Augsburg, Germany) and used as labeled surrogates. The stock labeled- surrogate solutions were prepared in MeCN at 40 µg L^−1^ and used to monitor method validity. The working surrogate solutions were prepared in MeCN at 1 mg L^−1^ and stored in darkness at the temperature of −20 °C. The heavy-labeled surrogates were analytes chemically similar to those being extracted and added to a sample at a known concentration in order to determine the extraction efficiency of the sample preparation procedure. Trifluralin-d_14_ obtained from Dr. Ehrenstorfer (Augsburg, Germany) was used as an internal standard (IS) for GC-MS/MS. The IS stock and working solutions of 1 mg L^−1^ and 10 µg L^−1^ concentrations, respectively, were prepared in MeCN and stored in darkness at −20 °C. An internal standard (IS) was an analyte chemically similar to those being evaluated and was added to a sample at a constant concentration for calibration and quantitation purposes. The IS was typically added in GC-MS or GC-MS/MS methods for stabilization of analyte concentrations thorough the analytical process. Analyte protectants were used to eliminate the matrix effect in GC-MS or GC-MS/MS method. These compounds may strongly interact with active sites which were mainly free silanol groups in GC inlet and column and the mechanism may be similar to the interaction between matrix components and active sites. Therefore, the analyte protectants could eliminate the difference of the analyte signal between matrix-containing matrix-free solutions. The analyte-protectant (AP) compounds were 3-ethoxy-1,2-propandiol (0.2 g mL^−1^), D-sorbitol (5 mg mL^−1^), D-Gluconic acid δ-lactone (10 mg mL^−1^), and shikimic acid (5 mg mL^−1^). These protectants were dissolved in MeCN/H_2_O solvent (6/4, *v*/*v*).

### 2.3. Chromatographic Conditions

For LC-MS/MS analysis, the UPLC-MS/MS system was composed of a Dionex Ultimate 3000 UHPLC^+^ (equipped with a binary pump, an auto-sampler, and column thermostats) coupled to a TSQ Quantis triple quadrupole mass spectrometer (Thermo Scientific, Waltham, MA, USA). Additionally, an ACQUITY UPLC BEH C18 column (100 × 2.1 mm i.d. and 1.7 μm particle size) combined with an ACQUITY column in-line filter was used (Waters, MO, USA). The solvents employed for the mobile phase were MeOH and H_2_O, in which MeOH/H_2_O ratios of 2/98 (*v*/*v*) and 98/2 (*v*/*v*) were indicated as solvents A and B, respectively. Both solvents were added with ammonium formate 5 mM and formic acid 0.1% to stabilize the analyte form and retention time, as well as to enhance the signal during mass spectrometry analysis. The mobile phase was set up as follows: 0–0.5 min, 0% B; 0.5–3 min, 0–35% B; 3–23 min, 35–100% B; 23–23.1 min, 100–0% B; 23.1–25 min, 0% B. The curve parameter was set at 5 for each stage, but at 2 for the 0.5–3 min stage. The flow rate set for the gradient program was 0.3 mL min^−1^, and the sample injection volume was 5 μL. Temperatures of the column oven and auto-sampler were set at 40 °C and 4 °C, respectively.

Optimization of MS for each compound was conducted by directly injecting the standard of 1 mg L^−1^ at the rate of 20 µL min^−1^ in both positive and negative modes. This experiment was implemented to determine the fragmentor voltage and the collision cell energy for all the transitions. The optimization of MS was done by these following steps: (i) optimize the RF-lens parameters to obtain the maximum precursor ion intensity, (ii) optimize product ions to identify the optimized fragmentor voltages (ranging from 50 to 200 V), (iii) optimize collision cell energy (ranging from 1 to 50 V) for each fragment in single reaction monitoring (SRM). According to EU 657/2002/EC guideline for LC-MS/MS analysis, each compound is represented by two multi-reaction monitoring (MRM) transitions (the higher intensity for quantification, and lower intensity combined with MRM ratio for qualification). For the MS probe, nitrogen (>99.99% purity) was used as the sheath gas, sweep gas, and auxiliary gas at an airflow rate of 32, 2, and 12 arb, respectively. Sample vaporization and transfer-tube temperatures were 300 °C and 325 °C, respectively. The electrospray voltage settings at positive and negative modes were 3.5 kV and −2.5 kV, respectively. Mass spectrometric parameters are shown in Table S2.

For GC-MS/MS analysis, the GC-MS/MS system consisted of a Trace 1310 Thermo Scientific^TM^ gas chromatograph coupled with a TSQ 9000 triple quadrupole mass spectrometer (Thermo Fisher Scientific, Waltham, MA, USA) operated in the electron ionization mode (EI) of 70 eV. The analytes were separated on the silica-based capillary column DB-5 ms (30 m × 0.25 mm i.d. and 0.25 µm film thickness) provided by Agilent (Santa Clara, CA, USA) and connected to a 5-m pre-column of similar properties. The optimal PTV injector conditions are shown in Table S3. The chromatographic column temperature was established as follows: initial temperature of 70 °C in 2 min, increased from 70 °C to 150 °C at the rate of 8 °C min^−1^, held at 150 °C in 3 min, raised from 150 °C to 320 °C at the rate of 8.5 °C min^−1^, and held at 320 °C in 5 min. The total analysis time for a single GC/MS-MS run was 40 min. The splitless injection volume was 3 μL. Helium was used as carrier gas at the rate of 1.2 mL min^−1^. The purge nitrogen gas flow was set at 50 mL min^−1^. The two most intense transitions and their optimal collision energies were selected by the Thermo AutoSRM program, in which the highest signal-intensity product was assigned as the quantitative ion and the second-highest signal-intensity product as the qualitative ion. The mass spectrometric parameters are shown in Table S4.

### 2.4. Selection of Extraction Solvent

The selection of the extraction solvent can remarkably improve the purification of the analyte from the sample matrix [8]. Among the most prevalent solvents, MeCN has been commonly utilized in the QuEChERS extraction protocol since it can limit the influences of non-polar compounds such as fats, waxes, and pigments present in the sample matrix compared to other solvents [11]. Moreover, previous studies pointed out that the use of acetic acid considerably improves the extraction efficiency of the target analytes even at trace levels [12]. Moreover, the addition of acetic acid to MeCN exhibits the stability enhancement of several unstable pesticides [13]. Therefore, evaluation of extraction efficiency was carried out using three different solvent types: (i) 10 mL MeCN, (ii) 10 mL MeCN with 5 mL H_2_O and (iii) 10 mL MeCN with 5 mL H_2_O containing 1% acetic acid. Consequently, the third solvent mixture was used for subsequent analysis owing to a high abundance of compounds with high recovery ranging from 90 to 100%. Two g of blank rice sample (*n* = 3) was spiked at the pesticide level of 100 μg kg^−1^.

### 2.5. Selection of SPE Sorbent

QuEChERS has demonstrated good performance for the detection of pesticide residues in high carbohydrate-level, pigment-rich, and water content higher than 75% sample matrices [14]. Even though starch is the main component of rice grain, the minor proportion is composed of diverse compounds such as fatty acids, proteins, lipids, fibers, and minerals which is cumbersome for chromatographic analyses. Consequently, appropriate adoption of absorption material during the purification phase helps to minimize the matrix effect and prolong the longevity of the chromatographic column.

The most commonly used materials in the QuEChERS extraction protocol are PSA, GCB, and C18 [15]. These materials can strongly absorb organic components such as organic acids, pigments, proteins, fatty acids, and carbohydrates. PSA is mostly used for adsorbing polar compounds from the non-polar sample matrices such as fatty acids and carbohydrates [16]. However, it could not completely clean up the extracts, thus requiring the addition of C18 adsorbent to remove lipophilic co-extracts of the MeCN extract from food matrices [17]. Meanwhile, GCB is used to minimize the influences of fatty acid, pigments, and sterols in the sample matrix [18].

### 2.6. Optimization of Elution Volume

Elution volume is an important factor in the analytical procedure that directly affects the analytes’ recovery efficiency [19]. If the elution volume is insufficient, analytes would not be completely eluted from the adsorbent. On the other hand, over-sufficiency of the elution volume would cause the co-elution phenomenon of contaminants in the sample matrices. Here, four different elution volumes were examined to determine the optimal level: 10 mL (EV1), 15 mL (EV2), 20 mL (EV3), and 25 mL (EV4).

### 2.7. Sample Preparation

The pesticide-free organic rice coded BLPM2-CCP52 was obtained from Fapas (Sand Hutton, UK). Two g of rice sample was weighed in a 50-mL PTFE centrifuge tube, then added with 100 μL of surrogate 1 mg L^−1^. Then, 5 mL of deionized water and 10 mL of MeCN solvent containing 1% AA were added to the tube. The mixture was vortexed for 1 min, shaken in 15 min, and ultrasonicated in 30 min at 30 °C. Afterward, the QuEChERS mixture was directly added to the samples and immediately vortexed within 1 min to prevent the coagulation of MgSO_4_ and centrifuged at 7780× *g* in 10 min at 20 °C. Finally, 5 mL of the extraction aliquot was transferred to and cleaned up in the SPE cartridge.

The PSA and C18 SPE cartridges were preactivated with 5 mL MeCN containing 1% AA and joined together with a C18 cartridge placed on top using an adapter provided by Agilent (Santa Clara, CA, USA). Five mL of the extraction aliquot was loaded into the activated column system and 20 mL of MeCN:toluene solvent mixture (3/1, *v*/*v*) was used as the elution solvent. Both the extraction and the elution solvents were collected and concentrated to 2 mL using a centrifugal vacuum concentrator at 40 °C and 10 mbar in 50 min. The concentrated extraction aliquot was equally divided into two 15-mL tubes, each tube contained 1 mL of extraction aliquot. The extraction solvent in both tubes was evaporated to dryness using a nitrogen gas stream at the temperature of 1 °C. The remaining solid was re-dissolved by 1 mL MeOH:H_2_O (1/1, *v*/*v*) solvent, filtered through a 0.22-μm PTFE membrane, and analyzed by the UPLC-MS/MS system. The remaining pellet was dissolved by 0.98 mL of IS solution and 20 μL of AP solution before the GC injection to ensure a good signal and peak shape. After that, the sample was vortexed and analyzed by the GC-MS/MS system.

### 2.8. Method Validation

Method validation was performed in accordance with SANTE/12682/2019 Guideline for the following parameters: linearity, limit of quantification (LOQ), limit of detection (LOD), accuracy, precision, measurement uncertainty, and matrix effect [20]. Solvent calibration curves and matrix-matched calibration curves were generated using six calibration points for each curve at concentrations ranging from 5 to 200 μg L^−1^ in matrix-free solvent and matrix-containing solvent. Matrix-matched calibration curves were evaluated by the correlation determination R^2^ and used for quantification of the target analytes. LOQs were evaluated by the lowest spiked concentration level meeting the recovery and repeatability requirements within 70–120% and less than 20%, respectively, as regulated by SANTE/12682/2019 Guideline. LODs were estimated as one-third of LOQs. For method accuracy evaluation, recovery was measured using blank rice matrices at three concentration levels of 10, 50, and 100 µg kg^−1^ with five replicates at each level. The recoveries were calculated by dividing the ratio of the peak area of each analyte in the sample extract by the equivalent amount of the standard solution. Method precision was indicated by intra-day (RSD_r_) and inter-day relative standard deviations (RSD_R_). Regarding RSD_r_, samples fortified at three levels with six replicates at each level were analyzed within a day. The RSD_R_ was examined by analyzing the samples of two consecutive days. For ESI ionization determination, the matrix effect was evaluated by comparing the slope of the matrix-matched calibration curves with one of the solvent calibration curves. The matrix effect was calculated using the following formula:

(1) $$\mathrm{ME}=\left(\frac{\mathrm{slope}\;\mathrm{of}\;\mathrm{the}\;\mathrm{matrix}-\mathrm{matched}\;\mathrm{standard}}{\mathrm{slope}\;\mathrm{of}\;\mathrm{the}\;\mathrm{solvent}\;\mathrm{standard}}-1\right)\times 100$$

The effect is mild or tolerable when ME ranges from −20% to 20%, medium when ME falls between −50 to −20% or 20 to 50%, and strong when ME is lower than −50% or greater than 50% [21].

### 2.9. Method Application on Rice Samples

We applied the optimized method to analyze the presence of pesticide compounds in 20 commercial ordinary rice samples collected from June to August, 2020 in several markets around Hanoi, Vietnam. Those rice samples were harvested in lowland paddies in Northern Vietnam.

## 3. Results and Discussion

### 3.1. Selection of Extraction Solvent

**Figure 1:**
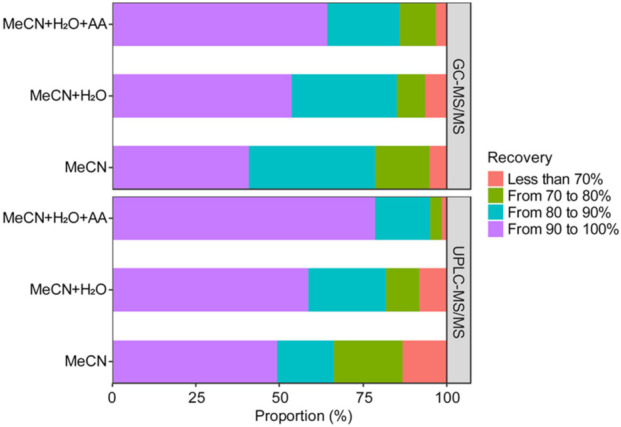
Comparision of the proportion of compounds obtained at different recoveries by using different extraction solvents (UPLC-MS/MS: 341 compounds, GC-MS/MS: 315 compounds).

The proportion of pesticides discovered by UPLC- and GC-MS/MS categorized into four recovery ranges is shown in Figure 1. Overall, 341 and 315 compounds were extracted by the latter and the former, respectively. Notably, usage of MeCN + H_2_O + AA solvent yielded best results in both MS methods, represented by the abundance of compounds with recovery ranging from 90 to 100 of 80% in UPLC-MS/MS and 70% in GC-MS/MS. The MeCN + H_2_O solvent closely followed that with approximately 60% of compounds of high recovery in both methods. Meanwhile, MeCN demonstrated underperformance compared to the abovementioned solvents with less than 50% of compounds belonging to the high recovery range. In contrast, there were more compounds of less than 70% recovery range extracted by MeCN than by MeCN + H_2_O + AA in both MS methods. Such results suggest that adding acetic acid to the extraction solvent considerably improved the extractability of the analyte from the sample matrix. Therefore, a mixture of 10 mL MeCN, 5 mL H_2_O, and 1% AA were selected as the extraction solvent employed in this study.

### 3.2. Selection of SPE Sorbent

**Figure 2:**
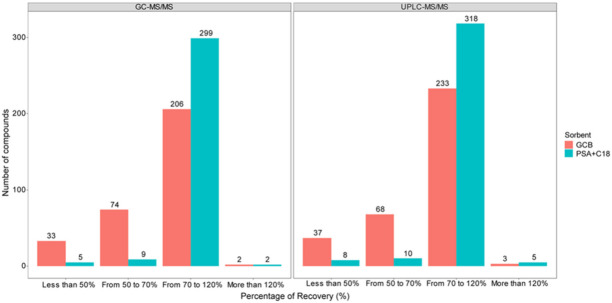
Comparision of the number of compounds obtained at different recoveries by using different SPE sorbents (UPLC-MS/MS: 341 compounds, GC-MS/MS: 315 compounds).

The number of analytes in response to different types of SPE sorbent analyzed by UPLC-and GC-MS/MS systems is displayed in Figure 2. GCB and PSA+C18 resulted in a similar number of compounds of high recovery in GC- and UPLC-MS/MS. Nonetheless, the number of compounds with recovery ranging from 70 to 120% adsorbed by PSA+C18 was remarkably higher than by GCB in both MS systems. In particular, 299 and 318 compounds were extracted by PSA+C18 material whereas 206 and 233 were extracted by GCB material in GC- and UPLC-MS/MS, respectively. Analytes such as carbohydrates, lipids, and fatty acids are strongly adsorbed by PSA+C18 material with weak chemical interactions, thus easily eluted by MeCN sorbent with high recovery efficiency. In contrast, GCB possesses a strong affinity towards aromatic planar compounds such as pesticides compounds [22]. As a result, elution of compounds such as carbendazim, thiabendazole, cyprodinil, diflubenzuron, or teflubenzuron was more cumbersome, resulting in recovery lower than <70% and higher when using GCB compared to PSA+C18 materials. As a consequence, the combination of C18 and PSA SPE cartridges was most relevant for the analysis of the rice sample matrix.

### 3.3. Optimization of Elution Volume

**Figure 3:**
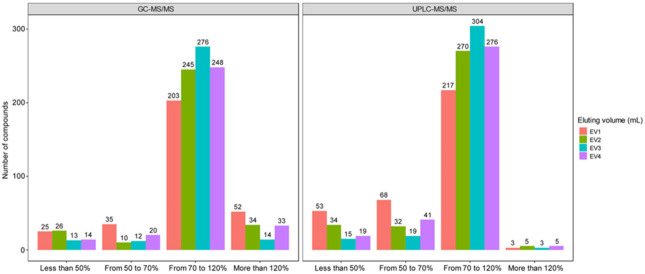
Comparision of the number of compounds obtained at different recoveries by using different elution volume (UPLC-MS/MS: 341 compounds, GC-MS/MS: 315 compounds). EV1: 10 mL, EV2: 15 mL, EV3: 20 mL, and EV4: 25 mL.

Elution volume strongly influenced the recovery of the analytes as shown in Figure 3. Accordingly, most compounds of high recovery efficiency ranging from 70 to 120% were eluted with 20 mL of elution solution, in which 276 compounds were identified by GC- and 304 compounds by UPLC-MS/MS method. Using less elution resulted in a remarkably lower number of analytes. In particular, 10 mL of solvent eluted 60 pesticides of less than 70% recovery as analyzed by GC-MS/MS, and 121 pesticides of less than 70% recovery as analyzed by UPLC-MS/MS. This was due to the incapability of complete elution of analytes by the SPE sorbent. However, further increase in elution volume to 25 mL did not yield better results than 20 mL, not to mention produced fewer compounds in both MS systems. This was due to the influence of co-eluents present in the sample matrix which was caused by ion suppression in UPLC-MS/MS analysis or ion enhancement in GC-MS/MS analysis. Therefore, 20 mL of ACN/toluene = 3/1 (*v*/*v*) was selected for the SPE clean-up process.

### 3.4. Method Validation

The results of parameter validation including linear range, coefficient of determination, accuracy (RSD_r_) and reproducibility (RSD_R_), limit of determination, limit of quantification, and matrix effect are displayed in Table S2 for UPLC- and S4 for GC-MS/MS. Linear regressions of all analytes exhibit coefficient of determination greater than 0.999, which is an indicator of excellent goodness-of-fit for the calibration points (Table 1, Tables S2 and S4). Regarding UPLC-MS/MS analysis, 305, 29, and 7 compounds had linear ranges from 1, 2, and 5 to 200 μg L^−1^, respectively. Regarding GC-MS/MS analysis, 68, 37, 178, and 30 compounds had linear ranges from 1, 2, 5, and 10 to 100 μg L^−1^, respectively. All analytes displayed recovery between 70 and 120% with RSD_r_ and RSD_R_ less than 20%. Furthermore, the maximal LOQ was 10 μg Kg^−1^, which strictly followed the MRL regulated by the EU for rice products [23]. Particularly, the lowest limit quantified by the GC system was 1 μg Kg^−1^, and most analytes were detected at the concentration of 5 μg Kg^−1^ (178 out of 315 compounds). Meanwhile, the UPLC system started recognizing pesticide residues at 2 μg Kg^−1^, which was also the concentration that most compounds were measured (302 out of 341 compounds). These results confirmed the robustness of our analytical procedure in accordance with SANTE/12682/2019 Guideline.

### 3.5. Matrix Effect

**Figure 4:**
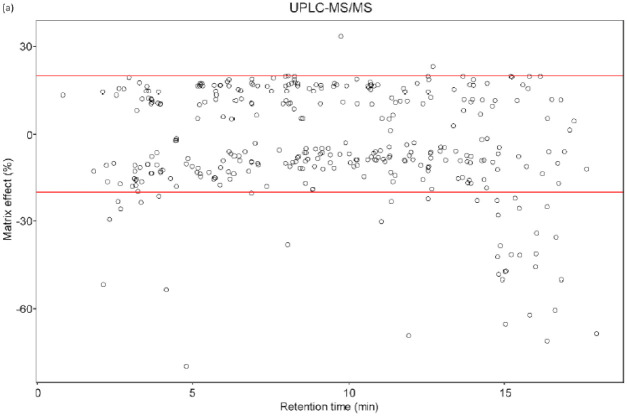

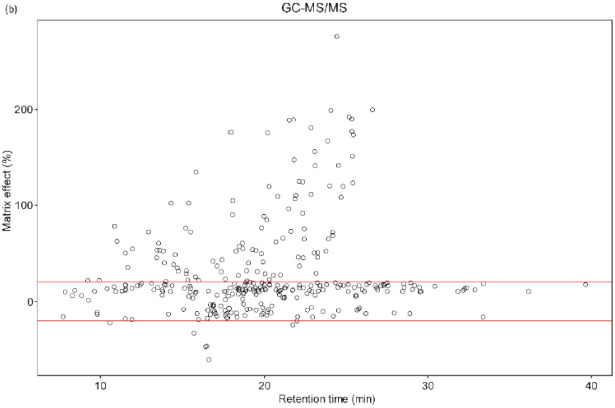
Correlation between retention time and matrix effect in (**a**) UPLC-MS/MS and (**b**) GC-MS/MS systems.

QuEChERS extraction method combined with PSA+C18 absorbents could effectively minimize the sample matrix, thus increasing the reliability of the analytical result (Table 2, Tables S2 and S4). In this study, two observed opposite ion fluctuation phenomena require careful consideration: ion suppression in UPLC- and ion enhancement in GC-MS/MS (Figure 4). In liquid chromatography, most pesticides are in the soft effect zone and only 38 out of 341 compounds were affected by the sample matrix, which generally provided better results than previously reported. Lee et al. [24] proposed a method for the determination of 47 pesticides in polished rice using LC-MS/MS combined with ultrasonic extraction, in which nine out of 41 compounds were affected by the matrix effect. Besides, Takatori et al. [17] reported matrix effect in 13 out of 99 pesticide compounds in rice using LC-MS/MS combined with C18/GCB/PSA SPE sorbent.

Ion suppression was commonly detected in UPLC-MS/MS [25]. Analyte ionization efficiency of the ESI ionization mode may be affected by the sample matrix containing droplets of fluctuating viscosity due to contaminants distributed on the droplet’s surface [21]. Besides, the octane-water ratio may also contribute to the ion suppression phenomenon as 38 analytes had logK_o/w_ values ranging from 1.9 to 6.0. Therefore, it can be concluded that the background effect mildly affected UPLC-MS/MS analysis result.

On the contrary, the ion enhancement phenomenon is more commonly detected in GC-MS/MS analysis [21], which is observed by the increase in analytical signals of 104 in 313 compounds compared to those of the standard solutions. Such phenomenon was mainly seen in compounds of short retention times (t_R_ < 27 min), whereas those at the end of the heating program were almost unaffected by the sample matrix. This was also higher than the results reported by Lee et al. [26] using GC-MS/MS combined with the QueChERS-dSPE method. Erney et al. [27] ascribed ‘matrix-induced chromatographic response enhancement effect’ as the main cause of ion enhancement event. Specifically, non-volatile matrix components in the sample matrix can be easily accumulated in the GC inlet, liner, or front part of an analytical column by repeated injections, which easily gives rise to the successive formation of new active sites. At the same time, this reduces the analyte absorption on the active sites as well as minimizes thermal decomposition of the thermal-sensitive compounds, making it easier for the compounds to reach the MS probe [28].

Pesticide compounds containing polar functional groups such as carboxyl, carbamate, phosphate, amine group or urea group are easily affected by the sample matrix because these functional groups probably interact with the silanol group or metal ions in liner or glass wool [29,30]. Those groups strongly affected by rice sample matrix are phosphorothiolate (e.g., pyryzophos), pyrethroid (e.g., etofenprox, permethrin), organophosphate (e.g., fenthion, sulprofos, pyridaphenthion, phosalone, coumaphos), and imidazole (e.g., prochloraz). The organochloride group is less affected by the rice matrix because the structures of these compounds have virtually no interaction with the active sites such as the silanol group on the liner surface [30]. Besides, we noticed that the pyrrole, pyrimidine, or uracil groups exhibited strong matrix-induced enhancement effects in all rice samples. Despite the thorough selection of the purification solvent material for the clean-up process, the sample matrix effect cannot be completely excluded but only mitigated using numerous methods, in which a matrix-matched calibration curve appears to be the best solution [31].

### 3.6. Method Application on Rice Samples

Results of method application on commercial ordinary rice samples are illustrated in Table 3. In general, a total of 24 pesticides out of 656 compounds were detected, which were categorized into four main groups: herbicide, insecticide, fungicide, and others. Insecticide was the most detected group with 18 out of 24 compounds found in 20 rice samples, whilst fungicide and herbicide were the least discovered group with four and three compounds present in rice samples, respectively. Within the insecticide group, lambda-cyhalothrin with a concentration ranging from 1.7 to 33.3 µg Kg^−1^ was the most detected compound which was found in 14 out of 20 rice samples and the detection limitation exceeded the regulated insecticide threshold in three samples. Nonetheless, the level of lambda-cyhalothrin determined in this study was lower than the concentration of 0.11 mg Kg^−1^ of a Korean rice sample recorded by Dong, et al. [32]. This compound is mainly used to prevent *Chilo suppressalis*, a serious rice pest that causes rotation in rice stems [33]. Besides lambda-cyhalothrin, chlorpyrifos and bifenthrin are two highly occurred compounds in the rice samples with concentrations ranging from 1.4 to 14.2 µg Kg^−1^ and from 3.3 to 45.5 µg Kg^−1^, respectively. Both are commonly employed for repellence of the leaffolders *Cnaphalocrocis medinalis* [34], spider mites, and bugs [35]. Previously, the presence of chlorpyrifos was reported in Korean rice samples with concentrations ranging from 0.1 to 2.2 mg Kg^−1^ which exceeds the MRL [32]. Regarding bifenthrin, 10 out of 10 rice samples detected with this compound contained a content 1.1 to 4.5 times higher than the allowable MRL threshold for rice. In the fungicide group, azoxystrobin was commonly used to prevent the blast disease caused by the rice blast fungus *Pyricularia* oryzae [36], while hexaconazole is used to treat the sheath blight caused by the plant pathogenic fungus *Rhizoctonia solani* [37]. In this study, two samples contained azoxystrobin concentrations of 1.3 and 6.4 times higher than the regulated MRL. Such results were similar to the azoxystrobin concentration of 0.02 to 0.05 mg Kg^−1^ detected in rice [3], meanwhile the presence of hexaconazole was detected in Korean rice with concentrations ranging from 0.05 to 1.7 mg Kg^−1^ [32]. In the herbicide group, 2,4-D and butachlor were present in seven and ten rice samples, respectively, with concentrations within the regulated MRL range. These two compounds are used as a selective pre-emergent herbicide. Notably, 2,4-D was one of three compounds banned for rice farming in Vietnam since 2017. Specifically, banning of the 2,4-D compound was issued by Decision No. 278/QD-BNN-BVTV, 2017, banning of carbendazim was issued by Decision No. 03/QD-BNN-BVTV, 2017 and that of fipronil was by Decision No. 501/QĐ-BNN-BVTV, 2019. The presence of such prohibited compounds detected in rice samples could be due to the poor management of and lack of appropriate punishment for illegal applications of agricultural pesticides on food crops. In recent years, many studies have also reported pesticide residues in rice. In Iran, Shakouri et al. [38] reported residues of cinosulfuron, triadimenol, and tricyclazole in commercial rice samples. However, the concentrations recorded were below the MRL threshold and mainly came from imported rice. In Pakistan, Ahmad et al. [39] recorded pesticide residues, namely cyhalothrin, monocrtophos, and captan, in commercial rice. Overall, the development of a method for simultaneous analysis of 656 pesticide residues in rice has proved its efficiency aiding in a preliminary screening of rice safety before regulating commercial rice on national and international markets.

## 4. Conclusions

Overall, this study proposed a protocol for the analysis of 656 pesticides in rice. A combination of chromatography with tandem mass spectrometry, modified QuEChERS extraction method, and suitable selection of PSA+C18 solvent purification materials was successfully utilized for multi-residue analysis of pesticide in rice, resulting in 318 and 299 pesticides of high recovery range from 70 to 120% in UPLC- and GC-MS/MS analyses, respectively. All analytes displayed recovery between 70 and 120% with RSD_r_ and RSD_R_ less than 20%. Furthermore, the maximal LOQs were 10 μg Kg^−1^ in both MS methods. All of these parameters met the requirements in the SANTE/12682/2019 Guideline. Application on commercial rice samples collected in markets from Hanoi showed that 14 out of 20 samples were contaminated with at least one pesticide compound and insecticide was the most detected pesticide group in rice. In addition, 2,4-D, carbendazim, and fipronil were those compounds that were banned for rice farming in Vietnam yet detected in rice samples with concentrations within the regulated MRLs ranges.

## Figures and Tables

**Table 1:** Limits of quantification (recovery in the range of 70–120% and relative standard deviation ≤20%) at different levels.

Concentration (μg Kg^−1^)	UPLC-MS/MS		GC-MS/MS		Total	
	Number of Pesticides	% Proportion	Number of Pesticides	% Proportion	Number of Pesticides	% Proportion
1	0	0	64	20.4	64	9.8
2	302	88.6	37	11.8	339	51.8
5	32	9.4	178	56.9	210	32.1
10	7	2.1	35	10.9	41	6.3

**Table 2:** Evaluation of matrix effect in UPLC-MS/MS and GC-MS/MS.

Matrix Effect	UPLC-MS/MS		GC-MS/MS	
	Number of Pesticides	% Proportion	Number of Pesticides	% Proportion
<−50	10	2.9	1	0.3
−50 to −20	28	8.2	5	1.6
−20 to 20	301	88.3	203	64.9
20 to 50	2	0.6	36	11.5
>50	0	0.0	68	21.7

**Table 3:** Results of pesticide detection in commercial rice samples collected from markets in Hanoi, Vietnam.

No.	Compound	Group	MRL * µg Kg^−1^	Lowest Level µg Kg^−1^	Highest Level µg Kg^−1^	Number of Samples Detected	Number of Noncompliance Samples
1	2,4-D	Herbicide	100	4.3	14.5	7	0
2	Acetamiprid	Insecticide	10	1.6	43.2	11	3
3	Anthraquinone	Other	10	1.4	15.4	6	2
4	Azoxystrobin	Fungicide	10	2.3	64.2	7	2
5	Bifenthrin	Insecticide	10	3.3	45.5	12	10
6	Butachlor	Herbicide	500	5.2	149.4	10	0
7	Carbaryl	Insecticide	1000	7.0	82.4	8	0
8	Carbendazim	Fungicide	2000	2.6	100.2	11	0
9	Carbofuran	Insecticide	100	1.3	14.3	9	0
10	Carbosulfan	Insecticide	200	1.7	20.1	8	0
11	Chlorpyrifos	Insecticide	100	1.4	14.2	13	0
12	Lambda-cyhalothrin	Insecticide	10	1.7	33.4	14	3
13	Cypermethrin	Insecticide	2000	1.6	58.3	11	0
14	Dichlorvos	Insecticide	100	2.3	14.2	10	0
15	Fenobucarb	Insecticide	500	3.7	23.6	11	0
16	Fipronil	Insecticide	10	5.0	54.2	8	2
17	Hexaconazole	Fungicide	100	1.0	22.6	9	0
18	Imidacloprid	Insecticide	50	4.2	45.4	11	0
19	Indoxacarb	Insecticide	20	2.5	16.7	6	0
20	Metaflumizone	Insecticide	500	2.7	9.0	7	0
21	Permethrin, cis-	Insecticide	2000	5.2	22.5	8	0
22	Permethrin, trans-	Insecticide	2000	2.6	21.5	9	0
23	Spinosad	Insecticide	1000	3.7	14.3	7	0
24	Thiamethoxam	Insecticide	100	2.5	57.3	9	0

*: EU MRLs regulation.

## Supplementary Materials

The following are available online at https://www.mdpi.com/article/10.3390/foods10102455/s1, Table S1: List of pesticide compounds detected by UPLC- and GC-MS/MS, Table S2: List of pesticides, method parameters and results of validation parameters: linearity range, matrix effect, limit of detection, limit of quantitation, recovery and reproducibility of UPLC-MS/MS method, Table S3: PTV injection conditions; Table S4: List of pesticides, method parameters and results of validation parameters: linearity range, matrix effect, limit of detection, limit of quantitation, recovery and reproducibility of GC-MS/MS method; Figure S1: Correlation between retention time and matrix effect in (a) UPLC-MS/MS and (b) GC-MS/MS systems.
