# Supplementary material for: A Combination of Chromatography with Tandem Mass Spectrometry Systems (UPLC-MS/MS and GC-MS/MS), Modified QuEChERS Extraction and Mixed-Mode SPE Clean-Up Method for the Analysis of 656 Pesticide Residues in Rice
Source: Foods. 2021 Oct 14;10(10):2455. doi: 10.3390/foods10102455 (PMC8536010; doi:10.3390/foods10102455)
Supplement: Supplementary file 1 [file foods-10-02455-s001.zip › foods-1418983-supplementary.pdf]

foods

MDPI

*Article*

# A Combination of Chromatography with Tandem Mass Spectrometry Systems (UPLC-MS/MS and GC-MS/MS), Modified QuEChERS Extraction and Mixed-Mode SPE Clean-Up Method for the Analysis of 656 Pesticide Residues in Rice

**Thanh-Thien Tran-Lam [1], Minh Quang Bui [2], Hoa Quynh Nguyen [3], Yen Hai Dao [3,*] and Giang Truong Le [3]**

[1] Institute of Mechanics and Applied Informatics, Vietnam Academy of Science and Technology, Ho Chi Minh City 72450, Vietnam; thanhthien307@gmail.com
[2] Center for Research and Technology Transfer (CRETECH), Vietnam Academy of Science and Technology, Hanoi 10072, Vietnam; bui_quang_minh@yahoo.com
[3] Institute of Chemistry, Vietnam Academy of Science and Technology, Hanoi 10072, Vietnam; hoanq8x@gmail.com (H.Q.N.); gianglt.hsmt@vast.vn (G.T.L.)
* Correspondence: hoasinhmoitruong.vast@gmail.com; Tel.: +84-985-859-795

**Table S1.** List of pesticide compounds detected by UPLC- and GC-MS/MS.

| Number | Chemical | Catalogue/CAS Number | Company |
|---|---|---|---|
| 1 | 1-Naphthyl Acetamide | 86-86-2 | Dr Ehrenstorfer |
| 2 | 1-Naphthylacetic acid | 86-87-3 | Sigma-Aldrich |
| 3 | 1,2,3,6-Tetrahydrophthalimide,cis- | 1469-48-3 | Sigma-Aldrich |
| 4 | 2,4-D | 94-75-7 | Sigma-Aldrich |
| 5 | 2,4-DB | 94-82-6 | Sigma-Aldrich |
| 6 | 2,4'-DDD | 53-19-0 | Dr Ehrenstorfer |
| 7 | 2,4'-DDE | 3424-82-6 | Dr Ehrenstorfer |
| 8 | 2,6-Dichlorobenzamide | 2008-58-4 | Sigma-Aldrich |
| 9 | 3-Chloro-4-Methylaniline | 95-74-9 | Sigma-Aldrich |
| 10 | 3-Phenylphenol | 580-51-8 | Sigma-Aldrich |
| 11 | 3,4,5-Trimethacarb | 2686-99-9 | Dr Ehrenstorfer |
| 12 | 3,5-Dichloroaniline | 626-43-7 | Sigma-Aldrich |
| 13 | 4-chloro-o-tolyloxyacetic acid (MCPA) | 94-74-6 | Sigma-Aldrich |
| 14 | 4-Chloronitrobenzene | 100-00-5 | Sigma-Aldrich |
| 15 | 4-Chlorophenoxyacetic acid | 122-88-3 | Sigma-Aldrich |
| 16 | 4,4'-DDD | 72-54-8 | Sigma-Aldrich |
| 17 | 4,4'-Dibromobenzophenone | 3988-03-2 | Sigma-Aldrich |
| 18 | 6-Benzylaminopurine (6-BAP) | 1214-39-7 | Sigma-Aldrich |
| 19 | Acenaphthene | 83-32-9 | Sigma-Aldrich |
| 20 | Aclonifen | 74070-46-5 | Sigma-Aldrich |
| 21 | Albendazole | 54965-21-8 | Sigma-Aldrich |
| 22 | Allethrin | 584-79-2 | Sigma-Aldrich |
| 23 | Alloxydim-sodium | 55635-13-7 | Sigma-Aldrich |

| | | | |
|---|---|---|---|
| 24 | Ametoctradin | 865318-97-4 | Sigma-Aldrich |
| 25 | Amidosulfuron | 120923-37-7 | Sigma-Aldrich |
| 26 | Ancymidol | 12771-68-5 | Sigma-Aldrich |
| 27 | Anilofos | 64249-01-0 | Sigma-Aldrich |
| 28 | Asulam | 3337-71-1 | Sigma-Aldrich |
| 29 | Aramite | 140-57-8 | LGC Standard |
| 30 | Azaconazole | 60207-31-0 | Sigma-Aldrich |
| 31 | Aziprotryne | 4658-28-0 | Sigma-Aldrich |
| 32 | Benodanil | 15310-01-7 | Sigma-Aldrich |
| 33 | Bensulfuron-methyl | 83055-99-6 | Sigma-Aldrich |
| 34 | Bentazon | 25057-89-0 | Sigma-Aldrich |
| 35 | Benthiavalicarb-isopropyl | 177406-68-7 | Sigma-Aldrich |
| 36 | Benzovindiflupyr | 1072957-71-1 | Sigma-Aldrich |
| 37 | Bromobutide | 74712-19-9 | Sigma-Aldrich |
| 38 | Brompyrazon | 3042-84-0 | Dr Ehrenstorfer |
| 39 | Butachlor | 23184-66-9 | Sigma-Aldrich |
| 40 | Butralin | 33629-47-9 | Sigma-Aldrich |
| 41 | Cadusafos | 95465-99-9 | Sigma-Aldrich |
| 42 | Captan | 133-06-2 | Sigma-Aldrich |
| 43 | Chlorbromuron | 13360-45-7 | Dr Ehrenstorfer |
| 44 | Chlorbufam | 1967-16-4 | Sigma-Aldrich |
| 45 | Chlordane | 5103-74-2 | Dr Ehrenstorfer |
| 46 | Chlordecone | 143-50-0 | Dr Ehrenstorfer |
| 47 | Chlordimeform | 6164-98-3 | Sigma-Aldrich |
| 48 | Chlorethoxyfos | 54593-83-8 | Sigma-Aldrich |
| 49 | Chlorfenethol | 80-06-8 | Dr Ehrenstorfer |
| 50 | Chlorfenprop-Methyl | 14437-17-3 | Dr Ehrenstorfer |
| 51 | Chlorimuron-ethyl | 90982-32-4 | Sigma-Aldrich |
| 52 | Chloropropylate | 5836-10-2 | Dr Ehrenstorfer |
| 53 | Chlorsulfuron | 64902-72-3 | Sigma-Aldrich |
| 54 | Cinidon-Ethyl | 142891-20-1 | Sigma-Aldrich |
| 55 | Cinosulfuron | 94593-91-6 | Sigma-Aldrich |
| 56 | Clethodim | 99129-21-2 | Sigma-Aldrich |
| 57 | Clodinafop-propargyl | 105512-06- | Sigma-Aldrich |
| 58 | Clomazone | 81777-89-1 | Sigma-Aldrich |
| 59 | Clopyralid | 1702-17-6 | Sigma-Aldrich |
| 60 | Crufomate | 299-86-5 | Sigma-Aldrich |
| 61 | Cyanofenphos | 13067-93-1 | Dr Ehrenstorfer |
| 62 | Cyanophos | 2636-26-2 | Sigma-Aldrich |
| 63 | Cycloxidim | 101205-02-1 | Sigma-Aldrich |
| 64 | Cyflumetofen | 400882-07-7 | sigma-Aldrich |
| 65 | Cyprazine | 22936-86-3 | Sigma-Aldrich |
| 66 | Cyproconazole | 94361-06-5 | Sigma-Aldrich |
| 67 | Cyprofuram | 69581-33-5 | Sigma-Aldrich |
| 68 | Cyromazine | 66215-27-8 | Sigma-Aldrich |
| 69 | DEET | 134-62-3 | Sigma-Aldrich |
| 70 | Demeton-S-Sulfoxide | 2496-92-6 | Sigma-Aldrich |
| 71 | Desethylsebutylazine | 37019-18-4 | Sigma-Aldrich |
| 72 | Desethylterbuthylazine | 30125-63-4 | Sigma-Aldrich |

| | | | |
|---|---|---|---|
| 73 | Desmetryn | 1014-69-3 | Dr Ehrenstorfer |
| 74 | Dialifos | 10311-84-9 | Sigma-Aldrich |
| 75 | Dibutyl succinate | 141-03-7 | Sigma-Aldrich |
| 76 | Dicamba | 1918-00-9 | Sigma-Aldrich |
| 77 | Dicapthon | 2463-84-5 | Sigma-Aldrich |
| 78 | Dichlobenil | 1194-65-6 | Sigma-Aldrich |
| 79 | Dichlofenthion | 97-17-6 | Sigma-Aldrich |
| 80 | Dichlormid | 37764-25-3 | Sigma-Aldrich |
| 81 | Dichlorprop-methyl | 23844-57-7 | Sigma-Aldrich |
| 82 | Dichlorvos | 62-73-7 | Sigma-Aldrich |
| 83 | Diclocymet | 139920-32-4 | Sigma-Aldrich |
| 84 | Diclofop-methyl | 51338-27-3 | Sigma-Aldrich |
| 85 | Diclosulam | 145701-21-9 | Sigma-Aldrich |
| 86 | Diethatyl-ethyl | 38727-55-8 | Sigma-Aldrich |
| 87 | Diflufenican | 83164-33-4 | Sigma-Aldrich |
| 88 | Dimefuron | 34205-21-5 | Sigma-Aldrich |
| 89 | Dimethametryn | 22936-75-0 | Dr Ehrenstorfer |
| 90 | Dimethenamid | 163515-14-8 | Sigma-Aldrich |
| 91 | Dimethipin | 55290-64-7 | Dr Ehrenstorfer |
| 92 | Dimethomorph | 110488-70-5 | Sigma-Aldrich |
| 93 | Dimethyl phthalate | 131-11-3 | Sigma-Aldrich |
| 94 | Dimethylvinphos | 2274-67- | Sigma-Aldrich |
| 95 | Dimetilan | 644-64-4 | Dr Ehrenstorfer |
| 96 | Dinitramine | 29091-05-2 | Dr Ehrenstorfer |
| 97 | Dinobuton | 973-21-7 | Dr Ehrenstorfer |
| 98 | Dinoterb | 1420-07-1 | Sigma-Aldrich |
| 99 | Diofenolan | 63837-33-2 | Sigma-Aldrich |
| 100 | Dioxabenzofos | 3811-49-2 | Dr Ehrenstorfer |
| 101 | Dioxathion | 78-34-2 | Sigma-Aldrich |
| 102 | Dipropetryn | 4147-51-7 | Sigma-Aldrich |
| 103 | Disulfoton sulfone | 2497-06-5 | Sigma-Aldrich |
| 104 | Ditalimfos | 5131-24-8 | Dr Ehrenstorfer |
| 105 | Dodemorph | 1593-77-7 | Sigma-Aldrich |
| 106 | Endrin-aldehyde | 7421-93-4 | Dr Ehrenstorfer |
| 107 | Epoxiconazole | 133855-98-8 | Dr Ehrenstorfer |
| 108 | Eprinomectin | 123997-26-2 | Sigma-Aldrich |
| 109 | EPTC | 759-94-4 | Sigma-Aldrich |
| 110 | Esprocarb | 85785-20-2 | Sigma-Aldrich |
| 111 | Ethametsulfuron-methyl | 97780-06-8 | Sigma-Aldrich |
| 112 | Ethidimuron | 30043-49-3 | Dr Ehrenstorfer |
| 113 | Ethiofencarb | 53380-22-6 | Sigma-Aldrich |
| 114 | Ethiofencarb-sulfoxide | 53380-22-6 | Sigma-Aldrich |
| 115 | Ethoprophos | 13194-48-4 | Sigma-Aldrich |
| 116 | Ethoxysulfuron | 126801-58-9 | Dr Ehrenstorfer |
| 117 | Etoxazole | 153233-91-1 | Sigma-Aldrich |
| 118 | Etrimfos | 38260-54-7 | Sigma-Aldrich |
| 119 | Famphur | 52-85-7 | Sigma-Aldrich |
| 120 | Fenamiphos | 22224-92-6 | Sigma-Aldrich |
| 121 | Fenamiphos-sulfone | 31972-44-8 | Sigma-Aldrich |

| | | | |
|---|---|---|---|
| 122 | Fenbuconazole | 119446-68-3 | Dr Ehrenstorfer |
| 123 | Fenchlorphos-Oxon | 3983-45-7 | Dr Ehrenstorfer |
| 124 | Fenfuram | 24691-80-3 | Dr Ehrenstorfer |
| 125 | Fenoprop | 93-72-1 | Dr Ehrenstorfer |
| 126 | Fenothiocarb | 62850-32-2 | Sigma-Aldrich |
| 127 | Fenoxaprop-Ethyl | 66441-23-4 | Sigma-Aldrich |
| 128 | Fenoxaprop-p | 113158-40-0 | Sigma-Aldrich |
| 129 | Fenoxaprop-P-ethyl | 71283-80-2 | Sigma-Aldrich |
| 130 | Fenpiclonil | 74738-17-3 | Sigma-Aldrich |
| 131 | Fenpropidin | 67306-00- | Sigma-Aldrich |
| 132 | Fensulfothion | 115-90-2 | Sigma-Aldrich |
| 133 | Fenthion-sulfone | 3761-42-0 | Sigma-Aldrich |
| 134 | Fenthion-sulfoxide | 3761-41-9 | Sigma-Aldrich |
| 135 | Flazasulfuron | 104040-78-0 | Sigma-Aldrich |
| 136 | Fluazifop-butyl | 69806-50-4 | Sigma-Aldrich |
| 137 | Flubendazole | 31430-15-6 | Dr Ehrenstorfer |
| 138 | Fluensulfone | 318290-98-1 | Dr Ehrenstorfer |
| 139 | Flumequine | 42835-25-6 | Sigma-Aldrich |
| 140 | Flumetralin | 62924-70-3 | Sigma-Aldrich |
| 141 | Flumioxazin | 103361-09-7 | Sigma-Aldrich |
| 142 | Fluopicolide | 239110-15-7 | Dr Ehrenstorfer |
| 143 | Fluopyram | 658066-35-4 | Sigma-Aldrich |
| 144 | Fluorodifen | 15457-05-3 | Sigma-Aldrich |
| 145 | Fluoroglycofen-ethyl | 77501-90-7 | Sigma-Aldrich |
| 146 | Fluotrimazol | 31251-03-3 | Sigma-Aldrich |
| 147 | Flurochloridone | 61213-25-0 | Dr Ehrenstorfer |
| 148 | Fluroxypyr-mepthyl | 81406-37-3 | Sigma-Aldrich |
| 149 | Flurprimidol | 56425-91-3 | Sigma-Aldrich |
| 150 | Flurtamone | 96525-23-4 | Sigma-Aldrich |
| 151 | Fluthiacet-Methyl | 117337-19-6 | Sigma-Aldrich |
| 152 | Fluxapyroxad | 907204-31-3 | Sigma-Aldrich |
| 153 | Fosthiazate | 98886-44-3 | Sigma-Aldrich |
| 154 | Furmecyclox | 60568-05-0 | Dr Ehrenstorfer |
| 155 | GC multi-residue pesticide kit (Restek 32562 – 100 µg/mL each in toluene) | 32563, 32564, 32565, 32566, 32567, 32568, 32569, 32570, 32571 | Restek |
| 156 | Genite | 97-16-5 | Sigma-Aldrich |
| 157 | Haloxyfop | 69806-34-4 | Sigma-Aldrich |
| 158 | Heptachlor-exo-epoxide | 1024-57-3 | Sigma-Aldrich |
| 159 | Heptenophos | 23560-59-0 | Sigma-Aldrich |
| 160 | Imazamethabenz-methyl | 81405-85-8 | Dr Ehrenstorfer |
| 161 | Imazamox | 114311-32-9 | Sigma-Aldrich |
| 162 | Imazapic | 104098-48-8 | Sigma-Aldrich |
| 163 | Imazaquin | 81335-37-7 | Sigma-Aldrich |
| 164 | Imazosulfuron | 122548-33-8 | Sigma-Aldrich |
| 165 | Imibenconazole | 86598-92-7 | Sigma-Aldrich |
| 166 | Imidacloprid-urea | 120868-66-8 | Dr Ehrenstorfer |
| 167 | Inabenfide | 82211-24-3 | Sigma-Aldrich |
| 168 | Indanofan | 133220-30-1 | Dr Ehrenstorfer |

| | | | |
|---|---|---|---|
| 169 | Iodofenphos | 18181-70-9 | Dr Ehrenstorfer |
| 170 | Iodosulfuron-methyl | 144550-36-7 | Sigma-Aldrich |
| 171 | Iprobenfos | 26087-47-8 | Sigma-Aldrich |
| 172 | Iprovalicarb | 140923-17-7 | Sigma-Aldrich |
| 173 | Isocarbamid | 30979-48-7 | Dr Ehrenstorfer |
| 174 | Isofenphos-oxon | 31120-85-1 | Dr Ehrenstorfer |
| 175 | Isoprothiolane | 50512-35-1 | Sigma-Aldrich |
| 176 | Isopyrazam | 881685-58-1 | Sigma-Aldrich |
| 177 | Isoxaflutole | 141112-29-0 | Sigma-Aldrich |
| 178 | Isoxathion | 18854-01-8 | Sigma-Aldrich |
| 179 | Karbutilate | 4849-32-5 | Dr Ehrenstorfer |
| 180 | Kinoprene | 42588-37-4 | Sigma-Aldrich |
| 181 | Lactofen | 77501-63-4 | Sigma-Aldrich |
| 182 | LC multi-residue pesticide kit (Restek 31971 – 100 µg/mL each in acetonitrile) | 31972, 31973, 31974, 31975, 31976, 31977, 31978, 31979, 31980, 31981 | Restek |
| 183 | Lindane | 58-89-9 | Sigma-Aldrich |
| 184 | Malaoxon | 1634-78-2 | Sigma-Aldrich |
| 185 | Mecarbam | 2595-54-2 | Sigma-Aldrich |
| 186 | Mecoprop Methyl Ester | 23844-56-6 | Sigma-Aldrich |
| 187 | Mefenpyr-diethyl | 135590-91-9 | Dr Ehrenstorfer |
| 188 | Mepiquat | 24307-26-4 | Sigma-Aldrich |
| 189 | Mesosulfuron-methyl | 208465-21-8 | Sigma-Aldrich |
| 190 | Mesotrione | 104206-82-8 | Sigma-Aldrich |
| 191 | Metalaxyl-M | 70630-17-0 | Sigma-Aldrich |
| 192 | Metamitron | 41394-05-2 | Sigma-Aldrich |
| 193 | Methidathion | 950-37-8 | Sigma-Aldrich |
| 194 | Methiocarb-sulfoxide | 2635-10-1 | Sigma-Aldrich |
| 195 | Methoprene | 40596-69-8 | Sigma-Aldrich |
| 196 | Metolcarb | 1129-41-5 | Sigma-Aldrich |
| 197 | Metominostrobin | 133408-50-1 | Sigma-Aldrich |
| 198 | Metosulam | 139528-85-1 | Sigma-Aldrich |
| 199 | Metrafenone | 220899-03-6 | Dr Ehrenstorfer |
| 200 | Metronidazole | 443-48-1 | Sigma-Aldrich |
| 201 | Metsulfuronmethyl | 74223-64-6 | Sigma-Aldrich |
| 202 | Molinate | 2212-67-1 | Dr Ehrenstorfer |
| 203 | Nitrapyrin | 1929-82-4 | Sigma-Aldrich |
| 204 | Nitrothal-Isopropyl | 10552-74-6 | Dr Ehrenstorfer |
| 205 | Octachlorostyrene | 29082-74-4 | Dr Ehrenstorfer |
| 206 | Octhilinone | 26530-20-1 | Dr Ehrenstorfer |
| 207 | Ofurace | 58810-48-3 | Sigma-Aldrich |
| 208 | Oryzalin | 19044-88-3 | Sigma-Aldrich |
| 209 | Oxydemeton-methyl | 301-12-2 | Sigma-Aldrich |
| 210 | Paraoxon-methyl | 950-35-6 | Sigma-Aldrich |
| 211 | Penoxsulam | 219714-96-2 | Dr Ehrenstorfer |
| 212 | Penthiopyrad | 183675-82-3 | Dr Ehrenstorfer |
| 213 | Phorate-Sulfone | 2588-04-7 | Sigma-Aldrich |
| 214 | Phorate-Sulfoxide | 2588-03-6 | Dr Ehrenstorfer |

| | | | |
|---|---|---|---|
| 215 | Phosfolan | 947-02-4 | Dr Ehrenstorfer |
| 216 | Phosphamidon | 13171-21-6 | Sigma-Aldrich |
| 217 | Picaridin | 119515-38-7 | Dr Ehrenstorfer |
| 218 | Picloram | 1918-02-1 | Sigma-Aldrich |
| 219 | Picolinafen | 137641-05-5 | Sigma-Aldrich |
| 220 | Picoxystrobin | 117428-22-5 | Sigma-Aldrich |
| 221 | Pirimicarb-desmethyl | 30614-22-3 | Dr Ehrenstorfer |
| 222 | Probenazole | 27605-76-1 | Sigma-Aldrich |
| 223 | Propamocarb HCl | 25606-41-1 | Dr Ehrenstorfer |
| 224 | Propazine | 139-40-2 | Sigma-Aldrich |
| 225 | Propiconazole | 60207-90-1 | Sigma-Aldrich |
| 226 | Propoxycarbazone | 181274-15-7 | Sigma-Aldrich |
| 227 | Proquinazid | 189278-12-4 | Sigma-Aldrich |
| 228 | Pyflubumide | 926914-55-8 | Sigma-Aldrich |
| 229 | Pymetrozine | 123312-89-0 | Sigma-Aldrich |
| 230 | Pyridafol | 40020-01-7 | Sigma-Aldrich |
| 231 | Pyrifenox | 88283-41-4 | Sigma-Aldrich |
| 232 | Quinmerac | 90717-03-6 | Sigma-Aldrich |
| 233 | Quinoclamine | 2797-51-5 | Sigma-Aldrich |
| 234 | Quizalofop-Ethyl | 76578-14-8 | Sigma-Aldrich |
| 235 | Rabenzazole | 40341-04-6 | Dr Ehrenstorfer |
| 236 | Rimsulfuron | 122931-48-0 | Sigma-Aldrich |
| 237 | Saflufenacil | 372137-35-4 | Sigma-Aldrich |
| 238 | Sebuthylazine | 7286-69- | Dr Ehrenstorfer |
| 239 | Siduron | 1982-49-6 | Sigma-Aldrich |
| 240 | Silafluofen | 105024-66-6 | Sigma-Aldrich |
| 241 | Simeconazole | 149508-90-7 | Dr Ehrenstorfer |
| 242 | Simeton | 673-04-1 | Dr Ehrenstorfer |
| 243 | Spiroxamine | 118134-30-8 | Sigma-Aldrich |
| 244 | Tebufenozide | 112410-23-8 | Sigma-Aldrich |
| 245 | Tebupirimfos | 96182-53-5 | Sigma-Aldrich |
| 246 | Tebutam | 35256-85-0 | Dr Ehrenstorfer |
| 247 | Terbufos-Oxon-Sulfone | 56070-15-6 | Dr Ehrenstorfer |
| 248 | Terbufos-Sulfone | 56070-16-7 | Sigma-Aldrich |
| 249 | Terbuthylazine | 5915-41-3 | Sigma-Aldrich |
| 250 | tert-butyl-4-Hydroxyanisole | 88-32-4 | Sigma-Aldrich |
| 251 | Tetrasul | 2227-13-6 | Sigma-Aldrich |
| 252 | Thenylchlor | 96491-05-3 | Dr Ehrenstorfer |
| 253 | Thiazafluron | 25366-23-8 | Sigma-Aldrich |
| 254 | Thiazopyr | 117718-60-2 | Sigma-Aldrich |
| 255 | Thiocyclam | 31895-22-4 | Sigma-Aldrich |
| 256 | Thiodicarb | 59669-26-0 | Sigma-Aldrich |
| 257 | Thiofanox-Sulfone | 39184-59-3 | Dr Ehrenstorfer |
| 258 | Thiofanox-Sulfoxide | 39184-27-5 | Sigma-Aldrich |
| 259 | Thionazin | 297-97-2 | Sigma-Aldrich |
| 260 | Thiram | 137-26-8 | Sigma-Aldrich |
| 261 | Tiocarbazil | 36756-79-3 | Dr Ehrenstorfer |
| 262 | Tolprocarb | 911499-62-2 | HPC Standards GmbH |

| | | | |
|---|---|---|---|
| 263 | Tolylfluanid | 731-27-1 | Sigma-Aldrich |
| 264 | Tolylsulfonylbutylurea | 64-77-7 | Sigma-Aldrich |
| 265 | Tralkoxydim | 87820-88-0 | Sigma-Aldrich |
| 266 | Triasulfuron | 82097-50-5 | Sigma-Aldrich |
| 267 | Tribenuron-methyl | 101200-48-0 | Sigma-Aldrich |
| 268 | Tridiphane | 58138-08-2 | Sigma-Aldrich |
| 269 | Trietazine | 13171-25-0 | Sigma-Aldrich |
| 270 | Trifenmorph | 1420-06-0 | Sigma-Aldrich |
| 271 | Triphenyl phosphate | 115-86-6 | Sigma-Aldrich |
| 272 | Uniconazole | 83657-17-4 | Dr Ehrenstorfer |
| 273 | Valifenalate | 283159-90-0 | Dr Ehrenstorfer |
| 274 | Vamidothion sulfoxide | 20300-00-9 | Dr Ehrenstorfer |

**Table S2.** List of pesticides, method parameters and results of validation parameters: linearity range, matrix effect, limit of detection, limit of quantitation, recovery and reproducibility of UPLC-MS/MS method.

| | Compound | RT (min) | ESI mode | RF lens (V) | Quantitative peak (collision energy) | Confirm peak (collision energy) | Linear range | $R^2$ | % Recovery (% $RSD_R$, n = 12) | | | LOD (µg/kg) | LOQ (µg/kg) | Matrix effect (%) |
|---|---|---|---|---|---|---|---|---|---|---|---|---|---|---|
| | | | | | | | | | 10 µg/kg | 50 µg/kg | 100 µg/kg | | | |
| 1 | Mepiquat | 0.81 | + | 130 | 114.1 > 58.1 (32) | 114.1 > 98.1 (15) | 1-200 | 0.9981 | 89.1 (6.2) | 89.4 (4.3) | 87.6 (3.7) | 0.6 | 2 | 13.3 |
| 2 | Acephate | 1.81 | + | 75 | 184.0 > 143.0 (9) | 184.0 > 95.17 (21) | 1-200 | 0.9988 | 91.4 (6.6) | 90.1 (5.2) | 86.9 (5.6) | 0.6 | 2 | −12.7 |
| 3 | Formetanate hydrochloride | 2.10 | + | 111 | 222.1 > 164.9 (16) | 222.1 > 120.1 (26) | 1-200 | 0.9981 | 90.4 (5.6) | 92.0 (5.6) | 91.0 (5.9) | 0.6 | 2 | 14.5 |
| 4 | Aminocarb | 2.12 | + | 105 | 209.1 > 137.1 (23) | 209.1 > 152.1 (15) | 1-200 | 0.9992 | 87.0 (5.2) | 88.2 (5.6) | 88.6 (5.8) | 0.6 | 2 | -51.7 |
| 5 | Aldicarb sulfoxide | 2.22 | + | 59 | 207.1 > 132.0 (8) | 207.1 > 89.15 (10) | 1-200 | 0.999 | 87.4 (4.4) | 90.3 (5.1) | 87.1 (4.3) | 0.6 | 2 | −10.7 |
| 6 | Dinotefuran | 2.25 | + | 79 | 203.1 > 129.1 (12) | 203.1 > 114.1 (8) | 1-200 | 0.9991 | 91.8 (3.2) | 89 (5.3) | 92.1 (5.6) | 0.6 | 2 | -16.4 |
| 7 | Butoxycarboxim | 2.32 | + | 75 | 223.0 > 166.1 (15) | 223.0 > 106.1 (17) | 1-200 | 0.9984 | 88.5 (5.5) | 88.9 (3.7) | 88.8 (4.4) | 0.6 | 2 | −29.4 |
| 8 | Methamidophos | 2.45 | + | 80 | 142.1 > 94.17 (16) | 142.1 > 125.0 (16) | 1-200 | 0.9995 | 89.1 (5.2) | 87.2 (5.7) | 91.9 (5.2) | 0.6 | 2 | −9.97 |
| 9 | Vamidothion sulfoxide | 2.54 | + | 102 | 79.1 >64.1 (31) | 79.1 >61.1 (17) | 1-200 | 0.9986 | 87.5 (5.7) | 91.8 (3.1) | 88.9 (5.9) | 0.6 | 2 | 13.4 |
| 10 | Flonicamid | 2.60 | + | 123 | 230.0 >203.2 (15) | 230.0 >174.1 (16) | 1-200 | 0.9985 | 91.6 (7) | 88 (7) | 87.7 (4.2) | 0.6 | 2 | −23.2 |
| 11 | Picloram | 2.62 | − | 91 | 238.9 >122.9 (23) | 238.9 >80.1 (29) | 5-200 | 0.9991 | 91 (6.5) | 88.6 (6.7) | 89.4 (5.6) | 3 | 10 | 15.5 |
| 12 | Aldicarb-sulfone | 2.66 | + | 89 | 182.1 >136.1 (39) | 182.1 >56.1 (14) | 1-200 | 0.999 | 89.4 (5.7) | 88.3 (5.6) | 91.8 (5.1) | 0.6 | 2 | −17.1 |
| 13 | Thiamethoxam | 2.67 | + | 104 | 292.1 >211.1 (13) | 292.1 >181.1 (23) | 1-200 | 0.9994 | 88.7 (5.4) | 89.1 (5.3) | 92.6 (4.3) | 0.6 | 2 | −25.8 |
| 14 | Asulam | 2.77 | + | 131 | 231.1 >156.1 (41) | 231.1 >108.1 (34) | 2-200 | 0.9987 | 88.7 (5) | 89.7 (3.8) | 90.7 (5.6) | 1.5 | 5 | 15.4 |
| 15 | Cyromazine | 2.95 | + | 110 | 167.1 >85.0 (19) | 167.1 >125.0 (18) | 1-200 | 0.9982 | 89 (5.9) | 91.1 (5.4) | 89 (5.6) | 0.6 | 2 | 19.3 |
| 16 | Mexacarbate | 3.05 | + | 117 | 223.1 >151.1 (24) | 223.1 >166.1 (16) | 1-200 | 0.9988 | 87.5 (5.7) | 87.6 (5.1) | 89 (5.2) | 0.6 | 2 | −17.9 |
| 17 | Trichlorfon | 3.14 | + | 112 | 256.9 >109.1 (21) | 256.9 >79.26 (32) | 1-200 | 0.9998 | 89.5 (6) | 91.2 (6) | 91.8 (4.1) | 0.6 | 2 | -10.7 |
| 18 | Vamidothion | 3.14 | + | 94 | 288.1 >146.1 (15) | 288.1 >118.1 (25) | 1-200 | 0.9983 | 88.4 (5.5) | 87.1 (5.4) | 91.4 (4.1) | 0.6 | 2 | -12.7 |
| 19 | Carbofuran-3-hydroxy | 3.17 | + | 121 | 238.2 >181.1 (12) | 238.2 >163.1 (18) | 1-200 | 0.9982 | 90.4 (4.2) | 88.9 (6.4) | 86.9 (5.8) | 0.6 | 2 | −15.8 |
| 20 | Fenuron | 3.17 | + | 40 | 165.2 >72.31 (15) | 165.2 >77.28 (30) | 1-200 | 0.9998 | 90.5 (6) | 89.2 (5.5) | 91.6 (4.1) | 0.6 | 2 | -17.6 |
| 21 | Dioxacarb | 3.19 | + | 91 | 224.1 >123.1 (19) | 224.1 >95.24 (29) | 1-200 | 0.9987 | 88.2 (5.4) | 91.4 (7) | 87.7 (6.3) | 0.6 | 2 | -15.5 |

| | | | | | | | | | | | | | | |
|---|---|---|---|---|---|---|---|---|---|---|---|---|---|---|
| 22 | Fuberidazole | 3.20 | + | 138 | 185.1 >157.1 (23) | 185.1 > 65.4 (48) | 1-200 | 0.9998 | 88.8 (6.7) | 87.6 (4.7) | 90 (6.6) | 0.6 | 2 | 8.1 |
| 23 | Dimethoate | 3.24 | + | 83 | 230.6 >125.0 (24) | 230.6 >79.24 (33) | 1-200 | 0.9978 | 89.8 (6.5) | 91.6 (6.6) | 88.6 (5.6) | 0.6 | 2 | −19.8 |
| 24 | Ethiofencarb-sulfoxide | 3.28 | + | 99 | 242.1 >185.1 (42) | 242.1 >107.1 (11) | 2-200 | 0.9998 | 91.7 (5.7) | 87.9 (4.3) | 87.3 (5.1) | 1.5 | 5 | 17.6 |
| 25 | Pirimicarb-desmethyl | 3.31 | + | 146 | 225.1 >72.1 (37) | 225.1 >168.1 (27) | 1-200 | 0.9986 | 89.7 (5.9) | 86.4 (4.8) | 89.1 (6.2) | 0.6 | 2 | -11.5 |
| 26 | Dicrotophos | 3.33 | + | 106 | 238.0 >112.2 (15) | 238.0 >193.0 (10) | 1-200 | 0.9989 | 91.4 (5.4) | 89.7 (5.4) | 93.3 (4.7) | 0.6 | 2 | -23.6 |
| 27 | Thiofanox-Sulfoxide | 3.36 | + | 115 | 235.1 >104.1 (28) | 235.1 >76.1 (12) | 1-200 | 0.9988 | 89 (6.4) | 90.3 (5.2) | 90.1 (5.5) | 0.6 | 2 | -16.4 |
| 28 | Imidacloprid-urea | 3.37 | + | 161 | 212.1 >128.1 (23) | 212.1 >99.1 (12) | 1-200 | 0.9992 | 91.3 (5.8) | 88.8 (4.4) | 90.1 (6.2) | 0.6 | 2 | 12.2 |
| 29 | Methiocarb-sulfoxide | 3.51 | + | 159 | 258.1 >122.1 (39) | 258.1 >201.1 (24) | 1-200 | 0.9988 | 89.6 (5.8) | 90.3 (6) | 89.5 (6.3) | 0.6 | 2 | 14.7 |
| 30 | Pymetrozine | 3.52 | + | 113 | 218.0 >78.0 (38) | 218.0 >105.0 (20) | 2-200 | 0.9994 | 90.7 (5.6) | 89.8 (4.8) | 89.9 (5.4) | 1.5 | 5 | 16.3 |
| 31 | Chlordimeform | 3.54 | + | 135 | 197.1 >125.1 (20) | 197.1 >152.1 (15) | 1-200 | 0.9993 | 86.8 (5) | 87.2 (5) | 89.5 (5.1) | 0.6 | 2 | -10.4 |
| 32 | Omethoate | 3.56 | + | 101 | 214.1 > 182.9 (13) | 214.1 >125.1 (17) | 1-200 | 0.9983 | 91.7 (5.9) | 90.7 (3.7) | 91.3 (5.9) | 0.6 | 2 | -10.4 |
| 33 | Metamitron | 3.57 | + | 90 | 203.1 > 175.1 (33) | 203.1 >104.1 (14) | 1-200 | 0.9994 | 91.4 (4) | 89.4 (5.8) | 89.4 (5.8) | 0.6 | 2 | 12.3 |
| 34 | Metronidazole | 3.59 | + | 119 | 172.1 > 54.9 (29) | 172.1 >98.9 (14) | 1-200 | 0.9991 | 90.1 (6.1) | 88.3 (6.8) | 90.7 (5) | 0.6 | 2 | 14.7 |
| 35 | Demeton-S-Sulfoxide | 3.65 | + | 101 | 263.1 >140.9 (40) | 263.1 >234.9 (28) | 1-200 | 0.9984 | 87.7 (4.9) | 84.7 (3.3) | 89.5 (4.4) | 0.6 | 2 | -13.4 |
| 36 | Propamocarb HCl | 3.65 | + | 96 | 190.2 >102.1 (17) | 190.2 >145.1 (13) | 1-200 | 0.9982 | 89.5 (5.1) | 90 (4.2) | 90.1 (6.5) | 0.6 | 2 | 12.2 |
| 37 | Quinmerac | 3.66 | + | 142 | 222.1 >204.1 (20) | 222.1 >141.2 (34) | 1-200 | 0.9996 | 88.3 (5.9) | 89.9 (5.4) | 89.3 (5.4) | 0.6 | 2 | 10.4 |
| 38 | Ethidimuron | 3.68 | + | 133 | 265.1 >161.9 (40) | 265.1 >208.1 (13) | 1-200 | 0.9985 | 89.1 (6.5) | 91 (5.9) | 88.9 (6.6) | 0.6 | 2 | 11.7 |
| 39 | Imazamox | 3.68 | + | 86 | 305.1 >193.1 (25) | 305.1 >261.1 (24) | 2-200 | 0.9988 | 89.6 (5.5) | 89.8 (4.7) | 90.2 (6.7) | 1.5 | 5 | -13.4 |
| 40 | Isocarbamid | 3.68 | + | 90 | 186.1 >87.1 (21) | 186.1 >130.1 (30) | 2-200 | 0.9986 | 88.9 (6.7) | 87 (5.6) | 89.4 (5.5) | 1.5 | 5 | -7.7 |
| 41 | Simeton | 3.69 | + | 121 | 198.1 >114.1 (30) | 198.1 >128.1 (14) | 1-200 | 0.9994 | 92.2 (4.1) | 91.8 (5.1) | 91.3 (6.8) | 0.6 | 2 | 11 |
| 42 | Imazapic | 3.85 | + | 143 | 276.1 >86.1 (23) | 276.1 >231.1 (18) | 1-200 | 0.998 | 92 (5.2) | 86 (5.1) | 90.5 (7) | 0.6 | 2 | -6.4 |
| 43 | Oxamyl | 3.85 | + | 95 | 242.1 >72.1 (19) | 242.1 >120.9 (12) | 1-200 | 0.9996 | 91.1 (5.5) | 89 (5) | 90.1 (4.7) | 0.6 | 2 | -10.6 |
| 44 | Butocarboxim | 3.87 | + | 46 | 213.0 >75.05 (16) | 213.0 >155.9 (10) | 1-200 | 0.9985 | 87.4 (4.1) | 89.1 (6.3) | 91.2 (5.8) | 0.6 | 2 | 10.7 |
| 45 | Brompyrazon | 3.89 | + | 87 | 266.1 >173.1 (34) | 266.1 >91.2 (27) | 1-200 | 0.9988 | 90.7 (5.9) | 91.8 (5.4) | 91 (5.6) | 0.6 | 2 | 14.5 |
| 46 | Nitenpyram | 3.91 | + | 120 | 271.1 >237.0 (21) | 271.1 >224.0 (18) | 1-200 | 0.999 | 85.1 (2.5) | 79.3 (4.9) | 79.3 (6.6) | 0.6 | 2 | -21.4 |
| 47 | Dimetilan | 3.94 | + | 163 | 241.1 >196.1 (42) | 241.1 >214.1 (14) | 1-200 | 0.9994 | 90.2 (6.3) | 88.6 (6.8) | 91.3 (4.8) | 0.6 | 2 | 10.3 |
| 48 | Ethirimol | 3.96 | + | 165 | 210.2 >140.1 (23) | 210.2 >98.2 (28) | 1-200 | 0.9992 | 75 (2.6) | 74.4 (4.3) | 72 (3.3) | 0.6 | 2 | −13.1 |
| 49 | Aldicarb | 3.97 | + | 58 | 208.1 >116.1 (10) | 208.1 >89.15 (10) | 1-200 | 0.9992 | 89.7 (4.9) | 88.5 (6.9) | 88.1 (5.8) | 0.6 | 2 | −12.7 |
| 50 | Oxydemeton-methyl | 3.97 | + | 139 | 235.1 >76.1 (30) | 235.1 >104.1 (17) | 1-200 | 0.9987 | 90.7 (5.2) | 89 (4.4) | 91.9 (5.9) | 0.6 | 2 | 10.4 |
| 51 | Thiofanox-Sulfone | 3.97 | + | 92 | 207.1 >95.1 (30) | 207.1 >179.1 (22) | 1-200 | 0.9991 | 92 (3.5) | 89.4 (4.1) | 89.6 (6) | 0.6 | 2 | 10.5 |
| 52 | Pyridafol | 4.03 | + | 132 | 192.1 >160.1 (20) | 192.1 >105.2 (32) | 1-200 | 0.9998 | 88.6 (5.8) | 90.2 (4.6) | 88.7 (6.2) | 0.6 | 2 | -12.3 |
| 53 | Carbendazim | 4.15 | + | 59 | 163.1 >88.3 (10) | 163.1 >73.3 (28) | 1-200 | 0.9987 | 91.3 (5.8) | 89.5 (6.5) | 91.5 (4.7) | 0.6 | 2 | −53.4 |
| 54 | Methomyl | 4.29 | + | 89 | 237.1 >192.1 (9) | 237.1 >120.1 (17) | 1-200 | 0.9983 | 89.6 (5.9) | 92 (6.3) | 89.1 (5.8) | 0.6 | 2 | −15.3 |
| 55 | Carbetamide | 4.44 | + | 109 | 256.1 >209.1 (16) | 256.1 >175.1 (18) | 1-200 | 0.9996 | 90.9 (6.2) | 89.9 (6.1) | 90.3 (4.5) | 0.6 | 2 | −2.18 |
| 56 | Imidacloprid | 4.47 | + | 97 | 279.1 >132.1 (31) | 279.1 >219.1 (12) | 1-200 | 0.9998 | 90 (6.2) | 87.6 (4.7) | 89.9 (5.6) | 0.6 | 2 | −1.48 |

| | | | | | | | | | | | | | | |
|---|---|---|---|---|---|---|---|---|---|---|---|---|---|---|
| 57 | Oxadixyl | 4.47 | + | 178 | 202.1 >175.0 (28) | 202.1 >131.1 (36) | 1-200 | 0.9997 | 86.1 (5) | 88.1 (4.3) | 89.4 (6.6) | 0.6 | 2 | −17.9 |
| 58 | Thiabendazole | 4.47 | + | 117 | 223.1 >126.0 (20) | 223.1 >90.1 (35) | 1-200 | 0.9996 | 90.5 (5.9) | 89.9 (5.2) | 90.1 (4.6) | 1.5 | 5 | −2.27 |
| 59 | Acetamiprid | 4.80 | + | 88 | 224.1 >193.0 (9) | 224.1 >127.1 (10) | 1-200 | 0.9989 | 91.2 (6) | 89.5 (5.4) | 90.6 (5.6) | 0.6 | 2 | −10.2 |
| 60 | Monocrotophos | 4.80 | + | 83 | 210.1 >111.1 (17) | 210.1 >93.21 (27) | 1-200 | 0.9992 | 90.8 (5.6) | 92 (4.5) | 91.8 (5.7) | 0.6 | 2 | −79.7 |
| 61 | Propoxur | 4.84 | + | 94 | 224.1 >167.1 (10) | 224.1 >109.1 (21) | 1-200 | 0.9995 | 89 (6) | 91.2 (5.3) | 90.2 (5.8) | 0.6 | 2 | −8.33 |
| 62 | Bendiocarb | 4.97 | + | 127 | 229.1 >172.1 (20) | 229.1 >116.1 (30) | 1-200 | 0.9988 | 88.4 (5.3) | 91.7 (3.7) | 90 (6.3) | 0.6 | 2 | −11.1 |
| 63 | Tebuthiuron | 5.13 | + | 46 | 265.1 >202.1 (29) | 265.1 >127.2 (13) | 1-200 | 0.9991 | 89.8 (5.2) | 86.5 (4.7) | 91.7 (4.5) | 0.6 | 2 | −13.00 |
| 64 | Paraoxon-methyl | 5.14 | + | 94 | 166.1 >94.1 (40) | 166.1 >109.1 (23) | 2-200 | 0.9998 | 87.3 (5.5) | 87.6 (4.8) | 90.2 (6.4) | 1.5 | 5 | 5.4 |
| 65 | Metolcarb | 5.15 | + | 169 | 214.0 >68.35 (36) | 214.0 >96.2 (27) | 1-200 | 0.9987 | 91.9 (5.9) | 91.2 (5.8) | 92.9 (5.9) | 0.6 | 2 | 16.4 |
| 66 | Simetryn | 5.16 | + | 119 | 241.1 >91.1 (35) | 241.1 >184.1 (27) | 1-200 | 0.9984 | 89.4 (5.6) | 92.5 (4.9) | 91.4 (5.2) | 0.6 | 2 | -11.7 |
| 67 | Thiazafluron | 5.21 | + | 169 | 340.2 >244.0 (20) | 340.2 >320.1 (17) | 1-200 | 0.9982 | 88.8 (4.4) | 92.8 (4.2) | 86.5 (5.3) | 0.6 | 2 | 10.2 |
| 68 | Mesotrione | 5.22 | + | 61 | 218.1 >125.1 (15) | 218.1 >97.12 (24) | 2-200 | 0.9989 | 85.8 (4) | 90 (6.3) | 86.7 (5.1) | 1.5 | 5 | 16.4 |
| 69 | Pyracarbolid | 5.23 | + | 50 | 208.1 >137.1 (40) | 208.1 >180.1 (28) | 1-200 | 0.9993 | 102.1 (4.3) | 101.4 (5.3) | 101.2 (3.9) | 0.6 | 2 | −13.6 |
| 70 | Quinoclamine | 5.24 | + | 103 | 312.1 >267.1 (31) | 312.1 >284.1 (29) | 2-200 | 0.9985 | 90.1 (6.1) | 92.5 (4.1) | 86.9 (6.3) | 1.5 | 5 | 17.4 |
| 71 | Imazaquin | 5.25 | + | 127 | 253.1 >126.1 (22) | 253.1 > 90.2 (41) | 1-200 | 0.9996 | 91.6 (3.2) | 89.4 (4.2) | 87.6 (5.1) | 0.6 | 2 | 16.9 |
| 72 | Thiacloprid | 5.25 | + | 139 | 289.1 >114.9 (31) | 289.1 >171.1 (31) | 1-200 | 0.999 | 89.4 (6.3) | 86.1 (4.4) | 87.4 (4.9) | 0.6 | 2 | -14.6 |
| 73 | Terbufos-Oxon-Sulfone | 5.29 | + | 94 | 257.1 >135.2 (35) | 257.1 >185.1 (28) | 2-200 | 0.9987 | 90.3 (6.2) | 88.9 (4.8) | 91.6 (5.1) | 1.5 | 5 | 17.4 |
| 74 | Ancymidol | 5.32 | + | 225 | 384.9 >306.9 (25) | 384.9 >198.9 (40) | 1-200 | 0.9998 | 89.5 (5.6) | 91.4 (6.1) | 91.5 (5.9) | 0.6 | 2 | 16.4 |
| 75 | Sulfentrazone | 5.38 | − | 150 | 190.1 >163.1 (24) | 190.1 >136.1 (29) | 1-200 | 0.9992 | 89 (3.2) | 89.8 (7.4) | 87.7 (5.7) | 0.6 | 2 | 11 |
| 76 | Tricyclazole | 5.52 | + | 63 | 382.1 >167.1 (29) | 382.1 >314.1 (17) | 1-200 | 0.9995 | 90.3 (5.8) | 89.4 (6.1) | 89.4 (4.7) | 0.6 | 2 | -13 |
| 77 | Carbaryl | 5.67 | + | 114 | 280.1 >136.1 (21) | 280.1 >224.1 (20) | 1-200 | 0.9998 | 91.7 (6.1) | 89 (6.6) | 88.6 (4.6) | 0.6 | 2 | -15.5 |
| 78 | Karbutilate | 5.69 | + | 157 | 226.2 >170.1 (21) | 226.2 >142.1 (26) | 1-200 | 0.9992 | 90.1 (5.3) | 88 (6.1) | 88 (4.6) | 0.6 | 2 | 16.6 |
| 79 | Secbumeton | 5.71 | + | 163 | 226.2 >142.1 (23) | 226.2 >184.1 (19) | 1-200 | 0.9996 | 89.3 (5.6) | 90.1 (4.4) | 85.5 (4.6) | 0.6 | 2 | -14.8 |
| 80 | Prometon | 5.72 | + | 108 | 336.1 >266.1 (22) | 336.1 >308.1 (19) | 1-200 | 0.9998 | 91 (6.4) | 90.6 (7.1) | 92.2 (5.8) | 0.6 | 2 | -15 |
| 81 | Fenamiphos-sulfone | 5.74 | + | 125 | 262.1 >234.1 (21) | 262.1 >238.1 (19) | 2-200 | 0.9994 | 87.9 (4.6) | 87.4 (5.1) | 87.7 (5) | 1.5 | 5 | 12 |
| 82 | Flumequine | 5.74 | + | 136 | 226.2 >65.0 (47) | 226.2 >91.0 (25) | 1-200 | 0.9986 | 90.2 (5.1) | 90.2 (5.9) | 87 (4.6) | 0.6 | 2 | 14.7 |
| 83 | 6-BAP | 5.76 | + | 159 | 315.1 >127.2 (39) | 315.1 >99.1 (12) | 1-200 | 0.9994 | 88.9 (5.6) | 87.3 (6) | 87.3 (5.6) | 0.6 | 2 | 13.4 |
| 84 | Malaoxon | 5.87 | + | 105 | 399.1 >199.1 (25) | 399.1 >367.1 (11) | 1-200 | 0.9993 | 90.6 (5) | 87.8 (5.9) | 91.6 (5.5) | 0.6 | 2 | 16.6 |
| 85 | Propoxycarbazone | 5.88 | + | 140 | 226.1 >170.1 (19) | 226.1 >142.1 (25) | 1-200 | 0.9987 | 88.5 (4.3) | 88.4 (5) | 85.1 (2.1) | 0.6 | 2 | -17.4 |
| 86 | Terbumeton | 5.89 | + | 157 | 355.1 >73.1 (28) | 355.1 >108.1 (26) | 1-200 | 0.9999 | 88.9 (4.3) | 87.1 (5.3) | 91.6 (6.2) | 0.6 | 2 | -11.5 |
| 87 | Thiodicarb | 5.89 | + | 46 | 241.1 >184.1 (5) | 241.1 >61.1 (13) | 2-200 | 0.9982 | 86.5 (5) | 90 (5.3) | 92.1 (4.4) | 1.5 | 5 | 16.7 |
| 88 | Thiofanox | 5.97 | + | 94 | 215.1 >126.1 (20) | 215.1 >99.16 (36) | 1-200 | 0.9986 | 90.2 (4.8) | 89.7 (6.5) | 88.4 (6.6) | 1.5 | 5 | 5.9 |
| 89 | Monolinuron | 5.98 | + | 118 | 358.1 >167.1 (41) | 358.1 >290.1 (29) | 1-200 | 0.9994 | 90.5 (5.7) | 87.8 (5.2) | 91.1 (5.8) | 0.6 | 2 | −9.09 |
| 90 | Chlorsulfuron | 6.11 | + | 119 | 295.1 >232.1 (42) | 295.1 >280.1 (35) | 2-200 | 0.9995 | 87.1 (5.7) | 88 (5.1) | 87.4 (5.2) | 1.5 | 5 | 17.9 |

| | | | | | | | | | | | | | | |
|---|---|---|---|---|---|---|---|---|---|---|---|---|---|---|
| 91 | Fenthion-sulfoxide | 6.15 | + | 126 | 233.1 >72.32 (22) | 233.1 >160.0 (30) | 1-200 | 0.9998 | 89.9 (4.9) | 91 (5.3) | 87.6 (5) | 0.6 | 2 | -12.8 |
| 92 | Fluometuron | 6.16 | + | 142 | 402.1 >137.1 (35) | 402.1 >237.1 (10) | 1-200 | 0.9996 | 89.4 (7) | 88.7 (5.8) | 89.9 (5.7) | 0.6 | 2 | −6.08 |
| 93 | Triasulfuron | 6.16 | + | 134 | 368.1 >259.1 (44) | 368.1 >109.0 (14) | 1-200 | 0.9996 | 88.2 (4.6) | 89 (4.4) | 89.5 (4.1) | 0.6 | 2 | 16.4 |
| 94 | Amidosulfuron | 6.17 | + | 123 | 300.1 >127.0 (22) | 300.1 >174.2 (14) | 1-200 | 0.9981 | 92.3 (5.5) | 87.6 (6.1) | 89.6 (5.7) | 0.6 | 2 | 18.7 |
| 95 | Phosphamidon | 6.20 | + | 136 | 432.1 >156.1 (29) | 432.1 >325.1 (32) | 1-200 | 0.9983 | 91.7 (6.1) | 88.9 (6.2) | 90.8 (3.3) | 0.6 | 2 | -15.4 |
| 96 | Rimsulfuron | 6.23 | + | 153 | 414.0 >157.1 (22) | 414.0 >183.1 (16) | 2-200 | 0.9998 | 91.9 (4.7) | 87.3 (6.5) | 86.8 (4.2) | 1.5 | 5 | -6.8 |
| 97 | Cinosulfuron | 6.25 | + | 123 | 239.2 >182.1 (18) | 239.2 >72.30 (23) | 1-200 | 0.9993 | 88.8 (5.6) | 91 (4.5) | 88.6 (5.9) | 0.6 | 2 | 5.2 |
| 98 | Pirimicarb | 6.29 | + | 85 | 226.1 >107.1 (18) | 226.1 >77.0 (45) | 1-200 | 0.9996 | 89.2 (5.3) | 90 (6.8) | 89.9 (5.9) | 0.6 | 2 | -13.6 |
| 99 | Probenazole | 6.32 | + | 65 | 224.0 >39.2 (40) | 224.0 >41.1 (14) | 2-200 | 0.9994 | 88.2 (5.4) | 87.5 (4) | 86.3 (3.4) | 1.5 | 5 | -7.5 |
| 100 | Albendazole | 6.37 | + | 121 | 266.1 >192.1 (39) | 266.1 >234.1 (15) | 1-200 | 0.9998 | 89.3 (5.4) | 89.4 (4.8) | 89.8 (6.7) | 0.6 | 2 | 11.5 |
| 101 | Phorate-Sulfoxide | 6.44 | + | 102 | 276.1 >171.1 (20) | 276.1 >199.1 (27) | 1-200 | 0.9996 | 88.7 (5.4) | 87.3 (4.8) | 90.6 (6.1) | 0.6 | 2 | 15.2 |
| 102 | Propham | 6.45 | + | 25 | 180.1 >138.0 (8) | 180.1 >120.1 (19) | 1-200 | 0.9981 | 92.6 (5.3) | 89.9 (5) | 92.6 (4.9) | 1.5 | 5 | −7.43 |
| 103 | Isoprocarb | 6.49 | + | 89 | 194.1 >95.22 (18) | 194.1 >137.1 (10) | 1-200 | 0.9997 | 91.3 (5.5) | 90.9 (5.4) | 88.7 (5.2) | 0.6 | 2 | −9.09 |
| 104 | Cyprazine | 6.52 | + | 104 | 228.1 >104.1 (34) | 228.1 >186.1 (21) | 1-200 | 0.9983 | 89 (5.4) | 90.5 (6.5) | 91.7 (5.6) | 0.6 | 2 | 14.9 |
| 105 | Ethametsulfuron-methyl | 6.53 | + | 125 | 411.1 >168.1 (21) | 411.1 >196.1 (20) | 1-200 | 0.9988 | 87.1 (5.1) | 89.5 (5.8) | 88.8 (6.1) | 0.6 | 2 | 12.2 |
| 106 | Ametryn | 6.66 | + | 173 | 228.1 >186.1 (20) | 228.1 >138.1 (23) | 1-200 | 0.9999 | 87.8 (5.9) | 88.1 (5.4) | 90 (5.8) | 0.6 | 2 | −6.08 |
| 107 | Metribuzin | 6.77 | + | 151 | 215.1 >187.1 (20) | 215.1 >171.1 (24) | 1-200 | 0.9985 | 89.3 (5.6) | 86.6 (6.2) | 89.9 (5.3) | 0.6 | 2 | −12.7 |
| 108 | Fenfuram | 6.85 | + | 119 | 202.1 >92.1 (26) | 202.1 >120.1 (24) | 1-200 | 0.9982 | 89 (4.9) | 92.1 (6.1) | 89.3 (7) | 0.6 | 2 | 17.4 |
| 109 | Methoprotryne | 6.85 | + | 171 | 272.2 >198.1 (23) | 272.2 >240.1 (20) | 1-200 | 0.9999 | 91 (6.8) | 90.5 (5.2) | 90.4 (6.6) | 0.6 | 2 | -9.6 |
| 110 | Mesosulfuron-methyl | 6.86 | + | 149 | 504.1 >156.1 (35) | 504.1 >306.1 (18) | 1-200 | 0.9994 | 90.3 (4.9) | 90.7 (6.3) | 91.3 (5.4) | 0.6 | 2 | -9.5 |
| 111 | Metosulam | 6.86 | + | 9 | 418.0 >175.0 (20) | 418.0 >354.1 (24) | 1-200 | 0.9994 | 92.1 (5.2) | 86.8 (6.2) | 90.2 (5.2) | 0.6 | 2 | 10.7 |
| 112 | Simazine | 6.86 | + | 38 | 202.0 >132.0 (20) | 202.0 >104.0 (26) | 1-200 | 0.9961 | 89.6 (5.8) | 90.4 (5.1) | 90.6 (4.6) | 0.6 | 2 | -20.3 |
| 113 | Metsulfuron methyl | 6.87 | + | 171 | 382.1 >167.0 (16) | 382.1 >198.9 (22) | 1-200 | 0.9992 | 92.3 (5.6) | 86.6 (6.6) | 89.1 (6.3) | 0.6 | 2 | 18.8 |
| 114 | Picaridin | 6.87 | + | 113 | 230.2 >130.1 (27) | 230.2 >174.1 (16) | 1-200 | 0.9997 | 90.4 (5.6) | 87.8 (5.4) | 89.1 (5.5) | 0.6 | 2 | 10.4 |
| 115 | Azaconazole | 6.91 | + | 115 | 300.0 >158.9 (32) | 300.0 >231.0 (33) | 2-200 | 0.9986 | 86.8 (6.2) | 87.9 (5.7) | 90.2 (4.2) | 1.5 | 5 | 16.8 |
| 116 | Carbofuran | 6.98 | + | 100 | 222.1 >165.1 (15) | 222.1 >123.1 (25) | 1-200 | 0.9991 | 89.9 (6.6) | 91.6 (5) | 89.7 (5.1) | 0.6 | 2 | -3.1 |
| 117 | Ofurace | 7.02 | + | 117 | 282.1 >160.2 (24) | 282.1 >254.1 (12) | 1-200 | 0.9987 | 90.2 (4.6) | 88 (2.7) | 89.6 (5.9) | 0.6 | 2 | 6.5 |
| 118 | Bentazon | 7.06 | + | 133 | 239.0 >132.1 (25) | 239.0 >197.0 (29) | 1-200 | 0.9986 | 90.8 (5.3) | 88.1 (5.8) | 86.8 (3.9) | 0.6 | 2 | 16.5 |
| 119 | Cycluron | 7.06 | + | 130 | 199.2 >89.24 (17) | 199.2 >72.31 (25) | 1-200 | 0.9997 | 87.7 (5.6) | 87.6 (6.7) | 88.7 (4.9) | 0.6 | 2 | -10 |
| 120 | Isocarbophos | 7.08 | + | 61 | 307.1 >231.0 (18) | 307.1 > 273.2 (5) | 1-200 | 0.9985 | 89.5 (4.1) | 90.8 (5) | 89.3 (7.3) | 0.6 | 2 | -10.5 |
| 121 | Carboxin | 7.34 | + | 111 | 236.1 >143.0 (18) | 236.1 >87.22 (28) | 1-200 | 0.9995 | 84.2 (3.1) | 83.4 (2.8) | 84.4 (2.8) | 0.6 | 2 | -18 |
| 122 | 3,4,5-Trimethacarb | 7.37 | + | 95 | 194.1 >107.0 (39) | 194.1 >137.1 (32) | 1-200 | 0.9994 | 89.9 (5.2) | 89.9 (6) | 86.7 (5.5) | 0.6 | 2 | 16.3 |
| 123 | Ditalimfos | 7.50 | + | 154 | 300.0 >130.0 (29) | 300.0 >272.0 (14) | 2-200 | 0.9982 | 87.4 (4.2) | 89.6 (6.5) | 89.2 (6) | 1.5 | 5 | 14.7 |
| 124 | Fensulfothion | 7.56 | + | 117 | 309.0 >173.0 (26) | 309.0 >281.0 (11) | 1-200 | 0.9997 | 88.5 (5.7) | 90.6 (5.9) | 89.4 (5.3) | 0.6 | 2 | 19.2 |

| | | | | | | | | | | | | | | |
|---|---|---|---|---|---|---|---|---|---|---|---|---|---|---|
| 125 | Chlorantraniliprole | 7.75 | + | 149 | 481.8 >450.9 (17) | 481.8 >283.9 (13) | 1-200 | 0.9996 | 89.1 (6.9) | 91.1 (4) | 86.4 (5) | 0.6 | 2 | -5.5 |
| 126 | Fenthion-sulfone | 7.88 | + | 129 | 311.0 >143.0 (32) | 311.0 >137.0 (18) | 2-200 | 0.9989 | 87.1 (6) | 89.7 (6.3) | 87.9 (6.2) | 1.5 | 5 | 10.5 |
| 127 | Iodosulfuron-methyl | 7.95 | + | 100 | 529.9 >141.0 (21) | 529.9 >324.9 (12) | 2-200 | 0.9982 | 88.2 (6.2) | 87.2 (5.9) | 89.9 (4.5) | 1.5 | 5 | 16.4 |
| 128 | Phenmedipham | 7.95 | + | 48 | 318.0 >136.1 (25) | 318.0 >168.1 (12) | 1-200 | 0.9999 | 88.6 (5.7) | 88.6 (4) | 89.6 (5.9) | 0.6 | 2 | -9.9 |
| 129 | Bensulfuron-methyl | 7.96 | + | 94 | 411.1 >149.0 (22) | 411.1 >213.1 (30) | 1-200 | 0.9994 | 90.2 (5.8) | 90.7 (5.8) | 90.5 (5.6) | 0.6 | 2 | 19.8 |
| 130 | Imazosulfuron | 7.97 | + | 157 | 413.0 >156.0 (23) | 413.0 >257.9 (33) | 5-200 | 0.9993 | 89.3 (4.7) | 89.3 (5.3) | 89.9 (5.4) | 3 | 10 | 11.5 |
| 131 | Inabenfide | 7.99 | + | 91 | 339.1 >244.1 (43) | 339.1 >321.0 (12) | 1-200 | 0.9992 | 88.8 (5.5) | 90.5 (3.9) | 92.2 (5.8) | 0.6 | 2 | 14.7 |
| 132 | Thiophanate-methyl | 8.02 | + | 123 | 343.1 >160.0 (43) | 343.1 >311.0 (12) | 2-200 | 0.9987 | 88.5 (5.6) | 90.7 (5) | 88.6 (5) | 1.5 | 5 | -38.1 |
| 133 | Flazasulfuron | 8.04 | + | 122 | 408.0 >182.1 (24) | 408.0 >301.1 (20) | 2-200 | 0.9994 | 87.9 (5) | 87.6 (4.9) | 87.4 (5.7) | 1.5 | 5 | 19.9 |
| 134 | Metominostrobin | 8.05 | + | 155 | 285.1 >196.0 (45) | 285.1 >238.1 (35) | 1-200 | 0.9991 | 89.2 (5.7) | 89.1 (5.3) | 87.6 (5.3) | 0.6 | 2 | 10.4 |
| 135 | Tribenuron-methyl | 8.05 | + | 113 | 396.1 >181.1 (35) | 396.1 >364.0 (34) | 1-200 | 0.9983 | 89.8 (5.5) | 92.9 (4.1) | 90.2 (3.9) | 0.6 | 2 | 16.8 |
| 136 | Fenobucarb | 8.09 | + | 99 | 208.2 >95.24 (18) | 208.2 >152.1 (13) | 1-200 | 0.9998 | 89.4 (3.3) | 90.8 (5.6) | 87 (4.5) | 0.6 | 2 | -11.1 |
| 137 | Fosthiazate | 8.11 | + | 113 | 284.1 >104.0 (21) | 284.1 >228.0 (11) | 1-100 | 0.9992 | 89.5 (5) | 90.2 (6.2) | 90.4 (6.4) | 0.6 | 2 | 10.6 |
| 138 | Penoxsulam | 8.15 | + | 136 | 484.1 >164.1 (31) | 484.1 >444.1 (10) | 1-200 | 0.9996 | 87.7 (4) | 91.1 (5.8) | 90.2 (6) | 0.6 | 2 | 17.4 |
| 139 | Desmedipham | 8.20 | + | 101 | 318.0 >182.1 (16) | 318.0 >136.1 (29) | 1-200 | 0.9995 | 86 (4.8) | 91.4 (5) | 89.5 (5.2) | 0.6 | 2 | -9.6 |
| 140 | Dimefuron | 8.23 | + | 111 | 339.1 >167.1 (38) | 339.1 >295.1 (35) | 1-200 | 0.9984 | 90.4 (6.3) | 87.5 (5.1) | 91.8 (4.3) | 0.6 | 2 | 8.7 |
| 141 | Propazine | 8.24 | + | 157 | 230.1 >79.1 (22) | 230.1 >146.1 (33) | 1-200 | 0.9985 | 88 (5.7) | 90.8 (3.4) | 91.3 (4.2) | 0.6 | 2 | 19.8 |
| 142 | Thionazin | 8.24 | + | 141 | 249.0 >97.0 (20) | 249.0 >129.9 (32) | 1-200 | 0.9997 | 90.8 (6) | 85.7 (4.8) | 87.3 (4.5) | 0.6 | 2 | 18.7 |
| 143 | Chlorotoluron | 8.27 | + | 121 | 213.1 >72.31 (21) | 213.1 >140.0 (27) | 1-200 | 0.9996 | 89.5 (6.4) | 87.3 (4.5) | 89.8 (5.8) | 0.6 | 2 | -9.4 |
| 144 | Fenpropidin | 8.34 | + | 112 | 274.2 >86.1 (20) | 274.2 >147.1 (25) | 1-200 | 0.9997 | 90.8 (5.5) | 89.1 (6.8) | 92.9 (4.5) | 0.6 | 2 | 17.4 |
| 145 | Diclosulam | 8.35 | + | 99 | 406.1 >177.1 (31) | 406.1 >377.1 (35) | 1-200 | 0.9987 | 89.7 (5.9) | 91.6 (5.6) | 89 (5.7) | 0.6 | 2 | 16.4 |
| 146 | Furalaxyl | 8.35 | + | 125 | 302.1 >242.1 (18) | 302.1 >270.0 (11) | 1-200 | 0.9989 | 90.6 (7.1) | 89.4 (5.9) | 89.1 (5.7) | 0.6 | 2 | -8 |
| 147 | Metobromuron | 8.38 | + | 119 | 259.0 >169.9 (22) | 259.0 >170.9 (29) | 1-200 | 0.9993 | 90.2 (5.1) | 91.5 (6.1) | 91.5 (4) | 0.6 | 2 | –7.69 |
| 148 | Ethofumesate | 8.42 | + | 143 | 287.1 >121.2 (20) | 287.1 >259.1 (11) | 1-200 | 0.9992 | 91.2 (6.1) | 89.6 (4.6) | 88.3 (4.3) | 0.6 | 2 | -11.6 |
| 149 | Clodinafop-propargyl | 8.46 | + | 115 | 350.1 >238.1 (32) | 350.1 >266.1 (12) | 2-200 | 0.9987 | 88.5 (5.2) | 93.2 (5.8) | 88.4 (4.5) | 1.5 | 5 | 5.4 |
| 150 | Methabenzthiazuron | 8.50 | + | 98 | 222.1 >165.1 (19) | 222.1 >150.1 (36) | 1-200 | 0.9981 | 90.7 (4.4) | 88.5 (5.6) | 93.6 (3.3) | 0.6 | 2 | -11.8 |
| 151 | Benodanil | 8.51 | + | 89 | 324.1 >231.1 (21) | 324.1 >105.1 (26) | 1-200 | 0.9997 | 89.6 (5.3) | 88.4 (5.2) | 89.5 (5.4) | 0.6 | 2 | 5.4 |
| 152 | Imazalil | 8.57 | + | 187 | 297.1 >159.0 (26) | 297.1 >255.0 (23) | 1-200 | 0.9996 | 70.9 (2.9) | 69.7 (3) | 70.7 (3.2) | 0.6 | 2 | -6.4 |
| 153 | Atrazine | 8.60 | + | 38 | 216.1 >174.0 (20) | 216.1 >104.1 (31) | 1-200 | 0.9993 | 92.4 (4.8) | 91.6 (5.6) | 89 (6.5) | 0.6 | 2 | –16.8 |
| 154 | Terbutryn | 8.60 | + | 151 | 242.1 >186.1 (21) | 242.1 >91.2 (29) | 1-200 | 0.9999 | 90.1 (4.8) | 89.9 (5.5) | 90 (5.8) | 0.6 | 2 | –9.27 |
| 155 | Diethofencarb | 8.62 | + | 92 | 268.2 >124.1 (35) | 268.2 >180.1 (20) | 1-200 | 0.9996 | 88.1 (6.3) | 90.8 (5.8) | 92.2 (6.1) | 0.6 | 2 | –4.97 |
| 156 | Fluazifop-butyl | 8.62 | + | 90 | 384.1 >282.1 (38) | 384.1 >328.1 (29) | 1-200 | 0.9996 | 89.5 (4.9) | 89.1 (4.6) | 87.9 (5.9) | 0.6 | 2 | -6.3 |
| 157 | Disulfoton sulfone | 8.67 | + | 101 | 307.2 >143.1 (29) | 307.2 >262.1 (31) | 1-200 | 0.9998 | 90 (5.5) | 87.6 (4.5) | 88.7 (5.2) | 0.6 | 2 | -8.6 |
| 158 | Phorate-Sulfone | 8.72 | + | 88 | 293.1 >115.1 (29) | 293.1 >199.1 (26) | 1-200 | 0.9993 | 88.9 (5.5) | 88.1 (5.1) | 90.4 (5.5) | 0.6 | 2 | 15.5 |

| | | | | | | | | | | | | | | |
|---|---|---|---|---|---|---|---|---|---|---|---|---|---|---|
| 159 | Halofenozide | 8.82 | + | 76 | 331.0 >275.1 (8) | 331.0 >105.0 (18) | 1-200 | 0.9995 | 87.8 (5.6) | 88.4 (5.3) | 87.7 (5.6) | 0.6 | 2 | –19.1 |
| 160 | Ethiprole | 8.83 | + | 167 | 397.0 >350.9 (23) | 397.0 >254.9 (39) | 1-200 | 0.9988 | 88.8 (5) | 89.9 (5.8) | 90.8 (4.7) | 0.6 | 2 | –18.9 |
| 161 | Dodemorph | 8.86 | + | 90 | 282.3 >98.1 (33) | 282.3 >98.1 (17) | 1-200 | 0.9998 | 87.8 (6.6) | 90.7 (4.5) | 88.7 (6.5) | 0.6 | 2 | 14.7 |
| 162 | Promecarb | 8.88 | + | 92 | 208.1 >109.2 (16) | 208.1 >151.2 (9) | 1-200 | 0.9997 | 87.9 (5.1) | 88.7 (5.5) | 88.5 (6) | 0.6 | 2 | –11.1 |
| 163 | Flutriafol | 8.92 | + | 128 | 302.1 >70.33 (20) | 302.1 >123.1 (30) | 1-200 | 0.9996 | 90.6 (4.6) | 91.1 (4.6) | 85.8 (5.8) | 0.6 | 2 | –5.17 |
| 164 | Terbuthylazine | 8.94 | + | 122 | 230.1 >96.1 (25) | 230.1 >174.1 (24) | 1-200 | 0.9992 | 90.8 (5.8) | 89.2 (5.6) | 86.9 (6.2) | 0.6 | 2 | 16.9 |
| 165 | Isoproturon | 8.99 | + | 133 | 207.2 >72.31 (21) | 207.2 >134.1 (26) | 1-200 | 0.9992 | 86.8 (4.9) | 90.5 (6.2) | 89 (6.9) | 0.6 | 2 | -8.4 |
| 166 | Metalaxyl | 9.07 | + | 116 | 280.2 >220.1 (16) | 280.2 >192.1 (21) | 1-200 | 0.9975 | 88.7 (5.9) | 88.9 (5.7) | 90.7 (6.4) | 0.6 | 2 | -6.7 |
| 167 | Flubendazole | 9.08 | + | 128 | 314.1 >122.9 (36) | 314.1 >282 (22) | 1-200 | 0.9983 | 88.3 (5) | 89.8 (6.3) | 89.9 (5.7) | 0.6 | 2 | 10.5 |
| 168 | Pyrifenox | 9.09 | + | 94 | 295.1 >93.1 (29) | 295.1 >263 (35) | 1-200 | 0.9994 | 86.8 (4.9) | 91.3 (5.3) | 89.1 (5.2) | 0.6 | 2 | 16.7 |
| 169 | Forchlorfenuron | 9.13 | + | 128 | 248.0 >129.1 (20) | 248.0 >93.23 (36) | 1-200 | 0.9997 | 89.1 (6) | 89.2 (6.3) | 87.1 (5.2) | 0.6 | 2 | –4.90 |
| 170 | Diuron | 9.14 | + | 134 | 233.0 >72.33 (21) | 233.0 >46.51 (19) | 1-200 | 0.9988 | 88.6 (5.2) | 88.4 (5.1) | 91.8 (5.4) | 0.6 | 2 | –6.33 |
| 171 | Triadimenol | 9.16 | + | 58 | 296.0 >70.04 (24) | 296.0 >126.9 (43) | 1-200 | 0.9997 | 90.1 (5.4) | 86.7 (7.1) | 89.8 (4.5) | 0.6 | 2 | -12 |
| 172 | Mepronil | 9.33 | + | 137 | 270.2 >119.1 (26) | 270.2 >228.0 (16) | 1-200 | 0.999 | 90.5 (5.2) | 89.9 (6.2) | 90.3 (6) | 0.6 | 2 | –4.91 |
| 173 | Flutolanil | 9.40 | + | 136 | 324.1 >242.0 (28) | 324.1 >262.0 (20) | 1-200 | 0.9968 | 87.7 (6.4) | 88.6 (6) | 86.2 (5.6) | 0.6 | 2 | –7.00 |
| 174 | Mandipropamid | 9.40 | + | 152 | 412.1 >328.1 (15) | 412.1 >365.1 (11) | 1-200 | 0.9995 | 87.8 (5.1) | 86.4 (4.6) | 89.1 (6.9) | 0.6 | 2 | -9.8 |
| 175 | Pyrimethanil | 9.51 | + | 165 | 200.1 >107.1 (25) | 200.1 >168.1 (33) | 1-200 | 0.9996 | 84.1 (4.5) | 87.7 (5.8) | 90.1 (6.2) | 0.6 | 2 | –11.7 |
| 176 | Famphur | 9.53 | + | 111 | 326.0 >217.0 (21) | 326.0 >281.0 (27) | 1-200 | 0.9998 | 89.5 (5.9) | 89.4 (5.4) | 88.3 (5.7) | 0.6 | 2 | 17.4 |
| 177 | Benthiavalicarb-isopropyl | 9.59 | + | 113 | 382.2 >197.0 (23) | 382.2 >296.1 (30) | 1-200 | 0.9986 | 91.4 (3.7) | 89.1 (7.1) | 87.3 (5.6) | 0.6 | 2 | 16.9 |
| 178 | Flurprimidol | 9.70 | + | 111 | 313.1 >91.0 (30) | 313.1 >270.0 (35) | 1-200 | 0.9993 | 90.7 (7.3) | 92 (5.6) | 92 (4.9) | 0.6 | 2 | -6.9 |
| 179 | Ivermectin | 9.73 | + | 76 | 892.7 >569.2 (13) | 892.7 >307.0 (22) | 1-200 | 0.9975 | 104.2 (4) | 101.9 (4.7) | 101.6 (4.4) | 0.6 | 2 | 33.5 |
| 180 | Aziprotryne | 9.80 | + | 174 | 226.1 >89.0 (31) | 226.1 >198.1 (17) | 1-200 | 0.9991 | 90 (5.5) | 89.5 (5.2) | 86.9 (4.2) | 0.6 | 2 | 11 |
| 181 | Bupirimate | 9.88 | + | 185 | 317.2 >166.1 (27) | 317.2 >272.0 (21) | 1-200 | 0.9998 | 89.1 (6.5) | 90.1 (6.8) | 89.1 (5) | 0.6 | 2 | -2.9 |
| 182 | Clomazon | 9.92 | + | 63 | 240.1 >89.1 (47) | 240.1 >125.0 (20) | 1-200 | 0.9994 | 88.4 (5.4) | 90 (5.8) | 90 (6) | 0.6 | 2 | 16.4 |
| 183 | Chloroxuron | 10.00 | + | 164 | 291.1 >72.4 (23) | 291.1 >218.1 (27) | 1-200 | 0.9991 | 87.2 (6.6) | 91.5 (4.9) | 86.6 (5.7) | 0.6 | 2 | –6.80 |
| 184 | Bifenazate | 10.03 | + | 93 | 301.2 >198.1 (5) | 301.2 >152.1 (43) | 1-200 | 0.9984 | 88.8 (4.8) | 91.9 (4.8) | 88.8 (5.9) | 0.6 | 2 | –9.88 |
| 185 | Mepanipyrim | 10.15 | + | 169 | 224.1 >77.27 (39) | 224.1 >106.1 (29) | 1-200 | 0.9991 | 88.5 (4.4) | 91.5 (6.8) | 90 (4.2) | 0.6 | 2 | -11 |
| 186 | Fluoxastrobin | 10.23 | + | 191 | 459.1 >427.1 (18) | 459.1 >188.1 (34) | 1-200 | 0.9994 | 90.8 (5.3) | 87.8 (4.3) | 91.9 (4.1) | 0.6 | 2 | -8.7 |
| 187 | Acibenzolar-S-methyl | 10.26 | + | 89 | 211.1 >136.1 (30) | 211.1 >140.1 (24) | 1-200 | 0.9983 | 90.6 (5.5) | 88.5 (6.5) | 90 (4.6) | 0.6 | 2 | –10.7 |
| 188 | Butafenacil | 10.26 | + | 144 | 492.1 >331.0 (26) | 492.1 >348.9 (17) | 1-200 | 0.9997 | 90.3 (4.3) | 90.6 (5) | 86.4 (3.6) | 0.6 | 2 | -8.8 |
| 189 | Saflufenacil | 10.26 | + | 101 | 501.2 >170.0 (13) | 501.2 >200.0 (13) | 1-200 | 0.9986 | 91.2 (4.8) | 90.9 (6) | 91.6 (5) | 0.6 | 2 | 10.5 |
| 190 | Linuron | 10.48 | + | 112 | 249.0 >182.0 (18) | 249.0 >160.0 (21) | 1-200 | 0.9985 | 89.1 (5.6) | 91.4 (4.9) | 91.1 (5.2) | 0.6 | 2 | -14.6 |
| 191 | Fenpropimorph | 10.59 | + | 153 | 304.2 >116.9 (55) | 304.2 >147.0 (30) | 1-200 | 0.9999 | 92.4 (4.7) | 88.7 (4.9) | 92.1 (5.4) | 0.6 | 2 | -8.7 |
| 192 | Tetraconazole | 10.60 | + | 143 | 371.9 >159.1 (28) | 371.9 >70.33 (24) | 1-200 | 0.9985 | 91.4 (4.7) | 88.2 (5.7) | 89.2 (6.2) | 0.6 | 2 | 16.3 |

| | | | | | | | | | | | | | | |
|---|---|---|---|---|---|---|---|---|---|---|---|---|---|---|
| 193 | Valifenalate | 10.63 | + | 131 | 399.1 >197.1 (23) | 399.1 >313.1 (17) | 1-200 | 0.9981 | 90.9 (5.9) | 90.5 (4.7) | 88.6 (6.2) | 0.6 | 2 | 16 |
| 194 | MCPA | 10.68 | + | 116 | 202.1 >126.9 (16) | 202.1 >163.8 (16) | 1-200 | 0.9988 | 88.8 (5.8) | 91.4 (5.4) | 86.5 (4.9) | 0.6 | 2 | 17.7 |
| 195 | 2,4-D | 10.70 | – | 125 | 217.9 >132.8 (27) | 217.9 >161.9 (17) | 1-200 | 0.9986 | 89.1 (5.7) | 88.1 (5.3) | 90.3 (6.3) | 0.6 | 2 | 15.2 |
| 196 | Dicamba | 10.70 | – | 118 | 216.0 >35.0 (29) | 216.0 >159.8 (17) | 1-200 | 0.9983 | 87.9 (4.5) | 93.9 (5.5) | 88.3 (3.3) | 0.6 | 2 | 16.9 |
| 197 | Fenamiphos | 10.71 | + | 94 | 304.1 >234.0 (37) | 304.1 >276.0 (21) | 1-200 | 0.9983 | 89.6 (6.5) | 86.6 (5.6) | 89.4 (5.6) | 0.6 | 2 | 16.8 |
| 198 | Azoxystrobin | 10.72 | + | 144 | 404.1 >372.0 (16) | 404.1 >344.0 (27) | 1-200 | 0.9985 | 89.3 (5) | 91.2 (4.9) | 92.3 (5) | 0.6 | 2 | -7.9 |
| 199 | Uniconazole | 10.73 | + | 114 | 292.1 >125.0 (32) | 292.1 >218.0 (15) | 1-200 | 0.9981 | 90.1 (5.4) | 88.3 (4.9) | 87.4 (6) | 0.6 | 2 | 10.3 |
| 200 | Fenarimol | 10.78 | + | 99 | 331.0 >259.0 (24) | 331.0 >304.0 (34) | 1-200 | 0.9996 | 89.7 (6) | 91.9 (4.2) | 89.5 (4.6) | 0.6 | 2 | -6.5 |
| 201 | Ethoxysulfuron | 10.80 | + | 108 | 399.2 >182.0 (40) | 399.2 >261.0 (33) | 1-200 | 0.9988 | 88.5 (4.6) | 88.2 (5.6) | 87.8 (4.8) | 0.6 | 2 | 16.5 |
| 202 | Methiocarb | 10.84 | + | 84 | 226.1 >169.0 (10) | 226.1 >121.0 (36) | 1-200 | 0.9994 | 87.8 (6.5) | 90.2 (5.5) | 88.3 (4.9) | 0.6 | 2 | –8.68 |
| 203 | Nuarimol | 10.85 | + | 166 | 315.1 >252.1 (23) | 315.1 >242.9 (26) | 1-200 | 0.9997 | 86.6 (5.2) | 87.6 (5.2) | 88.8 (5.8) | 0.6 | 2 | -6 |
| 204 | Cyazofamid | 10.87 | + | 103 | 325.1 >108.1 (17) | 325.1 >217.0 (21) | 1-200 | 0.9993 | 86.3 (4.3) | 89.7 (4.9) | 90.5 (4.7) | 0.6 | 2 | -8.9 |
| 205 | Chlorimuron-ethyl | 10.92 | + | 124 | 415.0 >186.0 (45) | 415.0 >213.0 (35) | 5-200 | 0.9987 | 89.9 (5.5) | 89.3 (5.7) | 91.3 (4.4) | 3 | 10 | 16.9 |
| 206 | Prometryn | 10.93 | + | 179 | 242.2 >158.1 (25) | 242.2 >200.1 (20) | 1-200 | 0.9999 | 90.3 (5.5) | 89.8 (6.5) | 88 (5.9) | 0.6 | 2 | –8.03 |
| 207 | Rotenone | 11.01 | + | 206 | 395.1 >213.0 (26) | 395.1 >191.1 (39) | 1-200 | 0.9983 | 88.7 (6.2) | 87.2 (5.2) | 90.7 (4.5) | 0.6 | 2 | -5.6 |
| 208 | Epoxiconazole | 11.03 | + | 55 | 330.1 >121.1 (15) | 330.1 >101.2 (35) | 1-200 | 0.9993 | 88.4 (5.2) | 87.3 (3.5) | 92.5 (6.3) | 0.6 | 2 | –30.1 |
| 209 | Fenamidone | 11.05 | + | 115 | 312.0 >92.2 (32) | 312.0 >236.2 (15) | 1-200 | 0.9992 | 89.7 (5.8) | 88.2 (5.7) | 92.5 (5.1) | 0.6 | 2 | 5.5 |
| 210 | Diflubenzuron | 11.10 | + | 126 | 311.1 >157.9 (14) | 311.1 >141.1 (25) | 1-200 | 0.9996 | 89.8 (6) | 87.2 (4.4) | 91.5 (5.3) | 0.6 | 2 | –8.48 |
| 211 | Dimoxystrobin | 11.15 | + | 105 | 327.1 >116.1 (20) | 327.1 >238.1 (13) | 1-200 | 0.9997 | 89.5 (4.4) | 88.6 (5.7) | 89.1 (6.2) | 0.6 | 2 | -9.4 |
| 212 | Picoxystrobin | 11.29 | + | 90 | 368.1 >145.3 (22) | 368.1 >115.2 (50) | 1-200 | 0.9996 | 91.4 (5.7) | 89.7 (6.7) | 90.5 (5.3) | 0.6 | 2 | -4.4 |
| 213 | Propiconazole | 11.29 | + | 91 | 342.4 >152.3 (26.0) | 342.4 >305.7 (5.0) | 1-200 | 0.9992 | 92.1 (4.8) | 88.8 (4.1) | 85.6 (5.1) | 0.6 | 2 | 5.2 |
| 214 | Tebufenozide | 11.29 | + | 96 | 353.2 >133.1 (21) | 353.2 >297.2 (5) | 1-200 | 0.9994 | 88.8 (6.2) | 88.4 (3.9) | 90.9 (5.8) | 0.6 | 2 | –7.94 |
| 215 | Neburon | 11.31 | + | 121 | 275.0 >88.22 (14) | 275.0 >57.39 (18) | 1-200 | 0.9988 | 87.4 (5.6) | 91.2 (4.6) | 87.3 (5.5) | 0.6 | 2 | –7.39 |
| 216 | Carfentrazone-ethyl | 11.33 | + | 180 | 412.1 >346.1 (23) | 412.1 >366.1 (19) | 1-200 | 0.9986 | 85.6 (4.1) | 90.8 (6.3) | 91.2 (5.6) | 0.6 | 2 | 1.26 |
| 217 | Fludioxonil | 11.34 | + | 70 | 266.0 >228.9 (10) | 266.0 >157.9 (32) | 1-200 | 0.998 | 88.2 (4.9) | 87.6 (6.3) | 93.1 (4.5) | 0.6 | 2 | –13.1 |
| 218 | Clothianidin | 11.36 | + | 101 | 250.1 >169.1 (13) | 250.1 >132.2 (14) | 1-200 | 0.9982 | 91.3 (5) | 90.8 (5.6) | 85.3 (3.1) | 0.6 | 2 | –23.2 |
| 219 | Thiram | 11.38 | + | 130 | 241.1 >169.7 (10) | 241.1 >204.8 (14) | 5-200 | 0.9991 | 87.7 (6.1) | 90.7 (5.6) | 89.7 (7.5) | 3 | 10 | 12.5 |
| 220 | 2,4-DB | 11.41 | – | 120 | 247.0 >160.8 (5) | 247.0 >169.0 (33) | 1-200 | 0.9986 | 90.1 (4.8) | 87.5 (5.6) | 87.4 (5) | 0.6 | 2 | -16.3 |
| 221 | Molinate | 11.41 | + | 87 | 188.1 >126.1 (24) | 188.1 >55.0 (13) | 1-200 | 0.9987 | 87.4 (4.6) | 88.7 (4) | 91.3 (4) | 0.6 | 2 | 6.5 |
| 222 | Paclobutrazol | 11.47 | + | 145 | 294.1 >70.32 (22) | 294.1 >125.1 (36) | 1-200 | 0.9997 | 87.3 (5.3) | 89.4 (6.7) | 91.1 (4.7) | 0.6 | 2 | -14.1 |
| 223 | Isoprothiolane | 11.50 | + | 68 | 291.1 >189.0 (22) | 291.1 >231.0 (11) | 1-200 | 0.9988 | 91 (5.9) | 90 (5.9) | 88.8 (6.8) | 0.6 | 2 | 10.9 |
| 224 | Fluopicolide | 11.64 | + | 150 | 385.0 >172.9 (27) | 385.0 >174.8 (24) | 1-200 | 0.9982 | 89.2 (5.9) | 85.6 (5.2) | 91.6 (4.9) | 0.6 | 2 | 11.4 |
| 225 | Dimethomorph | 11.75 | + | 167 | 388.1 >165.1 (31) | 388.1 >301.1 (21) | 1-200 | 0.9992 | 92.2 (4.9) | 92.2 (3.4) | 88.8 (6.2) | 0.6 | 2 | 15.6 |
| 226 | Methoxyfenozide | 11.78 | + | 95 | 369.1 >149.1 (18) | 369.1 > 313.1 (6) | 1-200 | 0.9982 | 91.4 (5.7) | 89 (5) | 88.8 (6.5) | 0.6 | 2 | -8.9 |

| | | | | | | | | | | | | | | |
|---|---|---|---|---|---|---|---|---|---|---|---|---|---|---|
| 227 | Cyproconazole | 11.79 | + | 157 | 247.0 >125.9 (3) | 247.0 >180.0 (28) | 1-200 | 0.9995 | 90.9 (5.2) | 88.5 (4.9) | 87.5 (6.1) | 0.6 | 2 | 14.5 |
| 228 | Diclobutrazol | 11.79 | + | 169 | 328.1 >70.3 (32) | 328.1 >159.1 (34) | 1-200 | 0.9992 | 88.4 (5.8) | 89 (4.9) | 92.6 (5.3) | 0.6 | 2 | −9.21 |
| 229 | Triadimefon | 11.84 | + | 103 | 294.1 >197.1 (14) | 294.1 >224.9 (12) | 1-200 | 0.9995 | 89.7 (4) | 88.7 (6.3) | 90.2 (6.1) | 0.6 | 2 | −3.64 |
| 230 | Fluxapyroxad | 11.87 | + | 157 | 382.3 >341.9 (21) | 382.3 >362.1 (13) | 1-200 | 0.9998 | 92.2 (5.1) | 88.1 (6) | 89.7 (6) | 0.6 | 2 | 11.4 |
| 231 | Prothioconazole | 11.91 | + | 70 | 341.9 >306.1 (15) | 341.9 >308.1 (15) | 5-200 | 0.9989 | 88.6 (5.8) | 88.9 (5.9) | 90.1 (5) | 3 | 10 | −69.3 |
| 232 | Zoxamide | 11.94 | + | 159 | 336.1 >186.9 (20) | 336.1 >159.1 (42) | 1-200 | 0.9996 | 88.6 (5.8) | 90.2 (4.5) | 89.1 (6.6) | 0.6 | 2 | −7.27 |
| 233 | Mefenacet | 11.98 | + | 111 | 299.1 >148.3 (16) | 299.1 >120.3 (27) | 1-200 | 0.9992 | 90.1 (5.1) | 88.4 (5.8) | 88.6 (4.8) | 0.6 | 2 | −6.24 |
| 234 | Flufenacet | 12.05 | + | 96 | 364.0 >152.1 (19) | 364.0 >194.1 (11) | 1-200 | 0.9994 | 90.5 (5.9) | 90.4 (5.6) | 85.8 (3.8) | 0.6 | 2 | −8.69 |
| 235 | Myclobutanil | 12.08 | + | 142 | 289.1 >70.31 (21) | 289.1 >125.1 (33) | 1-200 | 0.9996 | 89.7 (4.9) | 91.7 (4.9) | 89.3 (5.6) | 0.6 | 2 | −2.88 |
| 236 | Dimethylvinphos | 12.18 | + | 110 | 332.2 >127.0 (13) | 332.2 >170.0 (36) | 2-200 | 0.9988 | 89.5 (6.8) | 88.1 (5.2) | 90.5 (4.3) | 1.5 | 5 | 17.4 |
| 237 | Fluquinconazole | 12.30 | + | 130 | 376.1 >349.0 (19) | 376.1 >307.1 (22) | 1-200 | 0.9982 | 86.9 (4.7) | 86.5 (4.7) | 89.7 (5.9) | 0.6 | 2 | −10.8 |
| 238 | Famoxadone | 12.38 | + | 90 | 392.1 >331.1 (10) | 392.1 >238.1 (20) | 1-200 | 0.9981 | 92.9 (4.5) | 85.7 (3.9) | 89 (4.7) | 0.6 | 2 | −15.6 |
| 239 | Iprovalicarb | 12.38 | + | 91 | 321.2 >119.1 (19) | 321.2 >203.2 (9) | 1-200 | 0.9982 | 90.1 (6.3) | 91.3 (4.9) | 90.1 (6.1) | 0.6 | 2 | 10.4 |
| 240 | Benzovindiflupyr | 12.54 | + | 159 | 399.0 >173.0 (30) | 399.0 >209.9 (22) | 2-200 | 0.9994 | 91 (4.2) | 90.2 (5.5) | 89.3 (5.1) | 1.5 | 5 | 19.8 |
| 241 | Tebutam | 12.54 | + | 113 | 234.1 >91.1 (30) | 234.1 >192.1 (10) | 1-200 | 0.9998 | 90 (5.7) | 90.1 (4.7) | 90.2 (5.1) | 0.6 | 2 | 14.5 |
| 242 | Thiobencarb | 12.54 | + | 105 | 258.1 >125.1 (21) | 258.1 >89.22 (50) | 1-200 | 0.9987 | 92.1 (4.4) | 90.3 (6.2) | 90.6 (7) | 0.6 | 2 | -12.6 |
| 243 | Benzoximate | 12.55 | + | 80 | 364.0 >105.1 (9) | 364.0 >105.1 (23) | 1-200 | 0.9995 | 87.5 (5) | 89.4 (5.6) | 91.1 (3.9) | 0.6 | 2 | −11.4 |
| 244 | Triflumuron | 12.55 | + | 118 | 359.0 >156.0 (13) | 359.0 >138.9 (27) | 1-200 | 0.9991 | 90.3 (5.1) | 88.1 (4.1) | 89.8 (6.2) | 0.6 | 2 | −22.3 |
| 245 | Cymoxanil | 12.56 | + | 123 | 199.1 >183.9 (16) | 199.1 >141.1 (25) | 1-200 | 0.9994 | 89.6 (6) | 91.6 (6.8) | 91.1 (5.2) | 0.6 | 2 | −11.0 |
| 246 | Fluopyram | 12.56 | + | 146 | 394.1 >72.9 (26) | 394.1 >190.0 (19) | 1-200 | 0.9988 | 90.4 (6.5) | 90.7 (6.3) | 90.7 (6.8) | 0.6 | 2 | 18.7 |
| 247 | Fenbuconazole | 12.61 | + | 124 | 337.1 >125.1 (43) | 337.1 >194.1 (28) | 1-200 | 0.999 | 87.1 (6.4) | 89.5 (5.7) | 87.1 (4.9) | 0.6 | 2 | -6.5 |
| 248 | Spirotetramat | 12.62 | + | 152 | 374.2 >302.1 (16) | 374.2 >330.2 (10) | 1-200 | 0.9996 | 89.4 (5.3) | 89 (5.1) | 86.1 (3.7) | 0.6 | 2 | -6.1 |
| 249 | Ethoprophos | 12.63 | + | 98 | 243.1 >131.0 (21) | 243.1 >215.0 (12) | 1-200 | 0.9984 | 89.3 (2.9) | 89.5 (5.4) | 89.7 (7.2) | 0.6 | 2 | 12.6 |
| 250 | Fenhexamid | 12.63 | + | 125 | 302.1 >97.25 (26) | 302.1 >55.42 (39) | 1-200 | 0.9996 | 90.2 (5) | 91.1 (5.2) | 89.8 (6.8) | 0.6 | 2 | -8.2 |
| 251 | Penconazole | 12.66 | + | 61 | 284.1 >159.1 (43) | 284.1 >173.1 (10) | 1-200 | 0.9996 | 89.9 (4.8) | 89.7 (4.5) | 90.3 (6.5) | 0.6 | 2 | −18.9 |
| 252 | Cyprodinil | 12.70 | + | 178 | 226.2 >93.22 (37) | 226.2 >118.1 (34) | 1-200 | 0.9997 | 88.6 (4.9) | 88.5 (5.1) | 87.7 (3.6) | 0.6 | 2 | 23.2 |
| 253 | Spinosad A (*) | 12.93 | + | 250 | 732.4 >142.2 (26) | 732.4 >98.26 (41) | 1-200 | 0.9983 | 86.9 (2.5) | 84.9 (4) | 84 (2.8) | 0.6 | 2 | -5.9 |
| 254 | Oryzalin | 13.07 | − | 182 | 344.9 >147.0 (25) | 344.9 >281.2 (18) | 2-200 | 0.9981 | 88.1 (4.5) | 91.6 (5.1) | 91.7 (6.1) | 1.5 | 5 | -4.6 |
| 255 | Fenothiocarb | 13.35 | + | 83 | 254.1 >72.1 (18) | 254.1 >160.1 (10) | 1-200 | 0.998 | 90 (4.5) | 85.9 (4.5) | 92.7 (4.1) | 0.6 | 2 | 2.8 |
| 256 | Febuconazole | 13.37 | + | 155 | 337.0 >70.0 (21) | 337.0 >125.0 (35) | 1-200 | 0.9986 | 91.6 (5) | 89.6 (5.8) | 88.7 (4.7) | 0.6 | 2 | 15.2 |
| 257 | Bitertanol | 13.43 | + | 40 | 338.2 >269.2 (9) | 338.2 >70.1 (12) | 1-200 | 0.998 | 89.5 (7.2) | 88.7 (6.2) | 91.7 (6.8) | 0.6 | 2 | −5.78 |
| 258 | Indoxacarb | 13.50 | + | 184 | 528.0 >203.0 (38) | 528.0 >150.0 (25) | 1-200 | 0.9995 | 90.1 (5.8) | 90.9 (6.3) | 87.2 (6.2) | 0.6 | 2 | −15.3 |
| 259 | Flusilazole | 13.58 | + | 188 | 316.0 >247.0 (20) | 316.0 >165.1 (33) | 1-200 | 0.9986 | 91.7 (5.7) | 90.1 (2.8) | 89.5 (4.6) | 0.6 | 2 | −9.07 |
| 260 | Fenoxycarb | 13.64 | + | 108 | 317.1 >88.2 (19) | 317.1 >116.0 (11) | 1-200 | 0.9988 | 93.2 (5.7) | 88.8 (5.1) | 89 (5.1) | 0.6 | 2 | -14.9 |

| | | | | | | | | | | | | | | |
|---|---|---|---|---|---|---|---|---|---|---|---|---|---|---|
| 261 | Pycoxystrobin | 13.66 | + | 80 | 368.1 >145.0 (22) | 368.1 >205.0 (10) | 1-200 | 0.9991 | 89.3 (5.8) | 92.2 (6.4) | 90.9 (5.2) | 0.6 | 2 | 19.7 |
| 262 | Terbufenozide | 13.67 | + | 115 | 353.1 >133.0 (18.0) | 353.1 >297.0 (8) | 1-200 | 0.9996 | 89.9 (5.3) | 91.6 (5.6) | 87.4 (5.5) | 0.6 | 2 | 8.2 |
| 263 | Iprobenfos | 13.70 | + | 102 | 289.1 >91.1 (22) | 289.1 >205.0 (11) | 1-200 | 0.9998 | 89.9 (5.6) | 91.4 (5.6) | 89.6 (5.4) | 0.6 | 2 | 11.9 |
| 264 | Triticonazole | 13.77 | + | 143 | 289.1 >91.1 (19) | 289.1 >205.0 (45) | 1-200 | 0.9993 | 90 (6.7) | 87.3 (5.7) | 87.5 (5.1) | 0.6 | 2 | -7.3 |
| 265 | Spinosad D (*) | 13.80 | + | 250 | 746.4 >142.2 (27) | 746.4 >98.26 (43) | 1-200 | 0.9994 | 95.2 (2.1) | 95.5 (2.5) | 94.1 (2.6) | 0.6 | 2 | -9.5 |
| 266 | Fipronil | 13.81 | + | 71 | 436.9 >367.8 (17) | 436.9 >290.1 (25) | 1-200 | 0.9983 | 87.6 (6.1) | 87.4 (6.8) | 85.8 (3.8) | 0.6 | 2 | -16.6 |
| 267 | Buprofezin | 13.86 | + | 112 | 306.2 >106.2 (28) | 306.2 >57.44 (25) | 1-200 | 0.9996 | 89.1 (4.1) | 88.8 (5.4) | 90.7 (5.3) | 0.6 | 2 | -15.7 |
| 268 | Etrimfos | 13.88 | + | 132 | 293.0 >125.0 (26) | 293.0 >265.1 (18) | 1-200 | 0.9985 | 87.4 (4) | 88.8 (5.1) | 90.1 (6.7) | 0.6 | 2 | 16.8 |
| 269 | Kresoxim-methyl | 13.90 | + | 80 | 314.0 >222.1 (15) | 314.0 >235.0 (19) | 1-200 | 0.9993 | 90.9 (4.9) | 89.6 (5.9) | 87.8 (5.3) | 0.6 | 2 | -9.8 |
| 270 | Aclonifen | 13.91 | + | 138 | 265.1 >194.1 (22) | 265.1 >247.1 (10) | 5-200 | 0.9987 | 87.8 (5) | 92.1 (6.1) | 90.9 (5.4) | 3 | 10 | 11 |
| 271 | Mecarbam | 13.91 | + | 107 | 330.1 >116.1 (21) | 330.1 >199.1 (28) | 1-200 | 0.9983 | 91.3 (6.2) | 90.5 (5.5) | 88.7 (5.3) | 0.6 | 2 | -16.9 |
| 272 | Bromobutide | 13.92 | + | 102 | 312.1 >119.1 (34) | 312.1 >194.1 (24) | 1-200 | 0.9982 | 89.1 (4.6) | 89.5 (5.1) | 90.7 (5.1) | 0.6 | 2 | -7.6 |
| 273 | Fluthiacet-Methyl | 13.97 | + | 144 | 404.1 >105.1 (31) | 404.1 >344.1 (35) | 1-200 | 0.9987 | 90.6 (4.6) | 90.8 (4.2) | 90.7 (6.1) | 0.6 | 2 | 11.9 |
| 274 | Diethatyl-ethyl | 13.99 | + | 81 | 262.0 >188.2 (28) | 262.0 >160.2 (11) | 1-200 | 0.9993 | 89.1 (4.4) | 89.8 (4.5) | 89.2 (5.2) | 0.6 | 2 | 18.7 |
| 275 | Haloxyfop | 13.99 | – | 124 | 359.9 >252.0 (23) | 359.9 >288.0 (13) | 2-200 | 0.9992 | 88.2 (5.6) | 88.6 (5.5) | 88.3 (6.9) | 1.5 | 5 | 16.6 |
| 276 | Tebufenpyrad | 14.12 | + | 207 | 334.1 >145.0 (27) | 334.1 >117.1 (25) | 1-200 | 0.9996 | 90.9 (5.1) | 89 (5.7) | 89.3 (5.9) | 0.6 | 2 | –22.9 |
| 277 | Flubendiamide | 14.22 | + | 166 | 680.9 >253.9 (26) | 680.9 >274.0 (15) | 1-200 | 0.9993 | 91.2 (6) | 90.7 (5.3) | 89.3 (6.2) | 0.6 | 2 | 6.9 |
| 278 | Benalaxyl | 14.26 | + | 128 | 326.2 >148.1 (24) | 326.2 >121.1 (32) | 1-200 | 0.9997 | 91.3 (3.5) | 90.7 (4.3) | 88.6 (6.4) | 0.6 | 2 | –9.16 |
| 279 | Tebuconazole | 14.26 | + | 160 | 308.2 >70.33 (24) | 308.2 >125.1 (30) | 1-200 | 0.9993 | 90.7 (6.7) | 89.3 (5.6) | 87.8 (5.2) | 0.6 | 2 | -1.8 |
| 280 | Anilofos | 14.29 | + | 129 | 368.0 >124.9 (31) | 368.0 >199.0 (14) | 1-200 | 0.9993 | 92 (6.3) | 88.3 (4.8) | 90.1 (6.1) | 0.6 | 2 | 11.8 |
| 281 | Amitraz | 14.33 | + | 154 | 294.2 >148.3 (16) | 294.2 >91.2 (40) | 1-200 | 0.9998 | 92.3 (4.1) | 88.4 (4.7) | 90.9 (4.3) | 0.6 | 2 | –8.82 |
| 282 | Piperonyl butoxide | 14.34 | + | 107 | 356.2 >177.1 (12) | 356.2 >119.2 (37) | 1-200 | 0.9998 | 89.6 (6) | 87.7 (5.8) | 89.7 (6.1) | 0.6 | 2 | -15.6 |
| 283 | Isoxaflutole | 14.42 | + | 146 | 360.1 >244.0 (23) | 360.1 >276.0 (15) | 5-200 | 0.9999 | 89.8 (5.4) | 90.7 (5.5) | 89.1 (5.9) | 3 | 10 | -18.5 |
| 284 | Boscalid | 14.44 | + | 189 | 343.0 > 307 (21) | 343.0 > 272.0 (34) | 1-200 | 0.9996 | 89.8 (5.4) | 88.3 (6.7) | 91.6 (5.3) | 0.6 | 2 | -1.5 |
| 285 | Clethodim | 14.44 | + | 147 | 360.2 >244.0 (23) | 360.2 >276.0 (15) | 1-200 | 0.9987 | 88.1 (4.9) | 89.1 (6.4) | 91.4 (5.1) | 0.6 | 2 | 17.3 |
| 286 | Prochloraz | 14.60 | + | 111 | 376.1 >307.9 (14) | 376.1 >70.34 (27) | 1-200 | 0.9996 | 89.8 (4.4) | 88.1 (5.5) | 88.9 (5.2) | 0.6 | 2 | 9.6 |
| 287 | Emamectin benzoate (B1b) (*) | 14.63 | + | 76 | 872.4 >158.1 (37) | 872.4 >82.27 (46) | 1-200 | 0.9983 | 91.6 (5.9) | 90.4 (5.3) | 88.8 (6.6) | 0.6 | 2 | -9.6 |
| 288 | Triphenyl phosphate | 14.69 | + | 201 | 327.1 >151.9 (38) | 327.1 >214.9 (26) | 1-200 | 0.9982 | 91.8 (6.8) | 93 (3.8) | 91.9 (4.2) | 0.6 | 2 | 11.6 |
| 289 | Teflubenzuron | 14.76 | – | 95 | 378.9 >338.8 (10) | 378.9 >358.8 (10) | 1-200 | 0.9946 | 87.9 (5) | 89.1 (5.5) | 90.5 (5) | 0.6 | 2 | -22.8 |
| 290 | Phoxim | 14.77 | + | 75 | 299.1 >129.1 (13) | 299.1 >77.30 (29) | 1-200 | 0.998 | 89.7 (5.1) | 88.8 (5.4) | 90.6 (7.1) | 0.6 | 2 | -12.1 |
| 291 | Pyriproxyfen | 14.77 | + | 121 | 322.1 >96.23 (19) | 322.1 >185.1 (25) | 1-200 | 0.9995 | 91.3 (7.1) | 90.6 (4.2) | 90 (7) | 0.6 | 2 | -42.3 |
| 292 | Clofentezine | 14.80 | + | 100 | 303.0 >138.0 (11) | 303.0 >102.1 (31) | 1-200 | 0.9983 | 89.7 (6.2) | 90.5 (3.5) | 89.3 (5.4) | 0.6 | 2 | -28 |
| 293 | Pyraclostrobin | 14.80 | + | 126 | 388.1 >163.1 (26) | 388.1 >149.0 (31) | 1-200 | 0.9997 | 91.3 (5.1) | 87.9 (5.3) | 90.9 (6.1) | 0.6 | 2 | -6.8 |
| 294 | Fluazinam | 14.81 | – | 158 | 462.9 >415.9 (22) | 462.9 >397.9 (19) | 1-200 | 0.9995 | 90.8 (5.8) | 91.1 (5.8) | 88.8 (4.1) | 0.6 | 2 | -48.2 |

| | | | | | | | | | | | | | | |
|---|---|---|---|---|---|---|---|---|---|---|---|---|---|---|
| 295 | Hexaconazole | 14.83 | + | 161 | 314.2 >70.4 (18) | 314.2 >159.0 (28) | 1-200 | 0.9993 | 86.7 (4.4) | 87.4 (4.5) | 88.6 (5.5) | 0.6 | 2 | -4.6 |
| 296 | Metconazole | 14.86 | + | 179 | 320.1 >70.33 (24) | 320.1 >125.1 (40) | 1-200 | 0.9996 | 86.9 (6.5) | 90.4 (5.2) | 89.3 (6.9) | 0.6 | 2 | -38.5 |
| 297 | Temephos | 14.93 | + | 213 | 467.0 >418.9 (22) | 467.0 >404.9 (17) | 1-200 | 0.9992 | 88.8 (5.5) | 90.5 (4.3) | 90.6 (5.8) | 0.6 | 2 | -50 |
| 298 | Hexythiazox | 15.01 | + | 122 | 353.1 >227.9 (15) | 353.1 >168.1 (24) | 1-200 | 0.9997 | 90.2 (6.8) | 88.3 (4.8) | 89.9 (5.2) | 0.6 | 2 | -47.3 |
| 299 | Metaflumizone | 15.03 | + | 177 | 507.1 >177.9 (23) | 507.1 >286.9 (230 | 1-200 | 0.9992 | 86.8 (5.4) | 88.4 (5.6) | 87.9 (6.5) | 0.6 | 2 | -65.3 |
| 300 | Quinoxyfen | 15.05 | + | 213 | 308.0 >197.0 (35) | 308.0 >162.0 (48) | 1-200 | 0.9993 | 88.9 (5) | 90.2 (4.3) | 89.5 (4.9) | 0.6 | 2 | -47.2 |
| 301 | Metrafenone | 15.20 | + | 121 | 109.1 >209.0 (14) | 109.1 >226.9 (18) | 1-200 | 0.9983 | 90.1 (6.3) | 90.3 (5.1) | 87.2 (5.3) | 0.6 | 2 | 19.8 |
| 302 | Lufenuron | 15.21 | – | 76 | 509.0 >325.9 (10) | 509.0 >175.1 (20) | 1-200 | 0.997 | 108.9 (6.2) | 114.6 (4.8) | 111.3 (6.7) | 0.6 | 2 | -41.6 |
| 303 | Emamectin benzoate (B1a) (*) | 15.23 | + | 76 | 886.5 >158.1 (39) | 886.5 >82.30 (47) | 1-200 | 0.9992 | 87.7 (5.1) | 90 (5.3) | 89.1 (5.2) | 0.6 | 2 | 19.6 |
| 304 | Pencycuron | 15.34 | + | 167 | 329.1 >125.2 (21) | 329.1 >218.1 (16) | 1-200 | 0.9992 | 88.4 (4.9) | 88.1 (5.5) | 88.8 (6.4) | 0.6 | 2 | -22.2 |
| 305 | Cadusafos | 15.41 | + | 92 | 271.0 >159.0 (14) | 271.0 >215.0 (9.8) | 1-200 | 0.9981 | 87.5 (5.4) | 92.1 (5.3) | 89.5 (5.8) | 0.6 | 2 | 11.6 |
| 306 | Diniconazole | 15.45 | + | 115 | 326.0 >70.0 (26) | 326.0 >159.0 (31) | 1-200 | 0.9986 | 87.6 (4.9) | 90.4 (5.9) | 88.2 (4.9) | 0.6 | 2 | -7.7 |
| 307 | Spiromesifen | 15.47 | + | 76 | 371.3 >273.3 (15) | 371.3 >255.2 (20) | 1-200 | 0.9994 | 89.3 (6.5) | 90.9 (4.9) | 91.7 (6.5) | 0.6 | 2 | -25.6 |
| 308 | Propargite | 15.48 | + | 111 | 368.4 >107.1 (28) | 368.4 >57.44 (23) | 1-200 | 0.9984 | 88.7 (6.1) | 90.5 (5.8) | 91 (4.9) | 0.6 | 2 | -41.7 |
| 309 | Ametoctradin | 15.58 | + | 162 | 276.2 >149.0 (38) | 276.2 >176.0 (38) | 1-200 | 0.9983 | 90.0 (6.0) | 88.6 (4.8) | 90.2 (6) | 0.6 | 2 | 16.9 |
| 310 | Trifloxystrobin | 15.70 | + | 139 | 409.1 >186.0 (20) | 409.1 >145.0 (44) | 1-200 | 0.9998 | 91.4 (6.9) | 86 (5.8) | 87.3 (6.5) | 0.6 | 2 | -11 |
| 311 | Cycloxidim | 15.76 | + | 111 | 325.9 >180.0 (21) | 325.9 >280.1 (13) | 1-200 | 0.9991 | 90.2 (6.2) | 89.6 (4.5) | 92.5 (4.8) | 0.6 | 2 | 15.5 |
| 312 | Penthiopyrad | 15.79 | + | 146 | 360.1 >244.0 (23) | 360.1 >276.0 (15) | 1-200 | 0.9993 | 88.2 (4.3) | 88.7 (5.9) | 90.2 (6.6) | 0.6 | 2 | 19.8 |
| 313 | Flufenoxuron | 15.81 | + | 182 | 489.0 >158.0 (21) | 489.0 >141.0 (43) | 1-200 | 0.9987 | 108.0 (7.5) | 107.9 (5.4) | 109.6 (4.7) | 0.6 | 2 | -62.1 |
| 314 | Fenpyroximat | 15.99 | + | 151 | 422.1 >366.1 (15) | 422.1 >214.1 (24) | 1-200 | 0.9989 | 89.0 (4.9) | 89.7 (4.5) | 89.9 (6.6) | 0.6 | 2 | -45.6 |
| 315 | Spirodiclofen | 16.01 | + | 141 | 411.0 >313.0 (10) | 411.0 >71.37 (15) | 1-200 | 0.9998 | 89.0 (4.7) | 89.4 (5.7) | 88.2 (6.5) | 0.6 | 2 | -41.2 |
| 316 | Etoxazole | 16.02 | + | 167 | 360.1 >141.1 (27) | 360.1 >304.0 (19) | 1-200 | 0.9996 | 90.1 (5.9) | 90.6 (6.7) | 89.4 (5.9) | 0.6 | 2 | -34 |
| 317 | Isopyrazam | 16.14 | + | 146 | 360.1 >244.0 (23) | 360.1 >276.0 (15) | 2-200 | 0.9998 | 87.7 (6.2) | 88.3 (4.6) | 87.7 (5.3) | 1.5 | 5 | 19.8 |
| 318 | Triflumizole | 16.21 | + | 103 | 346.1 >278.1 (5) | 346.1 >73.1 (15) | 1-200 | 0.9994 | 87.4 (4.4) | 89.3 (5.1) | 88.1 (4.3) | 0.6 | 2 | -13.6 |
| 319 | Hexaflumuron | 16.36 | – | 105 | 458.9 >438.9 (14) | 458.9 >174.8 (37) | 1-200 | 0.9987 | 87.9 (5) | 89.5 (6) | 90.4 (5.6) | 0.6 | 2 | -25.1 |
| 320 | Chlorfluazuron | 16.37 | + | 149 | 539.9 >382.9 (23) | 539.9 >346.9 (46) | 1-200 | 0.9986 | 89.9 (5.9) | 88.7 (6.5) | 90.2 (4.9) | 0.6 | 2 | -71.1 |
| 321 | Spinetoram | 16.39 | + | 200 | 748.5 >142.2 (31) | 748.5 >98.1 (40) | 1-200 | 0.9987 | 90.5 (7) | 89.2 (6.1) | 90.9 (5.8) | 0.6 | 2 | 5.4 |
| 322 | Avermectin (B1b) (*) | 16.42 | + | 76 | 876.5 >291.0 (21) | 876.5 >553.4 (13) | 1-200 | 0.9986 | 90.8 (5.7) | 88.9 (5.4) | 90.3 (4.1) | 0.6 | 2 | -6 |
| 323 | Fenoxaprop-P-ethyl | 16.51 | + | 166 | 363.2 >145.0 (24) | 363.2 >289.0 (18) | 2-200 | 0.9987 | 88.9 (6.3) | 85.1 (3.2) | 86.8 (5.5) | 1.5 | 5 | 11.9 |
| 324 | Pyridaben | 16.62 | + | 90 | 365.0 >309.1 (14) | 365.0 >147.1 (24) | 1-200 | 0.9994 | 109.5 (5.4) | 107.4 (4.7) | 109.4 (5.9) | 0.6 | 2 | -60.5 |
| 325 | Novaluron | 16.66 | – | 119 | 490.9 >470.9 (14) | 490.9 >304.9 (19) | 1-200 | 0.9981 | 89.6 (7.2) | 89.2 (5.2) | 90.5 (6) | 0.6 | 2 | -35.5 |
| 326 | Furathiocarb | 16.71 | + | 133 | 383.2 >195.0 (21) | 383.2 >167.0 (27) | 1-200 | 0.9995 | 93.3 (6.2) | 88.7 (4.9) | 89.9 (4.4) | 0.6 | 2 | -9.9 |
| 327 | Cyflumetofen | 16.74 | + | 132 | 448.3 >172.9 (19) | 448.3 >248.7 (7) | 2-200 | 0.9996 | 88.2 (5.8) | 90.1 (4.6) | 87.3 (5.8) | 1.5 | 5 | -16.8 |
| 328 | Esprocarb | 16.80 | + | 115 | 266.2 >91.1 (25) | 266.2 >196.1 (15) | 1-200 | 0.9997 | 90.4 (5.5) | 88.3 (6.3) | 88.4 (6.4) | 0.6 | 2 | 11.7 |

| | | | | | | | | | | | | | | |
|---|---|---|---|---|---|---|---|---|---|---|---|---|---|---|
| 329 | Fenazaquin | 16.83 | + | 161 | 307.2 >161.1 (19) | 307.2 >57.44 (25) | 1-200 | 0.9983 | 90.9 (5.8) | 91.7 (5.2) | 90.8 (5.7) | 0.6 | 2 | -50.1 |
| 330 | Avermectin (B1a) (*) | 16.93 | + | 77 | 890.4 >305.1 (24) | 890.4 >567.2 (14) | 1-200 | 0.9997 | 88.1 (3.8) | 88.5 (6) | 90.5 (5.8) | 0.6 | 2 | -6 |
| 331 | Eprinomectin | 17.10 | + | 85 | 914.5 >186.1 (27) | 914.5 >154.0 (27) | 1-200 | 0.9982 | 90.6 (4.8) | 90.1 (5.9) | 88.4 (5.3) | 0.6 | 2 | 1.5 |
| 332 | Butachlor | 17.23 | + | 115 | 312.2 >262.2 (22) | 312.2 >238.1 (13) | 1-200 | 0.9985 | 88.8 (7.3) | 86.8 (5) | 90.4 (5) | 0.6 | 2 | 4.5 |
| 333 | Doramectin | 17.64 | + | 78 | 916.5 >331.2 (22) | 916.5 >593.3 (12) | 1-200 | 0.9988 | 88.4 (4.9) | 89.6 (5.9) | 87.9 (5.7) | 0.6 | 2 | -11.9 |
| 334 | Moxidectin | 17.96 | + | 68 | 640.3 >528.2 (10) | 640.3 >498.2 (10) | 2-200 | 0.9994 | 109.3 (6.4) | 109.6 (6.7) | 106.1 (6) | 1.5 | 5 | -68.6 |
| 335 | Etaconazole (*) | 10.24, 10.52 | + | 172 | 328.1 >159.2 (23) | 328.1 >205.1 (18) | 1-200 | 0.9996 | 89.2 (5.3) | 88.9 (5.1) | 91 (3.9) | 0.6 | 2 | 18.9 |
| 336 | Difenoconazole (*) | 12.94, 13.15 | + | 188 | 406.1 >251.0 (26) | 406.1 >253.1 (26) | 1-200 | 0.9987 | 92.9 (4.5) | 90.8 (6.4) | 91.2 (6.4) | 0.6 | 2 | -4.3 |
| 337 | Ipconazole (*) | 13.1, 13.52 | + | 166 | 334.2 >70.2 (27) | 334.2 >125.1 (33) | 1-200 | 0.9983 | 88.7 (5.1) | 89.4 (5.9) | 88.9 (4.3) | 0.6 | 2 | -8.9 |
| 338 | Mevinphos (*) | 3.12, 3.75 | + | 88 | 225.1 >127.0 (19) | 225.1 >109.1 (34) | 1-200 | 0.9988 | 89.1 (5.6) | 87 (4.7) | 90.4 (5.8) | 0.6 | 2 | -15.3 |
| 339 | Siduron (*) | 8.26, 9.11 | + | 101 | 233.1 >94.15 (18) | 233.1 >137.0 (15) | 1-200 | 0.9983 | 89.1 (5.4) | 88.8 (5.3) | 88.9 (5) | 0.6 | 2 | 17.7 |
| 340 | Spiroxamine (*) | 9.10, 9.26 | + | 142 | 298.3 >144.1 (20) | 298.3 >100.2 (30) | 1-200 | 0.9984 | 89.1 (5.7) | 90.3 (5.8) | 90.5 (4.8) | 0.6 | 2 | 15.8 |
| 341 | Bromucanozole (*) | 9.5, 10.65 | + | 178 | 377.9 >159.0 (30) | 377.9 >161.0 (31) | 1-200 | 0.998 | 90.1 (4.5) | 88.7 (6.3) | 89.1 (6.6) | 0.6 | 2 | 16.3 |

(*) Pesticides with two isomers, calculated with 2 peaks together.

**Table S3.** PTV injection conditions.

| Parameters | Value |
|---|---|
| Injection time (min) | 0.1 |
| Injection volume (μL) | 3.0 |
| Transfer rate (°C/s) | 2.5 |
| Transfer temperature (°C) | 300 |
| Transfer time (min) | 3 |
| Cleaning rate (°C) | 14.5 |
| Cleaning temperature (°C) | 320 |
| Cleaning time (min) | 20 |
| Cleaning flow (mL/min) | 50 |
| Cleaning phase | Post cycle temperature cool down |

**Table S4.** List of pesticides, method parameters and results of validation parameters: linearity range, matrix effect, limit of detection, limit of quantitation, recovery and reproducibility of GC-MS/MS method.

| | Compound | RT (min) | Quantitative peak (collision energy) | Confirm peak (collision energy) | Linear range | $R^2$ | % Recovery (% $RSD_R$, n = 12) | | | LOD (μg/kg) | LOQ (μg/kg) | Matrix effect (%) |
|---|---|---|---|---|---|---|---|---|---|---|---|---|
| | | | | | | | 10 μg/kg | 50 μg/kg | 100 μg/kg | | | |
| 1 | 4-Chloronitrobenzene | 7.74 | 157 > 100 (14) | 157 > 111 (10) | 5-100 | 0.9966 | 89.5 (6.1) | 97.7 (7.4) | 98 (7.2) | 1.5 | 5 | -15.6 |
| 2 | Dichlorvos | 7.85 | 109 > 79 (6) | 185 > 93 (12) | 1-100 | 0.9989 | 91.2 (4.1) | 97.4 (7.5) | 95.1 (7) | 0.3 | 1 | 10.2 |
| 3 | Allidochlor | 8.30 | 132 > 56 (6) | 138 > 96 (8) | 5-100 | 0.9968 | 71.4 (7.1) | 118.7 (4.9) | 70.7 (6.1) | 1.5 | 5 | 6.01 |
| 4 | EPTC | 8.44 | 128 > 43 (10) | 189 > 128 (6) | 1-100 | 0.9993 | 90.9 (6) | 96.3 (7.5) | 93.7 (9.9) | 0.3 | 1 | 11.5 |
| 5 | 3-Chloro-4-Methylaniline | 8.88 | 141 > 106 (14) | 141 > 93 (19) | 1-100 | 0.9987 | 87.5 (6.7) | 99.8 (6.5) | 98.3 (8.1) | 0.3 | 1 | 6.3 |
| 6 | Diclobenil | 9.23 | 171 > 100 (25) | 171 > 136 (13) | 5-100 | 0.9974 | 87.3 (7) | 75.4 (8.4) | 92.9 (10.7) | 1.5 | 5 | 22 |
| 7 | Indanofan | 9.28 | 174 > 131 (20) | 174 > 159 (20) | 10-100 | 0.9962 | 88.8 (4.7) | 95.3 (6.7) | 93.5 (10.6) | 3 | 10 | 1.2 |

| | | | | | | | | | | | |
|---|---|---|---|---|---|---|---|---|---|---|---|
| 8 | Chlorbromuron | 9.66 | 232 > 124 (20) | 124 > 97 (8) | 10-100 | 0.9989 | 87.6 (3.7) | 100.4 (7.9) | 91.3 (6.1) | 3 | 10 | 10.6 |
| 9 | Dichlormid | 9.81 | 172 > 108 (6) | 172 > 136 (6) | 1-100 | 0.9982 | 88.6 (6.6) | 96.5 (7.7) | 94.6 (9) | 0.3 | 1 | -13.4 |
| 10 | Dichlobenil | 9.82 | 170 > 99 (24) | 170 > 136 (14) | 1-100 | 0.9966 | 90.9 (5) | 100.7 (8.7) | 96.1 (11) | 0.3 | 1 | -11.5 |
| 11 | Biphenyl | 9.95 | 154 > 115 (26) | 154 > 127 (30) | 5-100 | 0.9974 | 90.5 (6.1) | 95.9 (7.7) | 95.8 (7.2) | 1.5 | 5 | 22 |
| 12 | Mevinphos | 10.43 | 127 > 109 (8) | 192 > 127 (10) | 5-100 | 0.994 | 70.2 (5) | 99 (7.4) | 97.2 (7.3) | 1.5 | 5 | 13.3 |
| 13 | Dichloroaniline, 3,4'- | 10.62 | 161 > 99 (21) | 161 > 126 (8) | 2-100 | 0.9953 | 89.6 (5.7) | 71.1 (5.9) | 97.2 (9.8) | 0.6 | 2 | -22.4 |
| 14 | Nitrapyrin | 10.85 | 231 > 194 (21) | 231 > 198 (20) | 5-100 | 0.9974 | 90 (6.1) | 97.1 (6.7) | 97.1 (5) | 1.5 | 5 | 15.4 |
| 15 | Etridiazole | 10.88 | 183 > 140 (16) | 211 > 183 (10) | 5-100 | 0.9978 | 70.2 (5.7) | 95 (6.7) | 92.2 (9.6) | 1.5 | 5 | 78.6 |
| 16 | Acenaphthene | 10.94 | 154 > 153 (16) | 154 > 152 (24) | 5-100 | 0.9982 | 89.8 (5.4) | 98 (7.6) | 99.7 (9.9) | 1.5 | 5 | 10.7 |
| 17 | Pebulate | 11.02 | 128 > 57 (8) | 161 > 128 (6) | 10-100 | 0.9956 | 70.1 (6.1) | 125.1 (3.8) | 69.9 (6) | 3 | 10 | 62.6 |
| 18 | N-(2,4-Dimethylphenyl) formamide | 11.32 | 120 >77 (15) | 121 >106 (8) | 5-100 | 0.9954 | 69.5 (5.4) | 97 (6.3) | 92.7 (10.8) | 1.5 | 5 | 10.9 |
| 19 | 3,5-Dichloroaniline | 11.51 | 161 > 98 (22) | 161 > 90 (20) | 1-100 | 0.9988 | 88.5 (6.2) | 100.5 (5.2) | 95.5 (6.9) | 0.3 | 1 | 11.7 |
| 20 | Dimethyl phthalate | 11.51 | 163 > 77 (20) | 163 > 133 (8) | 1-100 | 0.9974 | 90.5 (5.6) | 98.7 (7.3) | 92 (8.7) | 0.3 | 1 | 16.9 |
| 21 | Tetrahydrophthalimide, trans- | 11.51 | 151 > 80 (6) | 151 > 122 (10) | 5-100 | 0.9966 | 90.8 (5.4) | 101.4 (5) | 93.5 (8) | 1.5 | 5 | -17.8 |
| 22 | Methacrifos | 11.53 | 125 > 79 (6) | 180 > 93 (8) | 5-100 | 0.9969 | 68.9 (5.1) | 100.6 (8) | 95.2 (9) | 1.5 | 5 | 50.5 |
| 23 | Clopyralid | 11.54 | 192 > 117 (11) | 192 > 147 (9) | 10-100 | 0.9994 | 93 (4.5) | 98.3 (7.4) | 93.4 (10.7) | 3 | 10 | 16.9 |
| 24 | Thiocyclam | 11.56 | 181 > 88 (24) | 181 > 135 (11) | 10-100 | 0.9996 | 88.9 (3.4) | 100 (8.1) | 93.4 (9) | 3 | 10 | 12.7 |
| 25 | Chloroneb | 11.68 | 191 > 113 (15) | 193 > 115 (15) | 5-100 | 0.9977 | 89.5 (5.2) | 121.7 (5.1) | 93.3 (9.4) | 1.5 | 5 | 35.2 |
| 26 | 2-Phenylphenol | 11.94 | 141 > 115 (12) | 170 > 141 (22) | 5-100 | 0.9968 | 89.8 (5.2) | 100.2 (8.8) | 93.3 (7) | 1.5 | 5 | 13.8 |
| 27 | 4-Chlorophenoxyacetic acid | 11.94 | 186 > 143 (12) | 186 > 111 (20) | 10-100 | 0.998 | 90.2 (6) | 95.4 (6.1) | 96.4 (11.1) | 3 | 10 | -18.9 |
| 28 | Pentachlorobenzene | 11.96 | 250 > 179 (30) | 250 > 215 (20) | 1-100 | 0.9956 | 108.8 (13.6) | 96.7 (6.8) | 98 (9.2) | 0.3 | 1 | 54.6 |
| 29 | Dibutyl succinate | 12.27 | 230 > 101 (23) | 230 > 157 (13) | 5-100 | 0.9963 | 88.5 (5.2) | 101.6 (8.1) | 100.6 (7) | 1.5 | 5 | 16.6 |
| 30 | Mecoprop Methyl Ester | 12.44 | 142 > 107 (8) | 228 > 169 (8) | 5-100 | 0.9998 | 91.1 (6.7) | 102.8 (5.2) | 101.3 (8.8) | 1.5 | 5 | 17.2 |
| 31 | tert-butyl-4-Hydroxyanisole | 12.52 | 180 > 165 (8) | 180 > 137 (14) | 1-100 | 0.9971 | 93.8 (4.3) | 97.5 (6.4) | 98.3 (9.3) | 0.3 | 1 | 19.3 |
| 32 | Tecnazene | 12.96 | 215 > 179 (8) | 261 > 203 (13) | 5-100 | 0.9983 | 89.1 (6) | 96.6 (6.8) | 95.2 (9.4) | 1.5 | 5 | 72.4 |
| 33 | Propachlor | 13.15 | 120 >77 (19) | 176 >57 (8) | 5-100 | 0.9993 | 70 (6.1) | 92.5 (5.4) | 96 (8.9) | 1.5 | 5 | 18.9 |
| 34 | Chlorethoxyfos | 13.30 | 229 > 172 (16) | 215 > 134 (10) | 1-100 | 0.9964 | 91.6 (6.2) | 95.1 (8) | 96 (9.3) | 0.3 | 1 | 12.3 |
| 35 | Diphenylamine | 13.45 | 168 > 167 (14) | 168 > 139 (38) | 5-100 | 0.9955 | 70.1 (6.8) | 100.6 (5.5) | 95.1 (9.4) | 1.5 | 5 | 45.3 |
| 36 | Tetrachloroaniline, 2,3,5,6- | 13.47 | 231 > 158 (20) | 231 > 160 (22) | 5-100 | 0.9981 | 66.9 (5.9) | 120.7 (6.2) | 92.5 (9.8) | 1.5 | 5 | 53.8 |
| 37 | Imibenconazole | 13.51 | 409 > 250 (1) | 411 > 253 (1) | 1-100 | 0.9988 | 89.1 (5.2) | 96 (7.4) | 96.6 (9.4) | 0.3 | 1 | 14.9 |
| 38 | Cycloate | 13.59 | 154 > 83 (8) | 215 > 154 (6) | 5-100 | 0.9982 | 73.2 (5.9) | 96.2 (6.4) | 95.6 (8) | 1.5 | 5 | 60.5 |

| | | | | | | | | | | | | |
|---|---|---|---|---|---|---|---|---|---|---|---|---|
| 39 | Ethalfluralin | 13.64 | 276 > 202 (15) | 316 > 276 (10) | 5-100 | 0.9935 | 90.6 (6.1) | 98.6 (6.2) | 94.5 (9.1) | 1.5 | 5 | 53.6 |
| 40 | Trifluralin-d14 (**) | 13.70 | 315 > 163 (25) | 315 > 267 (10) | - | - | - | - | - | - | - | - |
| 41 | Tolylsulfonylbutylurea | 13.75 | 270 > 197 (20) | 270 > 91 (15) | 5-100 | 0.9945 | 89.1 (6.3) | 87.4 (5.1) | 90.3 (7.1) | 1.5 | 5 | 17.4 |
| 42 | Chlorfenprop-Methyl | 13.79 | 195 > 165 (10) | 165 > 137 (10) | 1-100 | 0.9987 | 88.1 (5.2) | 98.2 (7.7) | 97.2 (8.2) | 0.3 | 1 | 12.4 |
| 43 | Chlorpropham | 13.81 | 171 > 127 (8) | 213 > 171 (8) | 5-100 | 0.9964 | 93.2 (6.5) | 95.9 (5.7) | 92.1 (9.3) | 1.5 | 5 | 6.54 |
| 44 | Heptenophos | 13.82 | 124 > 89 (12) | 124 > 62 (28) | 5-100 | 0.9952 | 90.7 (6.1) | 94.9 (5.1) | 99.4 (8.5) | 1.5 | 5 | 10.8 |
| 45 | Trifluralin | 13.85 | 306 > 206 (12) | 306 > 264 (8) | 2-100 | 0.9935 | 91.6 (5.9) | 97.7 (8.1) | 96.2 (9.4) | 0.6 | 2 | 52.4 |
| 46 | Benfluralin | 13.92 | 292 > 206 (12) | 292 > 264 (8) | 2-100 | 0.9956 | 87.6 (6) | 97.6 (6.2) | 99.2 (8.6) | 0.6 | 2 | 40.1 |
| 47 | 1,2,3,6-Tetrahydrophthalimide,cis- | 13.93 | 151 > 79 (6) | 151 > 77 (30) | 5-100 | 0.9987 | 91.3 (7.1) | 98.6 (7.2) | 96.6 (8.4) | 1.5 | 5 | 17.8 |
| 48 | Dichlorprop-methyl | 14.01 | 162 > 63 (26) | 162 > 98 (16) | 2-100 | 0.9997 | 88.4 (5.4) | 96.3 (5.8) | 94.7 (9.4) | 0.6 | 2 | 16.9 |
| 49 | Sulfotep | 14.02 | 202 > 146 (10) | 322 > 202 (10) | 5-100 | 0.9997 | 89.3 (5.3) | 96.3 (7.4) | 93 (10.9) | 1.5 | 5 | 20.7 |
| 50 | Furmecyclox | 14.17 | 123 > 43 (16) | 123 > 53 (12) | 5-100 | 0.9994 | 91.2 (5.1) | 97.6 (7.4) | 98.6 (9.1) | 1.5 | 5 | -13.4 |
| 51 | Phorate | 14.34 | 121 >65 (10) | 260 >75 (8) | 5-100 | 0.9964 | 70.2 (6.5) | 96.1 (8.2) | 95.6 (8.6) | 1.5 | 5 | 102.2 |
| 52 | Lindan-d6 (**) | 14.39 | 224 > 150 (20) | 224 > 187 (10) | 2-100 | 0.9964 | 91.1 (7.2) | 96.4 (8) | 91.7 (6.8) | 0.6 | 2 | 16.7 |
| 53 | Captan | 14.42 | 264 > 79 (19) | 264 > 105 (10) | 2-100 | 0.9982 | 89.1 (5.1) | 85.1 (6.7) | 91.4 (5.3) | 1.5 | 5 | -15.3 |
| 54 | BHC, alpha- | 14.49 | 181 > 145 (13) | 219 > 183 (8) | 5-100 | 0.9954 | 72.9 (4.4) | 97.6 (7) | 93.1 (8.4) | 1.5 | 5 | 38.6 |
| 55 | Hexachlorobenzene | 14.59 | 249 > 214 (14) | 284 > 249 (18) | 5-100 | 0.9952 | 88.5 (7.1) | 121.3 (5.6) | 97.9 (8.1) | 1.5 | 5 | 48.7 |
| 56 | Pentachloroanisole | 14.73 | 265 > 237 (12) | 267 > 239 (10) | 5-100 | 0.9969 | 90.8 (6.2) | 96.4 (6.6) | 94.7 (8.5) | 1.5 | 5 | 34.9 |
| 57 | Dicloran | 14.79 | 176 > 148 (10) | 206 > 176 (10) | 1-100 | 0.9961 | 91.1 (4.8) | 96 (6.6) | 93.7 (10) | 0.3 | 1 | 31.8 |
| 58 | BHC, beta- | 15.10 | 181 > 145 (15) | 219 > 183 (8) | 2-100 | 0.9984 | 89.5 (7.5) | 96.1 (6.8) | 95.6 (8) | 0.6 | 2 | -8.5 |
| 59 | Clomazone | 15.17 | 125 > 89 (13) | 125 > 99 (17) | 5-100 | 0.9983 | 88.8 (6.4) | 98.4 (6) | 99.7 (6.2) | 1.5 | 5 | 13.7 |
| 60 | Quintozene | 15.21 | 235 > 141 (26) | 295 > 237 (15) | 5-100 | 0.9952 | 67.7 (5.8) | 117.9 (5.5) | 96.5 (8.4) | 1.5 | 5 | 76.3 |
| 61 | Profluralin | 15.25 | 318 > 199 (17) | 330 > 69 (25) | 5-100 | 0.9971 | 71.2 (7.3) | 95.6 (7.8) | 91.5 (8.2) | 1.5 | 5 | 28.6 |
| 62 | Pentachlorobenzonitrile | 15.30 | 273 > 238 (17) | 275 > 205 (30) | 5-100 | 0.9954 | 70.1 (5.5) | 121.1 (5.4) | 95.8 (7.6) | 1.5 | 5 | 32.3 |
| 63 | BHC, gamma- | 15.34 | 181 > 145 (13) | 219 > 183 (8) | 5-100 | 0.9962 | 68.1 (5.5) | 95.8 (6.5) | 92.4 (7.3) | 1.5 | 5 | 22.3 |
| 64 | Terbutylazine | 15.40 | 229 > 138 (12) | 229 > 173 (8) | 5-100 | 0.9953 | 88.5 (5.3) | 93.8 (8.6) | 94.6 (10.1) | 1.5 | 5 | 5.1 |
| 65 | Terbufos | 15.42 | 231 > 129 (23) | 231 > 175 (12) | 5-100 | 0.9983 | 71.5 (5.9) | 96.1 (7.2) | 91.5 (8.2) | 1.5 | 5 | 102.3 |
| 66 | Propyzamide | 15.49 | 173 > 109 (27) | 173 > 145 (13) | 5-100 | 0.9971 | 89.8 (6.4) | 100.2 (7.7) | 97.6 (8.4) | 1.5 | 5 | 16.1 |
| 67 | Diazinon | 15.52 | 137 > 84 (12) | 304 > 179 (10) | 5-100 | 0.9953 | 71.1 (5.4) | 100.9 (7.2) | 92.8 (7.5) | 1.5 | 5 | 10.2 |
| 68 | Fonofos | 15.53 | 137 > 109 (6) | 246 > 137 (8) | 5-100 | 0.9981 | 69.9 (5.1) | 98.5 (7.1) | 91.5 (8.9) | 1.5 | 5 | 72.3 |
| 69 | Fluchloralin | 15.55 | 264 > 160 (15) | 306 > 264 (8) | 1-100 | 0.9973 | 86.8 (6) | 96.3 (9.1) | 95.4 (6.8) | 0.3 | 1 | 13.9 |

| | | | | | | | | | | | | |
|---|---|---|---|---|---|---|---|---|---|---|---|---|
| 70 | Pyrimethanil | 15.68 | 198 > 118 (32) | 198 > 183 (16) | 5-100 | 0.9987 | 72.1 (3.6) | 98.3 (6.1) | 90.2 (9.8) | 1.5 | 5 | 3.6 |
| 71 | Chlorothalonil | 15.72 | 264 > 168 (23) | 266 > 133 (40) | 5-100 | 0.9984 | 89.6 (7) | 100.6 (5.2) | 99.7 (10.6) | 1.5 | 5 | -33.2 |
| 72 | Terbacil | 15.83 | 160 > 117 (8) | 161 > 144 (14) | 5-100 | 0.9953 | 91.7 (6) | 93.9 (7.3) | 95.8 (7.2) | 1.5 | 5 | 25.5 |
| 73 | Isazofos | 15.84 | 161 > 119 (8) | 172 > 130 (8) | 5-100 | 0.9984 | 68.4 (6.9) | 99.2 (6.8) | 96.6 (9) | 1.5 | 5 | 8.9 |
| 74 | Disulfoton | 15.85 | 88 > 60 (6) | 142 > 109 (6) | 5-100 | 0.9993 | 93.8 (4.6) | 95 (6.5) | 71.7 (5.6) | 1.5 | 5 | 134.6 |
| 75 | Tefluthrin | 15.86 | 177 > 127 (15) | 177 > 137 (15) | 5-100 | 0.9978 | 86.8 (6.1) | 98.8 (8.7) | 91.3 (9.8) | 1.5 | 5 | 9.8 |
| 76 | 1-Naphthylacetic acid | 15.92 | 186 > 141 (17) | 186 > 115 (19) | 2-100 | 0.9992 | 92.5 (5.3) | 100.4 (6.3) | 96.9 (9.6) | 0.6 | 2 | -12.4 |
| 77 | BHC, delta- | 16.03 | 181 > 145 (13) | 219 > 183 (8) | 1-100 | 0.9993 | 66.8 (6.5) | 92.9 (4.8) | 95.4 (9.3) | 0.3 | 1 | -19.1 |
| 78 | Triallate | 16.03 | 268 > 184 (20) | 268 > 226 (10) | 5-100 | 0.9956 | 88.2 (5.2) | 95.4 (8.1) | 93.4 (9.4) | 1.5 | 5 | 22.1 |
| 79 | Pentachloroaniline | 16.45 | 263 > 192 (20) | 265 > 194 (22) | 5-100 | 0.9974 | 88.3 (7) | 100 (7.3) | 94.7 (11.1) | 1.5 | 5 | -46.9 |
| 80 | Endosulfan ether | 16.51 | 239 > 204 (13) | 241 > 206 (13) | 5-100 | 0.9987 | 67.9 (5.8) | 96 (6.6) | 97 (8.4) | 1.5 | 5 | -46.7 |
| 81 | Dioxabenzofos | 16.56 | 183 > 153 (8) | 216 > 138 (8) | 1-100 | 0.9991 | 90.3 (5.8) | 98.9 (7.3) | 96.8 (8.8) | 0.3 | 1 | -17.7 |
| 82 | Fenoprop | 16.58 | 269 > 196 (8) | 269 > 97 (24) | 10-100 | 0.9989 | 89.5 (6.1) | 95.3 (7) | 94.4 (9.5) | 3 | 10 | -9.1 |
| 83 | Propanil | 16.61 | 217 > 161 (8) | 219 > 163 (8) | 1-100 | 0.9983 | 90.3 (6.3) | 97.9 (7.7) | 93.5 (9.6) | 0.3 | 1 | 11.2 |
| 84 | Dimethachlor | 16.65 | 134 > 105 (23) | 197 > 148 (8) | 5-100 | 0.9963 | 89.7 (7.3) | 99.5 (6.7) | 96 (9) | 1.5 | 5 | -60.4 |
| 85 | Chlorpyrifos-methyl | 16.73 | 286 > 93 (24) | 286 > 271 (12) | 5-100 | 0.9997 | 68.3 (4.2) | 95.6 (6.3) | 97.2 (8.9) | 1.5 | 5 | -3.2 |
| 86 | Acetochlor | 16.75 | 174 > 146 (12) | 223 > 132 (20) | 5-100 | 0.9964 | 91.4 (4.8) | 99.4 (8) | 93.7 (7.3) | 1.5 | 5 | -13.6 |
| 87 | Vinclozolin | 16.87 | 212 > 172 (12) | 285 > 212 (12) | 5-100 | 0.9956 | 87.8 (6.1) | 98 (7.5) | 95.4 (7.9) | 1.5 | 5 | -4.18 |
| 88 | Methyl parathion | 16.89 | 263 > 109 (10) | 263 > 136 (8) | 1-100 | 0.9969 | 89.9 (6.1) | 97.1 (8) | 93.8 (8.7) | 0.3 | 1 | 41.7 |
| 89 | Alachlor | 16.91 | 188 > 130 (32) | 188 > 160 (10) | 5-100 | 0.9994 | 89.8 (5.8) | 96 (8) | 89.3 (9.1) | 1.5 | 5 | -9.4 |
| 90 | Tolclofos-methyl | 16.93 | 265 > 250 (10) | 267 > 252 (10) | 5-100 | 0.9984 | 92.4 (5.6) | 98.2 (8.6) | 96.4 (8.7) | 1.5 | 5 | -4.6 |
| 91 | Transfluthrin | 16.94 | 163 > 91 (12) | 163 > 143 (13 | 5-100 | 0.9969 | 90.5 (4.5) | 95.1 (6.6) | 91.5 (10.9) | 1.5 | 5 | -3.4 |
| 92 | Propisochlor | 17.01 | 162 > 120 (13) | 162 > 144 (10) | 5-100 | 0.9962 | 91.2 (7.1) | 96 (5.3) | 95.1 (9.7) | 1.5 | 5 | -10.3 |
| 93 | Desethylterbuthylazine | 17.07 | 201 > 186 (8) | 201 > 68 (16) | 1-100 | 0.9986 | 89.5 (6) | 96.8 (8.8) | 94.9 (9.7) | 0.3 | 1 | -12.6 |
| 94 | Metalaxyl-M | 17.11 | 160 > 130 (10) | 220 > 160 (16) | 10-100 | 0.9993 | 90.1 (4.6) | 97.7 (5.8) | 95.5 (9.3) | 3 | 10 | -15.3 |
| 95 | Fenchlorphos | 17.16 | 285 > 270 (11) | 287 > 272 (11) | 5-100 | 0.9978 | 89.4 (6.2) | 100.1 (7.1) | 93.4 (6.3) | 1.5 | 5 | 6.9 |
| 96 | Heptachlor | 17.16 | 100 >65 (12) | 272 >237 (13) | 5-100 | 0.9964 | 88 (5.1) | 97.7 (6.5) | 97 (7.7) | 1.5 | 5 | 36.8 |
| 97 | Pirimiphos-methyl | 17.39 | 290 > 233 (10) | 305 > 180 ( (8) | 5-100 | 0.9982 | 73.2 (5.6) | 99.1 (6.6) | 91 (8.8) | 1.5 | 5 | -4.6 |
| 98 | Prodiamine | 17.41 | 279 > 203 (8) | 321 > 279 (6) | 1-100 | 0.9977 | 86.5 (6) | 100.5 (4.9) | 92.4 (7.6) | 0.3 | 1 | 30.9 |
| 99 | Fenitrothion | 17.48 | 277 > 109 (18) | 277 > 260 (6) | 5-100 | 0.9964 | 67.9 (6) | 97 (7.2) | 99.8 (8.3) | 1.5 | 5 | 43.6 |
| 100 | Tebupirimfos | 17.61 | 261 > 137 (15) | 233 > 110 (10) | 1-100 | 0.9968 | 90.1 (6.4) | 96.3 (8.5) | 94.9 (10.2) | 0.3 | 1 | 10.6 |

| | | | | | | | | | | | | |
|---|---|---|---|---|---|---|---|---|---|---|---|---|
| 101 | Linuron | 17.62 | 187 > 124 (21) | 248 > 61 (8) | 10-100 | 0.9989 | 90.7 (6.4) | 98.4 (8.2) | 89.1 (7.4) | 3 | 10 | -9.3 |
| 102 | Pentachlorothioanisole | 17.65 | 296 > 263 (12) | 298 > 265 (8) | 5-100 | 0.9978 | 75.9 (3.2) | 101.3 (7.5) | 89.6 (7.7) | 1.5 | 5 | 2 |
| 103 | Dichlofluanid | 17.67 | 123 > 77 (16) | 224 > 123 (12) | 10-100 | 0.9983 | 108 (13.6) | 97.9 (7.6) | 93.5 (9.4) | 3 | 10 | -14.9 |
| 104 | Malathion | 17.67 | 127 > 99 (6) | 173 > 127 (6) | 5-100 | 0.9988 | 69.2 (5.5) | 96.7 (7.6) | 93.2 (7.5) | 1.5 | 5 | 23.5 |
| 105 | Trietazine | 17.69 | 229 > 200 (10) | 200 > 68 (22) | 1-100 | 0.9981 | 88.3 (4.6) | 94.1 (8) | 93.9 (9.8) | 0.3 | 1 | 10.4 |
| 106 | Lindane | 17.71 | 290 > 219 (10) | 290 > 181 (15) | 1-100 | 0.9988 | 90.1 (4.8) | 100.6 (6.8) | 94.2 (8) | 0.3 | 1 | -12.4 |
| 107 | Dioxathion | 17.76 | 153 > 96 (10) | 125 > 97 (15) | 5-100 | 0.9967 | 90.3 (4.6) | 98.9 (8.2) | 95 (9) | 1.5 | 5 | -16.8 |
| 108 | Metolachlor | 17.82 | 238 > 133 (27) | 238 > 162 (10) | 2-100 | 0.9985 | 91 (6) | 96.1 (5.5) | 94.6 (8.7) | 0.6 | 2 | 5.7 |
| 109 | Chlorpyrifos | 17.85 | 197 > 169 (14) | 314 > 258 (12) | 5-100 | 0.9963 | 90.4 (7.3) | 99.1 (8.6) | 95.5 (9.8) | 1.5 | 5 | -7.16 |
| 110 | Tolprocarb | 17.90 | 346 > 265 (15) | 285 > 163 (10) | 2-100 | 0.9953 | 92.1 (6.5) | 89.1 (5.4) | 84.4 (7.2) | 1.5 | 5 | -15.3 |
| 111 | Parathion-d10 (**) | 17.94 | 115 >83 (10) | 301 > 115 (10) | - | - | - | - | - | - | - | - |
| 112 | Chlorthal-dimethyl | 17.97 | 301 > 223 (24) | 332 > 301 (8) | 5-100 | 0.9983 | 88.6 (6) | 100.2 (8) | 96.3 (7.9) | 1.5 | 5 | -11.6 |
| 113 | Fenthion | 17.97 | 278 > 109 (18) | 278 > 169 (17) | 5-100 | 0.9971 | 72.3 (6.4) | 99.6 (6.6) | 91.7 (6.9) | 1.5 | 5 | 176.1 |
| 114 | Aldrin | 18.03 | 263 > 193 (32) | 263 > 191 (30) | 5-100 | 0.9984 | 69 (6.5) | 98.5 (9) | 94.1 (8.5) | 1.5 | 5 | 8 |
| 115 | Parathion | 18.04 | 109 >81 (8) | 139 >109 (8) | 5-100 | 0.9973 | 91.9 (7.7) | 100.4 (5.8) | 91.1 (8.5) | 1.5 | 5 | 90.1 |
| 116 | Anthraquinone | 18.07 | 180 > 152 (12) | 208 > 180 (10) | 5-100 | 0.9991 | #DIV/0! | 98.6 (7.4) | 72.1 (5.4) | 1.5 | 5 | 104.7 |
| 117 | Triadimefon | 18.12 | 208 > 111 (24) | 208 > 181 (10) | 2-100 | 0.9975 | 88.6 (7.1) | 99.5 (7.2) | 96.6 (9.1) | 0.6 | 2 | 18.4 |
| 118 | Dichlorobenzophenone, 4,4'- | 18.26 | 139 > 75 (27) | 139 > 111 (13) | 2-100 | 0.9955 | 87.8 (6.4) | 99.9 (7.7) | 93.6 (8.8) | 0.6 | 2 | 52.5 |
| 119 | Chlorbufam | 18.30 | 152 > 125 (10) | 223 > 127 (12) | 5-100 | 0.9964 | 93.2 (5.3) | 100.6 (6.1) | 96.7 (7.5) | 1.5 | 5 | -12.1 |
| 120 | Pirimiphos-ethyl | 18.31 | 318 > 166 (12) | 318 > 182 (8) | 5-100 | 0.9954 | 89.2 (5.3) | 94.8 (5.6) | 99.2 (6.2) | 1.5 | 5 | 13 |
| 121 | 3-Phenylphenol | 18.32 | 141 > 115 (14) | 170 > 141 (22) | 5-100 | 0.9995 | 87.7 (6.6) | 95 (6.5) | 96.7 (8.3) | 1.5 | 5 | -16.6 |
| 122 | Fenson | 18.39 | 141 > 77 (8) | 268 > 141 (8) | 5-100 | 0.9967 | 93 (5.7) | 101.7 (7.3) | 95.7 (8.9) | 1.5 | 5 | 13.9 |
| 123 | Bromophos methyl | 18.40 | 329 > 314 (13) | 331 > 316 (13) | 5-100 | 0.9959 | 88.9 (5.4) | 99.3 (8.4) | 92.4 (8.7) | 1.5 | 5 | 27.1 |
| 124 | Diphenamid | 18.40 | 239 > 72 (10) | 239 > 167 (8) | 2-100 | 0.9968 | 87.1 (4.6) | 96.1 (6.6) | 93.3 (9.4) | 0.6 | 2 | 7.7 |
| 125 | Dinoterb | 18.41 | 225 > 177 (8) | 225 > 103 (20) | 10-100 | 0.9998 | 88.7 (6.6) | 101.7 (6.7) | 96.7 (11.2) | 3 | 10 | 12.4 |
| 126 | Isopropalin | 18.46 | 280 > 180 (10) | 280 > 238 (8) | 1-100 | 0.9963 | 88.7 (7.9) | 100.7 (6.8) | 93.2 (8.2) | 0.3 | 1 | 57.3 |
| 127 | 2,6-Dichlorobenzamide | 18.47 | 172 > 145 (14) | 188 > 173 (8) | 10-100 | 0.9964 | 88.9 (5.6) | 97.2 (6.1) | 98.9 (4.7) | 3 | 10 | 12.3 |
| 128 | Pendimethalin | 18.63 | 252 > 162 (10) | 252 > 191 (8) | 5-100 | 0.9974 | 90.1 (7.9) | 97.3 (8.4) | 94.1 (9.1) | 1.5 | 5 | 60.4 |
| 129 | Cyprodinil | 18.67 | 224 > 208 (20) | 225 > 210 (12) | 5-100 | 0.9987 | 70.4 (5.2) | 97.3 (8.1) | 92.5 (9) | 1.5 | 5 | 2.6 |
| 130 | Fipronil | 18.67 | 367 > 213 (30) | 369 > 215 (30) | 2-100 | 0.9954 | 90.3 (4.7) | 99 (8.9) | 95.4 (7.6) | 0.6 | 2 | 55.5 |
| 131 | Desethylsebutylazine | 18.68 | 201 > 172 (13) | 201 > 43 (8) | 5-100 | 0.9993 | 94.1 (5.4) | 95.8 (6.2) | 96 (9) | 1.5 | 5 | 12.3 |
| 132 | Metazachlor | 18.71 | 133 > 117 (25) | 209 > 132 (15) | 5-100 | 0.9969 | 91 (5.3) | 96.3 (9.7) | 92.9 (7.7) | 1.5 | 5 | 7.9 |

| | | | | | | | | | | | | |
|---|---|---|---|---|---|---|---|---|---|---|---|---|
| 133 | Alloxydim-sodium | 18.73 | 323 > 161 (22) | 323 > 107 (28) | 5-100 | 0.9989 | 89 (5.6) | 95.6 (6.2) | 97 (7.8) | 1.5 | 5 | 8.9 |
| 134 | Isodrin | 18.74 | 193 > 123 (30) | 193 > 157 (19) | 5-100 | 0.9966 | 87.1 (5.6) | 94.2 (6.9) | 98.7 (10.5) | 1.5 | 5 | -2.93 |
| 135 | Chlozolinate | 18.79 | 186 > 145 (16) | 331 > 259 (8) | 5-100 | 0.9954 | 88.7 (6.9) | 97.4 (8.1) | 94.4 (9.6) | 1.5 | 5 | -14.6 |
| 136 | Penconazole | 18.80 | 248 > 157 (26) | 248 > 192 (16) | 5-100 | 0.9988 | 87.8 (5.7) | 94.3 (7.1) | 93.5 (8.6) | 1.5 | 5 | 17.1 |
| 137 | Dichlofenthion | 18.84 | 250 > 223 (8) | 222 > 205 (12) | 1-100 | 0.9974 | 88.9 (4.6) | 97.4 (6.9) | 97.9 (9.7) | 0.3 | 1 | 12.6 |
| 138 | Tolylfluanid | 18.87 | 137 > 91 (17) | 238 > 137 (10) | 10-100 | 0.9972 | 88.3 (6) | 95.7 (9.4) | 95.3 (10.3) | 3 | 10 | 10.9 |
| 139 | Bromfenvinphos-methyl | 18.90 | 109 >79 (5) | 295 >109 (15) | 5-100 | 0.9985 | 74 (5) | 95.9 (5.9) | 95.2 (9.4) | 1.5 | 5 | 32.2 |
| 140 | Bioallethrin | 18.93 | 123 >81 (8) | 136 >93 (11) | 10-100 | 0.9951 | 68.9 (5.5) | 119.7 (6.2) | 97.7 (6.9) | 3 | 10 | 21.4 |
| 141 | Heptachlor epoxide | 18.94 | 351 > 261 (11) | 353 > 263 (13) | 5-100 | 0.9964 | 90 (5) | 96.5 (6.2) | 91.7 (9.1) | 1.5 | 5 | -8.5 |
| 142 | Quinalphos | 19.03 | 146 > 91 (24) | 146 > 118 (10) | 5-100 | 0.9974 | 92.7 (6.3) | 102 (5.7) | 93.7 (8.5) | 1.5 | 5 | 40.3 |
| 143 | Procymidone | 19.11 | 283 > 67 (28) | 283 > 96 (10) | 5-100 | 0.9954 | 90.4 (6.9) | 102.2 (5.5) | 96.6 (9.9) | 1.5 | 5 | -8 |
| 144 | Triadimenol | 19.11 | 128 > 65 (22) | 168 > 70 (10) | 5-100 | 0.9966 | 87.2 (5) | 97.6 (7.7) | 97.3 (9.1) | 1.5 | 5 | 20.1 |
| 145 | Triflumizole | 19.11 | 206 > 179 (14) | 206 > 186 (10) | 5-100 | 0.9984 | 89.9 (4) | 98 (7.3) | 92.2 (8.9) | 1.5 | 5 | 13.1 |
| 146 | Folpet | 19.27 | 104 >76 (10) | 260 >130 (16) | 5-100 | 0.9962 | 88 (5.2) | 74.5 (7.5) | 70.9 (5.9) | 1.5 | 5 | 54 |
| 147 | Cyanophos | 19.31 | 243 > 109 (10) | 125 > 79 (6) | 1-100 | 0.9981 | 88.7 (5.6) | 98.7 (8.9) | 96.5 (7.5) | 0.3 | 1 | 12.4 |
| 148 | Bromophos-ethyl | 19.35 | 331 > 303 (8) | 359 > 303 (17) | 1-100 | 0.9989 | 89.1 (6.3) | 95.4 (7.8) | 100.5 (7.4) | 0.3 | 1 | 19.2 |
| 149 | Octhilinone | 19.39 | 101 > 53 (16) | 114 > 58 (20) | 1-100 | 0.9974 | 91.6 (6.7) | 94.3 (9.3) | 94.1 (10.5) | 0.3 | 1 | 10.2 |
| 150 | Fenchlorphos-Oxon | 19.42 | 305 > 109 (15) | 305 > 269 (10) | 5-100 | 0.9977 | 90.8 (5.3) | 95.2 (7.9) | 91.4 (9.1) | 1.5 | 5 | 14.3 |
| 151 | Chlordane, trans- | 19.49 | 272 > 237 (13) | 375 > 266 (18) | 2-100 | 0.9971 | 89.8 (6.6) | 98.6 (5.1) | 94.9 (9.7) | 0.6 | 2 | 7.4 |
| 152 | Tetrachlorvinphos | 19.49 | 331 > 109 (20) | 333 > 109 (17) | 5-100 | 0.9983 | 89.3 (6) | 98.5 (6.8) | 94.6 (11.9) | 1.5 | 5 | 53.3 |
| 153 | DDE, o,p'- | 19.50 | 246 > 176 (32) | 316 > 246 (15) | 5-100 | 0.9967 | 91.1 (5.7) | 99.5 (8.3) | 95.8 (8.1) | 1.5 | 5 | -8.61 |
| 154 | Paclobutrazol | 19.57 | 236 > 125 (12) | 236 > 167 (10) | 5-100 | 0.9994 | 88.1 (5.3) | 97.4 (8.6) | 93.6 (9.4) | 1.5 | 5 | 30.9 |
| 155 | Dinitramine | 19.58 | 260 > 241 (8) | 215 > 196 (8) | 5-100 | 0.9998 | 89.7 (5.3) | 97.7 (6.1) | 94.9 (9.9) | 1.5 | 5 | 12.4 |
| 156 | Sebuthylazine | 19.65 | 230 > 200 (6) | 230 > 214 (6) | 5-100 | 0.9992 | 90.6 (5.3) | 102.6 (4.2) | 95 (9.6) | 1.5 | 5 | 10.4 |
| 157 | Dimethenamid | 19.76 | 230 >154 (10) | 154 > 111 (10) | 5-100 | 0.9967 | 86.1 (6.2) | 98.9 (7.5) | 100.1 (8.5) | 1.5 | 5 | 14.7 |
| 158 | Chlordane, cis- | 19.79 | 375 > 266 (21) | 377 > 268 (19) | 2-100 | 0.9984 | 90.5 (5.6) | 95.7 (8.7) | 100.4 (4.1) | 0.6 | 2 | -2.6 |
| 159 | Bromfenvinphos | 19.80 | 267 > 159 (15) | 323 > 267 (10) | 5-100 | 0.9954 | 87.2 (5.1) | 97.2 (8.3) | 89.7 (7.3) | 1.5 | 5 | 50 |
| 160 | Endosulfan I | 19.81 | 195 > 159 (6) | 241 > 206 (10) | 5-100 | 0.9978 | 89.4 (5.8) | 100 (6.8) | 94.2 (10.7) | 1.5 | 5 | -14.2 |
| 161 | Butralin | 19.81 | 266>224 (9) | 226>218 (8) | 2-100 | 0.9997 | 92.2 (7.1) | 98.6 (8) | 96.2 (9.6) | 0.6 | 2 | 76.7 |
| 162 | Flutriafol | 19.84 | 123 >95 (12) | 219 > 123 (14) | 2-100 | 0.9964 | 90.6 (6) | 99 (5) | 96.7 (8.1) | 0.6 | 2 | 19.6 |
| 163 | Nonachlor, trans- | 19.85 | 409 > 300 (20) | 409 > 302 (22) | 5-100 | 0.9987 | 93 (5) | 97.4 (8.4) | 97.7 (6.6) | 1.5 | 5 | 12.4 |

| | | | | | | | | | | | | |
|---|---|---|---|---|---|---|---|---|---|---|---|---|
| 164 | Tridiphane | 19.88 | 320 > 187 (15) | 320 > 173 (17) | 5-100 | 0.9971 | 88.3 (5.9) | 100 (7.9) | 97.2 (8.9) | 1.5 | 5 | -6.5 |
| 165 | Desmetryn | 19.89 | 213 > 170 (15) | 213 > 58 (8) | 1-100 | 0.9988 | 91.8 (5.2) | 98.2 (7.6) | 94.4 (9) | 0.3 | 1 | -12.8 |
| 166 | Flutolanil | 19.89 | 173 > 145 (14) | 281 > 173 (10) | 5-100 | 0.9964 | 68.7 (4.6) | 98.1 (8.7) | 93.4 (9) | 1.5 | 5 | 41.5 |
| 167 | Fludioxonil | 19.96 | 154 > 127 (8) | 248 > 127 (30) | 5-100 | 0.9965 | 89.7 (5.4) | 100.7 (6.8) | 96.2 (9.5) | 1.5 | 5 | 88.4 |
| 168 | Chlorfenson | 20.00 | 175 > 111 (8) | 177 > 113 (8) | 5-100 | 0.996 | 91.1 (6.2) | 97.1 (7.4) | 94.9 (6.2) | 1.5 | 5 | 17.6 |
| 169 | Pretilachlor | 20.03 | 162 > 132 (18) | 262 > 202 (8) | 5-100 | 0.9964 | 70.3 (5.4) | 99.1 (5.5) | 101.6 (6.4) | 1.5 | 5 | 28.7 |
| 170 | Prothiofos | 20.03 | 267 > 239 (8) | 309 > 239 (15) | 5-100 | 0.9961 | 88.4 (5.3) | 98.9 (6.7) | 94.2 (9.9) | 1.5 | 5 | 13.3 |
| 171 | Kinoprene | 20.06 | 149 > 77 (15) | 221 > 109 (8) | 10-100 | 0.9988 | 91.4 (5.2) | 97.7 (8.3) | 98.4 (9.9) | 3 | 10 | 12.4 |
| 172 | Profenofos | 20.13 | 337 > 267 (13) | 339 > 269 (13) | 2-100 | 0.9994 | 91.5 (6.3) | 98 (5.4) | 92.6 (8.9) | 0.6 | 2 | 84.6 |
| 173 | Octachlorostyrene | 20.14 | 380 > 308 (8) | 380 > 141 (12) | 1-100 | 0.9958 | 93.8 (5.9) | 98.7 (5.6) | 91.8 (7.8) | 0.3 | 1 | -14.6 |
| 174 | Oxadiazon | 20.17 | 175 > 112 (13) | 258 > 175 (8) | 5-100 | 0.9994 | 88.7 (5.5) | 99.3 (6.3) | 91.7 (9.2) | 1.5 | 5 | -10.6 |
| 175 | Tricyclazole | 20.19 | 189 > 135 (18) | 189 > 162 (12) | 5-100 | 0.9984 | 88.3 (6.5) | 98.1 (6.8) | 95.9 (9.3) | 1.5 | 5 | 175.1 |
| 176 | DDE, p,p'- | 20.23 | 246 > 176 (28) | 316 > 246 (20) | 5-100 | 0.9955 | 67.6 (4) | 97.8 (9.7) | 91.7 (10.5) | 1.5 | 5 | -8.08 |
| 177 | Oxyfluorfen | 20.28 | 252 > 146 (33) | 300 > 223 (15) | 2-100 | 0.9963 | 89.7 (5.8) | 100.2 (8.3) | 93.7 (9.1) | 0.6 | 2 | 119.4 |
| 178 | Myclobutanil | 20.29 | 179 > 125 (14) | 179 > 152 (8) | 5-100 | 0.9959 | 90.6 (5.5) | 96.5 (5.2) | 93.6 (6.7) | 1.5 | 5 | 22.7 |
| 179 | Bupirimate | 20.32 | 273 > 108 (16) | 273 > 193 (8) | 5-100 | 0.9952 | 88.9 (7) | 95.5 (7.6) | 96.4 (10.8) | 1.5 | 5 | 16.8 |
| 180 | Flusilazole | 20.33 | 233 > 152 (14) | 233 > 165 (18) | 5-100 | 0.9965 | 91.6 (7) | 101.8 (5.8) | 94.8 (11) | 1.5 | 5 | 17.7 |
| 181 | Aramite | 20.40 | 175 > 64 (22) | 135 > 64 (14) | 5-100 | 0.9974 | 66.5 (4.7) | 97.5 (7.1) | 91.9 (9.1) | 1.5 | 5 | -11.6 |
| 182 | Dieldrin | 20.43 | 263 > 193 (34) | 277 > 241 (8) | 10-100 | 0.996 | 91.7 (6.4) | 99.7 (8.3) | 98.3 (6.6) | 3 | 10 | -4.8 |
| 183 | Chlorfenapyr | 20.54 | 137 > 102 (12) | 249 > 112 (24) | 5-100 | 0.9964 | 89.5 (5.8) | 99.3 (5.7) | 95.4 (8.3) | 1.5 | 5 | 27.2 |
| 184 | Chlorthiophos | 20.70 | 257 > 193 (16) | 257 > 239 (12) | 5-100 | 0.9957 | 91 (6) | 96.8 (7.1) | 98.5 (9.8) | 1.5 | 5 | 12.4 |
| 185 | Fluazifop-P-butyl | 20.71 | 383 > 268 (8) | 383 > 282 (12) | 5-100 | 0.9987 | 67.5 (5.3) | 100.4 (5.8) | 94.2 (7.5) | 1.5 | 5 | 62.1 |
| 186 | Ethylan | 20.80 | 223 > 167 (12) | 223 > 179 (22) | 5-100 | 0.9964 | 91.2 (6) | 98.1 (8.3) | 88.2 (6.9) | 1.5 | 5 | 17.2 |
| 187 | Nitrofen | 20.80 | 202 > 139 (20) | 283 > 202 (10) | 1-100 | 0.9965 | 88.8 (6.4) | 99.2 (7.3) | 93.2 (8.9) | 0.3 | 1 | 109.1 |
| 188 | Tiocarbazil | 20.86 | 100 > 57 (6) | 156 > 57 (10) | 5-100 | 0.9982 | 89.5 (4.8) | 96.7 (8.9) | 95.8 (12.3) | 1.5 | 5 | 10.2 |
| 189 | Endrin | 20.92 | 263 > 193 (30) | 279 > 243 (8) | 5-100 | 0.9958 | 88.6 (7) | 96.6 (9.2) | 95.4 (10.6) | 1.5 | 5 | 7.52 |
| 190 | Chlorthiophos | 20.93 | 297 > 269 (8) | 325 > 269 (14) | 5-100 | 0.9985 | 89.8 (6.6) | 100.6 (5.2) | 94.8 (7.1) | 1.5 | 5 | 11.2 |
| 191 | Chlorobenzilate | 20.97 | 139 > 111 (12) | 251 > 139 (14) | 5-100 | 0.9968 | 90.6 (5.2) | 96.5 (6.9) | 97.8 (8.9) | 1.5 | 5 | 28 |
| 192 | Dimethipin | 20.99 | 117 > 58 (6) | 117 > 42 (14) | 10-100 | 0.9987 | 87.7 (5.3) | 99.6 (6.5) | 91.8 (8.2) | 3 | 10 | 11.5 |
| 193 | Endosulfan II | 21.15 | 195 > 125 (23) | 195 > 159 (8) | 5-100 | 0.9954 | 89.2 (6.1) | 96.1 (6.1) | 93.9 (7.5) | 1.5 | 5 | 4.23 |
| 194 | Ethion | 21.15 | 153 > 97 (10) | 231 > 129 (24) | 5-100 | 0.9953 | 91 (5.6) | 100.8 (6.3) | 92.7 (9.5) | 1.5 | 5 | 66.1 |

| | | | | | | | | | | | | |
|---|---|---|---|---|---|---|---|---|---|---|---|---|
| 195 | Atraton | 21.19 | 196 > 75 (24) | 211 > 75 (22) | 5-100 | 0.9963 | 70.7 (5.9) | 98.3 (9.1) | 97.7 (9.5) | 1.5 | 5 | 3.8 |
| 196 | Dipropetryn | 21.22 | 255 > 138 (22) | 255 > 180 (16) | 1-100 | 0.9988 | 90 (5.3) | 99.8 (8.6) | 95.4 (8) | 0.3 | 1 | 14.5 |
| 197 | Nonachlor, cis- | 21.24 | 409 > 300 (23) | 409 > 302 (20) | 5-100 | 0.9984 | 91.3 (6.2) | 117.2 (4.6) | 92.5 (8.3) | 1.5 | 5 | 4.84 |
| 198 | DDT, o,p'- | 21.26 | 235 > 165 (21) | 237 > 165 (22) | 5-100 | 0.994 | 90.6 (5.3) | 100.7 (8.5) | 94.1 (10) | 1.5 | 5 | 14.5 |
| 199 | Simeconazole | 21.37 | 121 > 101 (12) | 121 > 74 (24) | 2-100 | 0.9997 | 91.6 (7.6) | 95.9 (7) | 90.6 (10) | 0.6 | 2 | 16.3 |
| 200 | Triazophos | 21.48 | 161 > 134 (8) | 257 > 162 (8) | 1-100 | 0.9952 | 86.8 (6.5) | 95.5 (7.2) | 95.5 (6.6) | 0.3 | 1 | 95.9 |
| 201 | Sulprofos | 21.53 | 322 > 156 (13) | 322 > 97 (24) | 5-100 | 0.9972 | 70.5 (6.1) | 93.6 (6.1) | 94.2 (8.7) | 1.5 | 5 | 189 |
| 202 | Carfentrazone ethyl | 21.62 | 330 > 310 (8) | 340 > 312 (10) | 5-100 | 0.9956 | 88.7 (4.7) | 100.8 (7.3) | 95.6 (7.7) | 1.5 | 5 | 72.9 |
| 203 | Thiazopyr | 21.72 | 327 > 277 (26) | 306 > 215 (26) | 5-100 | 0.9983 | 90.7 (6) | 96.9 (8.3) | 92.5 (6.7) | 1.5 | 5 | 11.4 |
| 204 | 4,4'-Methoxychlor olefin | 21.74 | 238 > 152 (35) | 238 > 195 (20) | 5-100 | 0.9958 | 69 (5.9) | 100.8 (6.5) | 95.2 (8.1) | 1.5 | 5 | 12.3 |
| 205 | Chlorbenside | 21.74 | 125 > 89 (17) | 125 > 62 (28) | 10-100 | 0.9976 | 70.2 (6.6) | 98.3 (6.3) | 94.2 (7.5) | 3 | 10 | -24.5 |
| 206 | Norflurazon | 21.77 | 145 > 95 (18) | 303 > 145 (17) | 2-100 | 0.997 | 118.7 (10.3) | 98.1 (9.5) | 97.4 (9.9) | 0.6 | 2 | 189.5 |
| 207 | Carbophenothion | 21.79 | 125 > 97 (6) | 199 > 143 (10) | 10-100 | 0.9997 | 68.9 (4.7) | 97.8 (7.2) | 91 (8.5) | 3 | 10 | 147.5 |
| 208 | Edifenphos | 21.88 | 109 >65 (13) | 173 >109 (8) | 5-100 | 0.9963 | 91 (4.8) | 102 (6.4) | 97.4 (7.2) | 1.5 | 5 | 106.2 |
| 209 | Lenacil | 21.94 | 136 > 53 (18) | 153 > 136 (14) | 5-100 | 0.9991 | 86 (2.9) | 98.4 (6.5) | 97.2 (8.8) | 1.5 | 5 | 109.9 |
| 210 | Endosulfan sulfate | 22.00 | 241 > 206 (8) | 272 > 237 (10) | 5-100 | 0.9994 | 71.6 (4.8) | 101 (7.2) | 93.9 (7.9) | 1.5 | 5 | 4 |
| 211 | Rabenzazole | 22.00 | 212 > 170 (10) | 212 > 118 (8) | 5-100 | 0.9983 | 92.5 (6.9) | 97.3 (7.9) | 94 (10.9) | 1.5 | 5 | -19.9 |
| 212 | Methoprene | 22.01 | 310 > 73 (25) | 310 > 43 (28) | 5-100 | 0.9982 | 90.4 (5.8) | 94.4 (6.7) | 95.1 (9.2) | 1.5 | 5 | -15.7 |
| 213 | DDT, p,p'- | 22.05 | 235 > 165 (21) | 237 > 165 (22) | 1-100 | 0.9956 | 92.1 (6.6) | 98.8 (7.4) | 94.1 (9.3) | 0.3 | 1 | 45.9 |
| 214 | Hexazinone | 22.12 | 171 > 71 (16) | 171 > 85 (16) | 2-100 | 0.9952 | 92.6 (5.1) | 100 (6.6) | 94 (8.6) | 0.6 | 2 | 124.7 |
| 215 | 2,4'-Methoxychlor | 22.15 | 121 >78 (20) | 227 >121 (12) | 2-100 | 0.9974 | 89.8 (5.8) | 99.6 (6.9) | 94.4 (10.1) | 0.6 | 2 | 36.7 |
| 216 | Heptachlor-exo-epoxide | 22.15 | 390 > 318 (8) | 390 > 237 (15) | 1-100 | 0.9989 | 90.7 (6.6) | 97.9 (7.1) | 96.2 (7.6) | 0.3 | 1 | 16.8 |
| 217 | Nitrothal-Isopropyl | 22.15 | 236 > 194 (8) | 194 > 120 (18) | 1-100 | 0.9983 | 92.4 (5.2) | 99.6 (8) | 94.9 (8.6) | 0.3 | 1 | 14.4 |
| 218 | Captafol | 22.33 | 150 > 79 (6) | 150 > 77 (24) | 10-100 | 0.9993 | 90.7 (5.9) | 70.9 (8.1) | 93.6 (9.3) | 3 | 10 | 124 |
| 219 | Tebuconazole | 22.35 | 125 > 89 (16) | 250 > 125 (22) | 5-100 | 0.9963 | 90.7 (6.2) | 96.7 (7.2) | 95.1 (10.3) | 1.5 | 5 | 92.1 |
| 220 | Propargite | 22.37 | 135 > 77 (24) | 135 > 107 (14) | 10-100 | 0.9982 | 86.8 (5.9) | 99 (7.4) | 92.7 (8.5) | 3 | 10 | 45.8 |
| 221 | Nitralin | 22.44 | 274 > 216 (6) | 316 > 274 (8) | 5-100 | 0.9975 | 92.9 (5.3) | 96.8 (7.6) | 94.3 (7.3) | 1.5 | 5 | 65 |
| 222 | Piperonyl butoxide | 22.45 | 176 > 103 (26) | 176 > 13 (14) | 1-100 | 0.9996 | 90 (5.4) | 95.8 (8.5) | 95.5 (8.6) | 0.3 | 1 | 75.8 |
| 223 | Isofenphos-oxon | 22.58 | 213 > 121 (14) | 185 > 121 (10) | 1-100 | 0.9979 | 93.5 (3.4) | 98.6 (6.5) | 94.4 (10.4) | 0.3 | 1 | 8.6 |
| 224 | Allethrin | 22.63 | 123 > 81 (8) | 123 > 41 (24) | 1-100 | 0.9994 | 88.8 (6.7) | 101.7 (5.6) | 96 (7.5) | 0.3 | 1 | -8.9 |
| 225 | 2,4'-DDE | 22.75 | 246 > 176 (28) | 317 > 246 (20) | 1-100 | 0.9984 | 87 (6) | 95 (7.8) | 93 (8.3) | 0.3 | 1 | 11.6 |

| | | | | | | | | | | | |
|---|---|---|---|---|---|---|---|---|---|---|---|
| 226 | Proquinazid | 22.84 | 288 > 245 (15) | 330 > 288 (5) | 2-100 | 0.9969 | 90.8 (5.3) | 94.5 (6.3) | 97.4 (9.5) | 0.6 | 2 | 16.6 |
| 227 | Iprodione | 22.85 | 314 > 245 (14) | 314 > 271 (12) | 5-100 | 0.9996 | 92.8 (5.5) | 96.6 (5.4) | 96.2 (7.9) | 1.5 | 5 | 111 |
| 228 | Pyridaphenthion | 22.85 | 340 > 109 (20) | 340 > 199 (8) | 5-100 | 0.997 | 90.3 (4.9) | 97.4 (6.4) | 96 (9.8) | 1.5 | 5 | 180.6 |
| 229 | Dicapthon | 22.87 | 262 > 216 (16) | 262 > 93 (32) | 1-100 | 0.9985 | 89.3 (6) | 97.4 (5.1) | 100.1 (5) | 0.3 | 1 | -6.4 |
| 230 | Dimethametryn | 22.91 | 212 > 68 (30) | 212 > 94 (20) | 1-100 | 0.9996 | 87.9 (6.7) | 99.4 (5.5) | 96.5 (6.3) | 0.3 | 1 | 10.4 |
| 231 | Bifenthrin | 23.03 | 181 > 165 (25) | 181 > 166 (10) | 5-100 | 0.9952 | 92.5 (5.6) | 96.1 (5.5) | 93.8 (11.3) | 1.5 | 5 | 50.4 |
| 232 | Phosmet | 23.08 | 160 > 77 (24) | 160 > 133 (10) | 1-100 | 0.9958 | 90.3 (5.7) | 101.5 (5.1) | 93.6 (9.1) | 0.3 | 1 | 155.7 |
| 233 | Endrin ketone | 23.09 | 315 > 279 (8) | 317 > 281 (8) | 5-100 | 0.9961 | 89.8 (5.9) | 98.3 (9.2) | 92 (10.1) | 1.5 | 5 | 6.63 |
| 234 | EPN | 23.11 | 169 > 77 (22) | 169 > 141 (8) | 5-100 | 0.9973 | 90.3 (5.4) | 99.5 (6.5) | 98.6 (9.4) | 1.5 | 5 | 141.1 |
| 235 | Bromopropylate | 23.16 | 183 > 155 (12) | 341 > 183 (15) | 2-100 | 0.9959 | 88.8 (4.3) | 98.2 (7.6) | 93 (9.9) | 0.6 | 2 | 29.3 |
| 236 | 1-Naphthyl Acetamide | 23.24 | 185 > 141 (15) | 185 > 115 (10) | 5-100 | 0.9998 | 85.4 (3.1) | 97.5 (8.6) | 93.2 (8.6) | 1.5 | 5 | 19.9 |
| 237 | Methoxychlor | 23.25 | 227 > 141 (33) | 227 > 169 (25) | 2-100 | 0.9966 | 87 (5.9) | 92.4 (6.3) | 92.3 (4.8) | 0.6 | 2 | 50.7 |
| 238 | Fenpropathrin | 23.26 | 181 > 152 (24) | 265 > 210 (8) | 2-100 | 0.9961 | 88.9 (6.1) | 99.6 (7.2) | 92.2 (8.1) | 0.6 | 2 | 46.3 |
| 239 | Crufomate | 23.28 | 256 > 226 (20) | 169 > 141 (10) | 5-100 | 0.9991 | 89.1 (4.8) | 98.9 (7.7) | 94.5 (9.9) | 1.5 | 5 | 11.2 |
| 240 | Tebufenpyrad | 23.39 | 276 > 171 (10) | 333 > 276 (8) | 2-100 | 0.9986 | 89.9 (4.8) | 98.8 (6.5) | 93.4 (7) | 0.6 | 2 | 16.3 |
| 241 | Fenoxaprop-p | 23.41 | 288 > 119 (10) | 361 > 288 (5) | 5-100 | 0.9978 | 91.9 (6.9) | 95.5 (7.1) | 97.9 (6.5) | 1.5 | 5 | 16.6 |
| 242 | Fluensulfone | 23.59 | 291 > 236 (120 | 264 > 142 (24) | 10-100 | 0.9989 | 88.3 (5.5) | 97.7 (8.2) | 92.8 (7.3) | 3 | 10 | 12.4 |
| 243 | Tetradifon | 23.75 | 159 > 111 (20) | 159 > 131 (10) | 5-100 | 0.9951 | 88.8 (6.9) | 94.4 (7.4) | 93.3 (10.3) | 1.5 | 5 | 52.5 |
| 244 | Chlorfenethol | 23.80 | 251 > 139 (16) | 251 > 43 (23) | 1-100 | 0.9995 | 89.9 (6.4) | 98.7 (8) | 96.8 (10.8) | 0.3 | 1 | 17.4 |
| 245 | Phosalone | 23.87 | 182 > 111 (16) | 182 > 138 (8) | 2-100 | 0.9963 | 87.3 (5.1) | 97 (8.2) | 94.6 (10) | 0.6 | 2 | 166.9 |
| 246 | Leptophos | 23.91 | 171 > 77 (18) | 171 > 124 (8) | 2-100 | 0.9955 | 89 (5.2) | 95.3 (7.3) | 92.7 (9.1) | 0.6 | 2 | 65.1 |
| 247 | Azinphos-methyl | 23.98 | 160 > 77 (16) | 160 > 132 (6) | 5-100 | 0.9956 | 90.6 (6.5) | 99.9 (7.6) | 98 (10) | 1.5 | 5 | 119.7 |
| 248 | Pyriproxyfen | 24.04 | 136 > 78 (20) | 136 > 96 (12) | 5-100 | 0.9995 | 88.4 (5.7) | 98.4 (6.5) | 94.3 (7.9) | 1.5 | 5 | 199.1 |
| 249 | Acrinathrin | 24.17 | 181 > 152 (22) | 208 > 181 (8) | 1-100 | 0.9951 | 93 (5.9) | 98.3 (7.7) | 96.1 (6.7) | 0.3 | 1 | 71.8 |
| 250 | Cyhalothrin, lambda- | 24.18 | 197 > 141 (10) | 208 > 181 (8) | 1-100 | 0.9968 | 92.1 (6.4) | 100.7 (9.3) | 93.6 (6.6) | 0.3 | 1 | 68.7 |
| 251 | Dinobuton | 24.20 | 211 > 163 (8) | 211 > 88 (28) | 10-100 | 0.9998 | 88.8 (5.3) | 101.2 (8.4) | 95.7 (9) | 3 | 10 | -15.5 |
| 252 | Flumetralin | 24.33 | 143 > 107 (18) | 143 > 57 (34) | 10-100 | 0.9985 | 92.5 (6.4) | 94.6 (5.8) | 93.6 (8.5) | 3 | 10 | 19.8 |
| 253 | Pyrazophos | 24.42 | 221 > 193 (8) | 232 > 204 (8) | 1-100 | 0.9996 | 92.5 (6) | 97 (7.1) | 96.7 (9) | 0.3 | 1 | 276 |
| 254 | Iodofenphos | 24.50 | 413 > 377 (8) | 413 > 125 (17) | 5-100 | 0.9965 | 87.5 (3.6) | 98.2 (6.4) | 95 (8.4) | 1.5 | 5 | 14.7 |
| 255 | Mirex | 24.51 | 272 > 237 (15) | 274 > 239 (15) | 5-100 | 0.9959 | 88.8 (5.8) | 96.7 (8.9) | 95.8 (9.2) | 1.5 | 5 | -9.39 |
| 256 | Fenarimol | 24.53 | 139 > 111 (16) | 219 > 107 (12) | 2-100 | 0.9953 | 89 (6.1) | 95 (6.4) | 96.2 (11.9) | 0.6 | 2 | 141.4 |

| | | | | | | | | | | | | | |
|---|---|---|---|---|---|---|---|---|---|---|---|---|---|
| 257 | Terbufos-Sulfone | 24.65 | 153 > 97 (10) | 125 > 97 (6) | 10-100 | 0.9994 | 92.9 (5.3) | 95.3 (7.6) | 90.4 (9.3) | 3 | 10 | 16.9 |
| 258 | Azinphos-ethyl | 24.67 | 132 > 77 (12) | 160 > 132 (6) | 5-100 | 0.9959 | 93.7 (4.9) | 97.1 (8.1) | 95.8 (9.3) | 1.5 | 5 | 108 |
| 259 | Pyraclofos | 24.80 | 194 > 138 (18) | 360 > 194 (10) | 2-100 | 0.9987 | 90.8 (6.9) | 96 (8.8) | 95 (9.7) | 0.6 | 2 | 119.5 |
| 260 | Methidathion | 24.84 | 145 > 85 (6) | 302 > 284 (14) | 5-100 | 0.9997 | 90 (5.9) | 97.1 (6.7) | 91.6 (7.3) | 1.5 | 5 | 16.6 |
| 261 | Flurochloridone | 25.12 | 312 > 187 (14) | 312 > 145 (16) | 1-100 | 0.9972 | 89.1 (5.8) | 100 (6.9) | 95.8 (7.1) | 0.3 | 1 | 10.5 |
| 262 | 2,4'-DDD | 25.14 | 235 > 165 (20) | 235 > 199 (14) | 1-100 | 0.9992 | 91.4 (6.4) | 94 (8) | 92.2 (9.8) | 0.3 | 1 | 17.5 |
| 263 | Permethrin, cis- | 25.19 | 163 > 127 (6) | 183 > 168 (10) | 2-100 | 0.9997 | 91 (6.5) | 97.8 (7.2) | 96.2 (10.5) | 0.6 | 2 | 192.2 |
| 264 | Permethrin, trans- | 25.34 | 163 > 127 (6) | 183 > 153 (14) | 5-100 | 0.9957 | 90.6 (6.8) | 99.3 (9) | 95.2 (7.1) | 1.5 | 5 | 177 |
| 265 | Coumaphos | 25.36 | 210 > 182 (10) | 362 > 109 (17) | 5-100 | 0.9994 | 88.8 (5.3) | 99.6 (8.8) | 98.7 (8.7) | 1.5 | 5 | 190 |
| 266 | Fluquinconazole | 25.38 | 340 > 108 (42) | 340 > 298 (14) | 2-100 | 0.9984 | 91.3 (5.6) | 99.4 (8.3) | 99 (8.3) | 0.6 | 2 | 151.1 |
| 267 | Pyridaben | 25.42 | 147 > 117 (20) | 147 > 132 (13) | 2-100 | 0.9994 | 90.3 (4.7) | 97.8 (9.3) | 95.4 (8.1) | 0.6 | 2 | 122.9 |
| 268 | Prochloraz | 25.43 | 180 > 138 (12) | 308 > 70 (13) | 5-100 | 0.9996 | 91.4 (4.8) | 96.2 (5.5) | 95.1 (9.3) | 1.5 | 5 | 173.2 |
| 269 | Diflufenican | 25.49 | 394 > 266 (15) | 265 > 246 (10) | 1-100 | 0.9987 | 90.4 (6.4) | 96.8 (7.1) | 95.1 (7.2) | 0.3 | 1 | 14.7 |
| 270 | Chlordecone | 25.61 | 271 > 236 (12) | 271 > 140 (36) | 10-100 | 0.9964 | 88.2 (5.5) | 94.9 (6.7) | 95.4 (9) | 3 | 10 | 6.7 |
| 271 | Diclocymet | 25.61 | 277 > 221 (6) | 277 > 112 (12) | 1-100 | 0.9997 | 88.2 (6.3) | 91.4 (6.5) | 92.6 (8.5) | 0.3 | 1 | 5.4 |
| 272 | Pyflubumide | 25.67 | 535 > 155 (22) | 535 > 111 (10) | 5-100 | 0.9994 | 87.7 (5.9) | 97.5 (5.8) | 95.6 (9.2) | 1.5 | 5 | -8.6 |
| 273 | 4,4'-Dibromobenzophenone | 25.77 | 380 > 173 (15) | 380 > 76 (24) | 1-100 | 0.9992 | 88.1 (4.2) | 96.4 (6.3) | 98.3 (10.1) | 0.3 | 1 | -7.8 |
| 274 | Imazamethabenz-methyl | 25.82 | 144 > 116 (10) | 245 > 144 (18) | 1-100 | 0.9965 | 92.4 (6.6) | 99 (8.1) | 92.6 (6.5) | 0.3 | 1 | -6.8 |
| 275 | Tetrasul | 25.99 | 251 > 181 (32) | 251 > 173 (34) | 1-100 | 0.9998 | 90.4 (3.9) | 97.5 (6.6) | 93.5 (8.1) | 0.3 | 1 | 11.9 |
| 276 | Phosfolan | 26.10 | 168 > 140 (10) | 139 > 60 (15) | 5-100 | 0.9981 | 89.7 (6.5) | 98.6 (8.7) | 96.8 (9.7) | 1.5 | 5 | 10.4 |
| 277 | Chloropropylate | 26.21 | 251 > 139 (14) | 139 > 74 (16) | 5-100 | 0.9992 | 89.4 (6.8) | 95.1 (5.8) | 94 (8.5) | 1.5 | 5 | -12.4 |
| 278 | Etofenprox | 26.62 | 163 > 107 (18) | 163 > 135 (10) | 5-100 | 0.9983 | 89.6 (6.3) | 101.9 (5.9) | 98.7 (9.7) | 1.5 | 5 | 199.8 |
| 279 | Isoxathion | 26.76 | 105 > 77 (12) | 105 > 51 (28) | 10-100 | 0.9992 | 89.6 (6.9) | 98.4 (5.7) | 95.1 (9.9) | 3 | 10 | 16.3 |
| 280 | Fluridone | 26.87 | 328 > 189 (38) | 328 > 259 (24) | 5-100 | 0.9961 | 93.4 (5.2) | 97.5 (9) | 93.6 (7.7) | 1.5 | 5 | 14.2 |
| 281 | 4,4'-DDD | 26.94 | 235 > 165 (20) | 235 > 199 (14) | 1-100 | 0.9973 | 88.7 (4) | 98.1 (6.2) | 96.6 (8.2) | 0.3 | 1 | 12.2 |
| 282 | Diofenolan | 27.12 | 300 > 186 (10) | 225 > 103 (34) | 5-100 | 0.9994 | 88.7 (5.1) | 102.5 (5) | 93 (10.2) | 1.5 | 5 | 16.6 |
| 283 | Flurtamone | 27.29 | 333 > 120 (20) | 199 > 157 (15) | 5-100 | 0.9978 | 68.2 (4.3) | 97.8 (7.9) | 97.3 (10) | 1.5 | 5 | 17.7 |
| 284 | Fluorodifen | 27.50 | 190 > 126 (8) | 126 > 75 (12) | 5-100 | 0.9964 | 93.2 (5) | 97 (7.3) | 94.1 (8.5) | 1.5 | 5 | 10.5 |
| 285 | Fluoroglycofen-ethyl | 27.50 | 447 > 223 (14) | 447 > 203 (21) | 5-100 | 0.9986 | 87.8 (5.7) | 94.2 (4.2) | 91 (7.6) | 1.5 | 5 | 19.8 |
| 286 | DEET | 27.55 | 119 > 91 (14) | 191 > 190 (6) | 2-100 | 0.9985 | 93.6 (4) | 98.8 (7.9) | 99.2 (8.9) | 0.6 | 2 | 16.5 |
| 287 | Diclofop-methyl | 28.45 | 252 > 161 (16) | 340 > 253 (10) | 1-100 | 0.9982 | 86.9 (5.6) | 94.9 (7.8) | 92.5 (8.5) | 0.3 | 1 | 10.4 |

| | | | | | | | | | | | | | |
|---|---|---|---|---|---|---|---|---|---|---|---|---|---|
| 288 | Endrin-aldehyde | 28.51 | 173 > 138 (16) | 249 > 214 (24) | 5-100 | 0.9997 | 91.1 (4.6) | 94.7 (5.8) | 71.1 (6.4) | 1.5 | 5 | 18.7 |
| 289 | Fluotrimazol | 28.53 | 310 > 165 (18) | 165 > 115 (26) | 5-100 | 0.9983 | 70.8 (6.1) | 99.5 (8.3) | 94.1 (9.2) | 1.5 | 5 | 14.6 |
| 290 | Fluroxypyr-mepthyl | 28.90 | 180 > 161 (10) | 180 > 134 (20) | 1-100 | 0.9994 | 89.2 (6.7) | 99.2 (7.8) | 89.9 (6.8) | 0.3 | 1 | -12.5 |
| 291 | Cyprofuram | 28.95 | 211 > 132 (12) | 111 > 74 (12) | 5-100 | 0.9976 | 87.6 (6.3) | 98.3 (6.6) | 98.9 (8.1) | 1.5 | 5 | 19.6 |
| 292 | Cyanofenphos | 29.05 | 169 > 141 (8) | 157 > 77 (22) | 1-100 | 0.9992 | 89 (5) | 94.3 (7.9) | 95.9 (8.4) | 0.3 | 1 | 12.8 |
| 293 | Thenylchlor | 29.17 | 127 > 59 (10) | 127 > 53 (18) | 1-100 | 0.9978 | 87.2 (6.8) | 78.8 (8.9) | 91.7 (7.9) | 0.3 | 1 | 16.3 |
| 294 | Genite | 29.50 | 141 > 77 (8) | 141 > 51 (32) | 2-100 | 0.9994 | 88.5 (5.5) | 95.8 (5.5) | 97.3 (9.4) | 0.6 | 2 | 14.6 |
| 295 | Picolinafen | 29.51 | 375 > 238 (20) | 377 > 239 (15) | 2-100 | 0.9964 | 70.4 (5.4) | 101.3 (7.6) | 95 (7.4) | 0.6 | 2 | 10.2 |
| 296 | Mefenpyr-diethyl | 29.59 | 299 > 253 (10) | 253 > 127 (36) | 1-100 | 0.9978 | 89 (4.5) | 93.8 (5.3) | 69.3 (6.2) | 0.3 | 1 | 10.4 |
| 297 | Trifenmorph | 30.41 | 297 > 128 (12) | 297 > 43 (20) | 10-100 | 0.9982 | 92.5 (6) | 101 (6.6) | 94.8 (9.4) | 3 | 10 | 15.9 |
| 298 | Fenoxaprop-Ethyl | 31.84 | 361 > 288 (8) | 119 > 91 (8) | 5-100 | 0.9966 | 90.5 (6.2) | 99.3 (8.2) | 95.1 (8.7) | 1.5 | 5 | 10.4 |
| 299 | Lactofen | 32.14 | 343 > 223 (14) | 223 > 131 (20) | 10-100 | 0.9982 | 87.2 (5.1) | 95.8 (7.1) | 93.7 (7) | 3 | 10 | 11.4 |
| 300 | Tralkoxydim | 32.25 | 137 > 57 (10) | 109 > 57 (6) | 5-100 | 0.999 | 88.8 (6.3) | 99 (9) | 93.1 (8.5) | 1.5 | 5 | 13.3 |
| 301 | Dialifos | 32.36 | 208 > 180 (10) | 209 > 182 (10) | 10-100 | 0.9986 | 91.1 (4.9) | 97.8 (7.8) | 96.1 (8) | 3 | 10 | 14.2 |
| 302 | Fenpiclonil | 32.89 | 236 > 201 (10) | 201 > 165 (140 | 1-100 | 0.9988 | 92.3 (5.1) | 97 (7.6) | 97 (8.1) | 0.3 | 1 | 12 |
| 303 | Silafluofen | 33.38 | 258 > 181 (16) | 258 > 243 (14) | 1-100 | 0.9982 | 92.4 (5.6) | 99.7 (7.4) | 97.4 (12) | 0.3 | 1 | -16.3 |
| 304 | Quizalofop-Ethyl | 33.43 | 372 > 299 (10) | 163 > 99 (20) | 1-100 | 0.9993 | 90.5 (4.7) | 73.7 (10) | 97.6 (9.3) | 0.3 | 1 | 18.8 |
| 305 | Flumioxazin | 36.17 | 354 > 326 (8) | 354 > 176 (16) | 5-100 | 0.9998 | 68.7 (5.1) | 100.1 (7) | 93.2 (7.3) | 1.5 | 5 | 10.6 |
| 306 | Cinidon-Ethyl | 39.66 | 358 > 330 (10) | 330 > 302 (15) | 5-100 | 0.9992 | 88.4 (6.5) | 100.6 (4.8) | 94.7 (10.1) | 1.5 | 5 | 17.5 |
| 307 | Diallate (*) | 14.32, 14.57 | 234 > 150 (18) | 234 > 192 (12) | 5-100 | 0.9986 | 92.2 (5.8) | 97.9 (6.1) | 71.5 (6.1) | 1.5 | 5 | 10.5 |
| 308 | MGK 264 (*) | 18.44, 18.74 | 164 > 67 (10) | 164 > 98 (14) | 5-100 | 0.9977 | 91.5 (6.5) | 96.6 (7.1) | 96 (8.3) | 1.5 | 5 | 14.1 |
| 309 | Chlorfenvinphos (*) | 18.87, 19.5 | 267 > 159 (15) | 323 > 267 (10) | 5-100 | 0.9963 | 88.5 (6.5) | 98.8 (7.2) | 93.5 (8.6) | 1.5 | 5 | 19.8 |
| 310 | Resmethrin (*) | 22.37, 22.51 | 143 > 128 (10) | 171 > 143 (6) | 1-100 | 0.9988 | 87.2 (5) | 100 (7.1) | 96.6 (8.4) | 0.3 | 1 | 14.4 |
| 311 | Tetramethrin (*) | 22.93, 23.11 | 164 > 77 (25) | 164 > 107 (12) | 5-100 | 0.9983 | 69.2 (7.1) | 99 (7) | 95.9 (11) | 1.5 | 5 | -16.4 |
| 312 | Phenothrin (*) | 23.58, 23.71 | 183 > 153 (14) | 183 > 168 (12) | 5-100 | 0.9996 | 89.1 (5.9) | 98 (6) | 90.1 (7.7) | 1.5 | 5 | -10.4 |
| 313 | Cyfluthrin (*) | 25.8, 25.92, 26, 26.05 | 163 > 91 (14) | 163 > 127 (6) | 5-100 | 0.9987 | 94 (4.3) | 97 (7.5) | 95.4 (7.9) | 1.5 | 5 | 16.4 |
| 314 | Cypermethrin (*) | 26.22, 26.32, 26.4, 26.45 | 181 > 127 (30) | 181 > 152 (25) | 5-100 | 0.9987 | 88.6 (6.4) | 100.9 (6.8) | 96.9 (7.8) | 1.5 | 5 | 17.7 |
| 315 | Flucythrinate (*) | 26.4, 26.63 | 157 > 107 (13) | 199 > 157 (8) | 10-100 | 0.9964 | 90.4 (5.4) | 94.3 (4.4) | 95.8 (8.7) | 3 | 10 | 19.5 |
| 316 | Fenvalerate (*) | 27.3, 27.54 | 167 > 125 (8) | 169 > 127 (10) | 10-100 | 0.9994 | 92.9 (3.9) | 99.6 (6.9) | 93.3 (9.1) | 3 | 10 | 16.4 |
| 317 | tau-Fluvalinate (*) | 27.41, 27.48 | 250 > 55 (23) | 250 > 200 (19) | 10-100 | 0.9998 | 89.6 (6.3) | 101.2 (4.8) | 96.8 (7.4) | 3 | 10 | 18.5 |
| 318 | Deltamethrin (*) | 27.94, 28.17 | 181 > 152 (20) | 253 > 174 (8) | 10-100 | 0.9994 | 90.4 (7.4) | 98.3 (7.2) | 93.7 (9) | 3 | 10 | -12.2 |

(*) Pesticides with more than one isomer, calculated all peaks together.

(**) Surrogate and internal standard compounds.

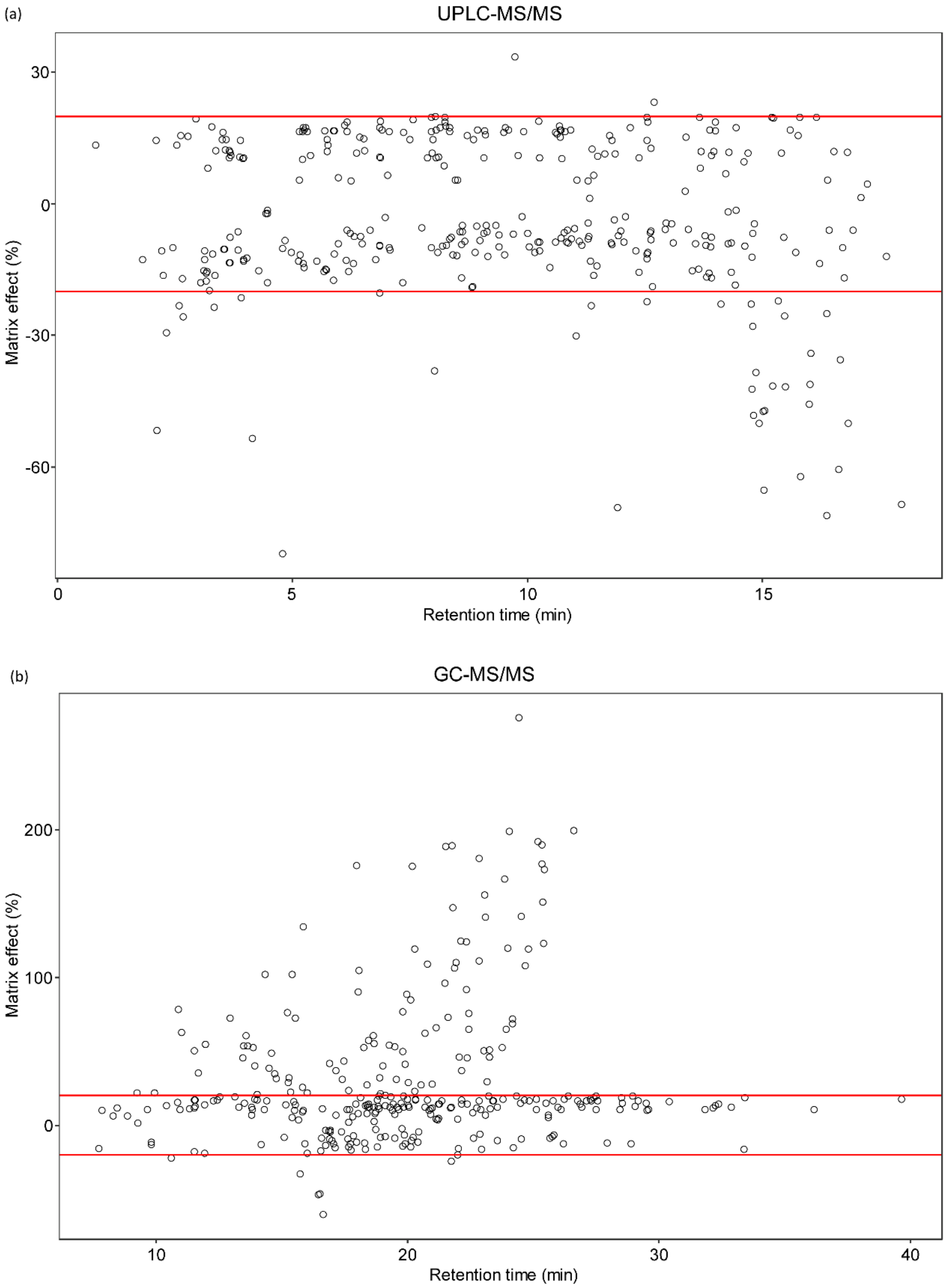


**Figure S1.** Correlation between retention time and matrix effect in (a) UPLC-MS/MS and (b) GC-MS/MS systems.
